# Iron overload in steatotic hepatocytes drives systemic metabolic dysfunction via alterations in hepatokine production

**DOI:** 10.1172/JCI196374

**Published:** 2026-04-28

**Authors:** Hye Jin Jo, Ayoung Kim, Hyunsoo Rho, Ae Kyung Park, Gil-Hwan Kim, Seo Jeong Jo, Hao Yuxin, You-Jung Hong, Ji Min Yeon, Hwang Chan Yu, Mi-Young Song, Jeongwoo Park, Yeon Hee Jeong, Sung Eun Hong, Hyo Jin Yeon, Da Young Oh, Philipp E. Scherer, Cheol Soo Choi, Dong Hyeon Lee, Sung Hwan Ki, Keon Wook Kang, Murim Choi, Byung-Hyun Park, Eun Ju Bae, Sang Geon Kim, Won Kim, Chang Yeob Han

**Affiliations:** 1 School of Pharmacy and Institute of New Drug Development, Jeonbuk National University, Jeonju, Republic of Korea.; 2 College of Pharmacy and Research Institute of Pharmaceutical Sciences, Seoul National University, Seoul, Republic of Korea.; 3 College of Pharmacy and Integrated Research Institute for Drug Development, Dongguk University-Seoul, Goyang-si, Gyeonggi-do, Republic of Korea.; 4 Department of Pathology, Washington University School of Medicine, St. Louis, Missouri, USA.; 5 College of Pharmacy, Graduate School of Pharmaceutical Sciences, and; 6 Graduate Program in Innovative Biomaterials Convergence, Ewha Womans University, Seoul, Republic of Korea.; 7 Graduate School of Medical Science and Engineering, Korea Advanced Institute of Science and Technology, Daejeon, Republic of Korea.; 8 Department of Biochemistry and Molecular Biology, Jeonbuk National University Medical School, Jeonju, Republic of Korea.; 9 Department of Internal Medicine, Seoul National University College of Medicine, Seoul Metropolitan Government Boramae Medical Center, Seoul, Republic of Korea.; 10 Department of Biomedical Sciences, Seoul National University College of Medicine, Seoul, Republic of Korea.; 11 Touchstone Diabetes Center, Department of Internal Medicine, University of Texas Southwestern Medical Center, Dallas, Texas, USA.; 12 Department of Internal Medicine, Gil Medical Center, Gachon University College of Medicine, Incheon, Republic of Korea.; 13 MRC-OSTRC, Research Institute of Pharmaceutical Sciences, College of Pharmacy, Chosun University, Gwangju, Republic of Korea.

**Keywords:** Hepatology, Metabolism, Diabetes, Homeostasis, Obesity

## Abstract

Iron overload has emerged as a significant risk factor for metabolic dysfunction–associated steatotic liver disease (MASLD), a growing global health concern. Despite this association, the precise mechanisms by which hepatic iron and its regulatory genes connect liver pathology to systemic metabolic dysfunction remain elusive. Here, we demonstrate that humoral signals originating from iron-overloaded hepatocytes acted as critical mediators driving systemic metabolic dysfunction in MASLD. Ferroportin (FPN, SLC40A1), the sole cellular iron exporter, exhibited markedly reduced expression in hepatocytes of both patients with MASLD and mouse models of the disease, concomitant with hepatic iron accumulation. Functionally, hepatocyte-specific FPN deletion significantly exacerbated diet-induced obesity and insulin resistance, with these metabolic perturbations accompanied by decreased energy expenditure and impaired thermogenic capacity. Mechanistically, we establish that hepatic iron accumulation resulting from FPN deficiency enhanced the production of 2 specific hepatokines, fetuin-A and LECT2, through activation of the transcription factor FoxO1. Notably, therapeutic interventions — including genetic silencing of these hepatokines, hepatocyte-specific FPN overexpression, or oral iron chelation — effectively reversed the metabolic dysfunction phenotypes. These findings provide critical insights into the pathophysiological mechanisms linking MASLD to systemic metabolic disorders and highlight promising therapeutic strategies to combat these diseases.

## Abstract 2

Iron overload in the liver disrupts systemic metabolism by altering hepatocyte-producing hormone signals, suggesting novel treatment strategies for MASLD and its cardiometabolic complications.

## Graphical abstract

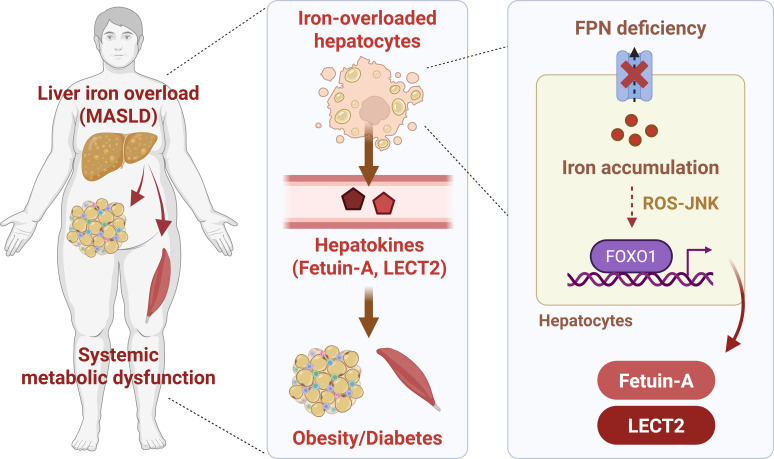

## Introduction

The global prevalence of metabolic dysfunction–associated steatotic liver disease (MASLD), a condition encompassing hepatic steatosis with cardiometabolic risk factors, has risen dramatically in parallel with the obesity epidemic, positioning it as a major public health challenge (1–4). While MASLD is strongly associated with systemic metabolic disorders, including type 2 diabetes (T2D) and cardiovascular disease (5–8), the molecular mechanisms linking hepatic pathology to extrahepatic complications remain poorly understood. This knowledge gap has hindered the development of effective therapies, leaving patients reliant on lifestyle modifications with extremely limited treatment options (5, 9, 10).

The liver serves as a central metabolic hub, coordinating lipid and glucose homeostasis, while functioning as an endocrine organ through the secretion of hepatokines, namely liver-derived signaling proteins that regulate inter-organ communication (7, 11, 12). Clinical and preclinical studies have identified multiple hepatokines whose dysregulated expression correlates with insulin resistance (IR) and metabolic syndrome (7, 11, 12). However, the upstream drivers of pathogenic hepatokine profiles in MASLD remain undefined, posing a critical barrier to therapeutic development.

Iron homeostasis has emerged as a key regulator of metabolic processes beyond its classical roles in oxygen transport and erythropoiesis (13–17). Clinical evidence demonstrates a strong association between iron overload and the risk of metabolic syndrome (14, 18, 19). However, little is known about the underlying mechanisms through which tissue- and cell-type–dependent iron levels influence metabolic disturbances. While excess iron in adipocytes has been shown to impair adipokine signaling and promote obesity (20–22), the metabolic consequences of hepatocyte-specific iron dysregulation remain unexplored. This represents an important gap in our understanding, given the liver’s dual roles in iron homeostasis and metabolic coordination.

Ferroportin (FPN, also known as SLC40A1), the sole mammalian iron exporter, regulates cellular iron homeostasis by mediating iron efflux from enterocytes, macrophages, and hepatocytes (23–25). Though FPN’s functions in dietary iron absorption (enterocytes) and iron recycling (macrophages) are well characterized (23), its hepatocyte-specific roles remain unclear. Hepatic FPN downregulation could theoretically promote intrahepatic iron retention while sparing systemic iron parameters, which is a scenario with unexplored metabolic implications. In particular, the hepatocyte iron/hepatokine axis remains unmapped.

In this study, we bridge these knowledge gaps by investigating how FPN deficiency–induced hepatic iron overload reprograms hepatokine secretion to drive systemic metabolic dysfunction. Through integrated clinical cohort analyses and mechanistic studies in hepatocyte-specific FPN-KO models, we identify an iron/FoxO1/hepatokine signaling axis that links MASLD pathophysiology to its cardiometabolic comorbidities. Our findings provide both biological insights into hepatokine regulation and translational strategies for targeting iron homeostasis in metabolic diseases.

## Results

### Serum ferritin serves as a reliable biomarker for metabolic pathophenotypes in patients with MASLD.

**Figure 1:**
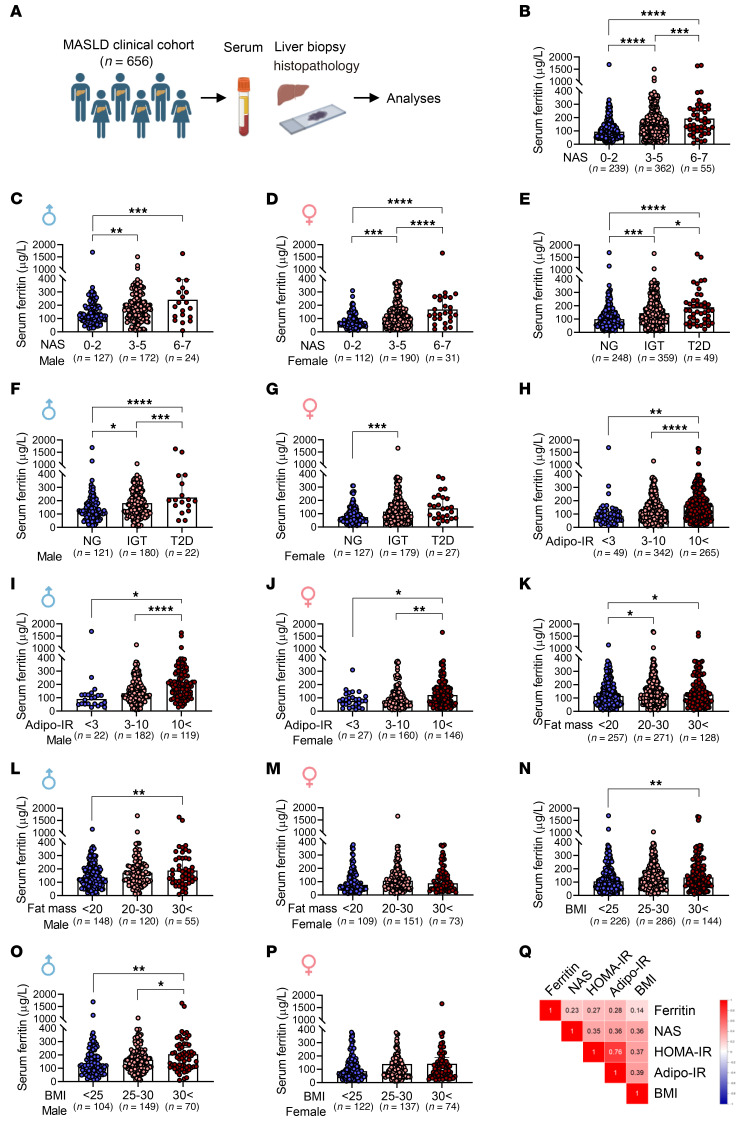
Serum ferritin levels correlate with metabolic parameters in patients with MASLD. (**A**) Overview of correlation analyses between serum ferritin and metabolic parameters in patients with MASLD. (**B**–**P**) Serum ferritin levels were analyzed across patient subgroups stratified by sex and clinical parameters: NAS (**B**–**D**), HOMA-IR (**E**–**G**), adipo-IR (**H**–**J**), fat mass (**K**–**M**), and BMI (**N**–**P**). Subgroups in panels **E**–**G** represent normoglycemia (NG) (HOMA-IR <3), impaired glucose tolerance (IGT) (3~10), and overt T2D (>10). (**Q**) Correlation plot summarizing relationships between serum ferritin and metabolic parameters. Data are presented as the median ± 95% CI. *P < 0.05, **P < 0.01, ***P < 0.001, and ****P < 0.0001, by 1-way ANOVA followed by Tukey’s multiple-comparison test.

To elucidate the relationship between iron homeostasis and metabolic features in clinical cases of MASLD, we conducted a comprehensive analysis of serum ferritin, a marker of iron stores, and its associations with diverse metabolic parameters in a well-characterized MASLD cohort (Figure 1A). Detailed baseline characteristics of this cohort are presented in Supplemental Table 1; supplemental material available online with this article; https://doi.org/10.1172/JCI196374DS1 Our sex-stratified subgroup analyses revealed a significant and progressive increase in ferritin levels corresponding to a rising nonalcoholic fatty liver disease (NAFLD) activity score (NAS) in both male and female patients (Figure 1, B–D). In addition, serum ferritin demonstrated strong correlations with IR, as quantified by the homeostatic model assessment of IR (HOMA-IR) (Figure 1, E–G) and adipose tissue IR (adipo-IR) (Figure 1, H–J). Moreover, both fat mass (Figure 1, K–M) and BMI (Figure 1, N–P) exhibited significant associations with serum ferritin levels, with these relationships being particularly pronounced in male participants. Given the overlap in serum ferritin levels observed in the scatter plots, we reanalyzed the cohort by stratifying patients into ferritin quartiles and confirmed that higher ferritin levels were associated with worse clinical outcomes, including increased NAS, HOMA-IR, and adipo-IR (Supplemental Figure 1).

To further validate serum ferritin as an independent predictor of IR, we performed multiple linear regression analysis using adipo-IR or HOMA-IR as response variables. These analyses were carefully adjusted for potential confounding factors, including demographics (sex, age), obesity parameters (BMI), serum lipid profile (triglycerides), and liver injury markers (alanine transaminase, γ-glutamyl transferase, NAS). Variables with right-skewed distributions were log-transformed, and representative variables were selected to minimize multicollinearity (Supplemental Figure 2A). Some variables were reclassified according to similar mean log-transformed values (Supplemental Table 2). Hepatic pathology adjustments were made using either NAS (model I) or its subcategories such as steatosis, hepatocyte ballooning, and lobular inflammation (model II). Notably, serum ferritin remained a significant independent predictor of IR across all statistical models (Table 1 and Supplemental Table 3). Furthermore, we observed that the association between serum ferritin and IR strengthened with increasing steatosis severity (Supplemental Figure 2B), although this relationship was less evident when analyzed in the context of cellular damage status (Supplemental Figure 2, C–F). Altogether, these findings demonstrate that serum ferritin levels are closely linked to glucose intolerance in patients with MASLD (Figure 1Q), underscoring the potential role of iron as a strong risk factor and highlighting the value of serum ferritin as a prognostic biomarker for the progression of systemic metabolic disturbance associated with MASLD.

### Hepatocyte FPN expression is downregulated in MASLD.

Liver iron content positively correlates with serum ferritin levels in patients with MASLD (26, 27), and hepatic iron overload is frequently observed in both human and murine models of metabolic dysfunction–associated steatohepatitis (MASH) (14, 26). To identify key iron-regulatory molecules linking iron overload to metabolic disturbances, we first conducted a comprehensive screening of genes associated with iron homeostasis, including *FPN*, using established public databases. Among the various genes involved in iron export, import, and systemic regulation, FPN emerged as significantly downregulated in the livers of patients with steatosis compared with healthy individuals (Supplemental Figure 3A). This downregulation pattern was similarly observed in patients with steatohepatitis (Supplemental Figure 3B), with FPN levels substantially lower in individuals with severe MASLD than in those with mild disease (Supplemental Figure 3C).

**Figure 2:**
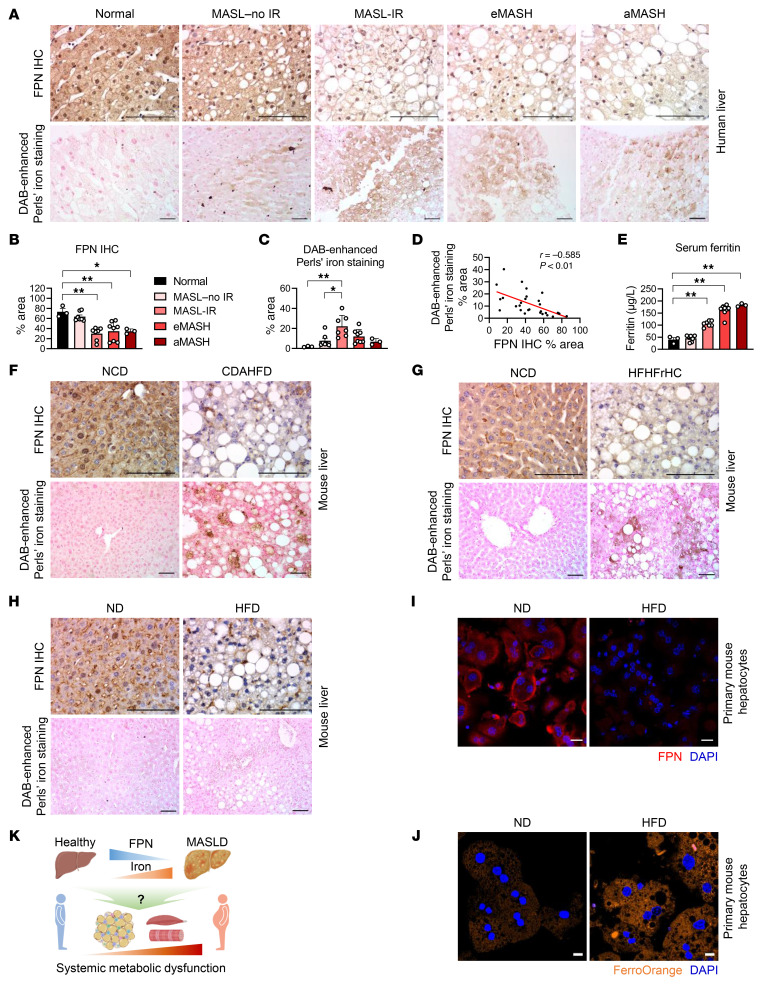
Hepatic FPN expression is downregulated in human and murine MASLD. (**A**–**C**) Immunohistochemical staining for FPN and DAB-enhanced Perls’ iron staining in liver tissues from the MASLD cohort (**A**), with corresponding quantifications (**B** and **C**). Normal controls (*n* = 3); MASL, simple steatosis with or without IR (*n* = 7 per group); eMASH with no or mild fibrosis (F0–1) (*n* = 8); and aMASH with F ≥2 (*n* = 3). (**D**) Inverse correlation between FPN expression and hepatic iron accumulation. Pearson correlation coefficients (*r*) and corresponding *P* values are shown. (**E**) Serum ferritin levels across MASLD subgroups. (**F**–**H**) FPN immunostaining and Perls’ iron staining in mice fed a CDAHFD (**F**), a HFHFrHC diet (**G**), or a standard HFD (**H**). (**I**) IF staining for FPN in primary hepatocytes isolated from HFD-fed mice. Scale bars: 20 μm. (**J**) Live-cell fluorescence imaging of intracellular labile iron levels in primary hepatocytes, as shown in **I**. (**K**) Schematic illustrating the proposed role of hepatic FPN deficiency in MASLD-associated systemic metabolic dysfunction. Data are presented as the mean ± SEM. Scale bars: 50 μm (upper images) and 20 μm (lower images) (**A** and **F**–**H**). *P < 0.05 and **P < 0.01, by 1-way ANOVA followed by Tukey’s multiple-comparison test.

Next, we examined FPN downregulation in greater detail according to metabolic disease severity in patients with MASLD , who were stratified into the following subgroups: steatosis without IR (MASL without IR [MASL–no IR]), steatosis with IR (MASL with IR [MASL-IR]), early MASH (eMASH), and advanced MASH (aMASH). Immunostaining demonstrated that hepatic FPN protein expression was progressively reduced across MASLD stages compared with normal controls, with more pronounced downregulation beginning in MASL-IR and persisting through eMASH and aMASH (Figure 2, A and B). Notably, FPN reduction was modest and not statistically significant in MASL–no IR, whereas more substantial downregulation was observed in metabolically advanced stages.

To directly assess hepatic iron content in the human cohort, we performed 3,3′-diaminobenzidine–enhanced (DAB-enhanced) Perls’ Prussian blue staining and found that hepatic iron accumulation was present across MASLD stages (Figure 2, A and C). The MASL-IR group exhibited greater hepatic iron deposition than the MASL–no IR group and showed the most prominent iron accumulation within hepatocytes. Hepatic FPN expression was inversely associated with iron accumulation (Figure 2D).

Serum ferritin levels increased progressively with advancing disease severity, particularly in MASH stages (Figure 2E), suggesting that circulating ferritin may reflect both hepatic iron burden and inflammatory status in advanced MASLD. In contrast, transferrin receptor 1 (TFRC), a key mediator of iron import, was not significantly altered in patients with MASLD (Supplemental Figure 3D), indicating that increased hepatic iron burden was more closely associated with impaired iron export rather than enhanced iron import.

To validate these clinical observations in experimental models, we examined FPN expression and hepatic iron content in multiple murine models of MASLD. Consistent with the human data, FPN protein levels were significantly reduced in the livers of mice fed a choline-deficient, l-amino acid–defined high-fat diet (CDAHFD), a high-fat, high-fructose, high-cholesterol diet (HFHFrHC diet), or a simple HFD compared with their respective controls (Figure 2, F–H). Hepatic *Fpn* mRNA levels were likewise decreased in CDAHFD-fed and HFD-fed mice (Supplemental Figure 3E). This reduction in FPN expression was further confirmed in primary hepatocytes isolated from HFD-fed mice (Figure 2I and Supplemental Figure 3F).

The downregulation of FPN was accompanied by hepatic iron accumulation in mice fed a CDAHFD or a HFHFrHC diet (Figure 2, F and G). Although hepatic FPN downregulation was also observed in HFD-fed mice, Perls’ staining showed minimal ferric iron (Fe^3+^) deposition under these conditions (Figure 2H) compared with the more robust accumulation seen in the CDAHFD and HFHFrHC models. This difference may reflect variations in disease severity, including inflammatory burden and hepatocellular injury, as well as dietary context, all of which may influence the detectability of ferric iron deposition. Importantly, FerroOrange staining demonstrated that labile ferrous iron (Fe^2+^) levels were elevated in primary hepatocytes isolated from HFD-fed mice (Figure 2J), suggesting a redistribution of intracellular iron toward a redox-active pool despite the limited ferric iron accumulation detectable by Perls’ staining. Collectively, these results suggest that hepatic FPN downregulation in MASLD may contribute to iron overload and associated metabolic disorders, establishing a potential mechanistic link between altered iron homeostasis and metabolic dysfunction (Figure 2K).

### Hepatocyte FPN deficiency exacerbates diet-induced obesity and IR.

**Figure 3:**
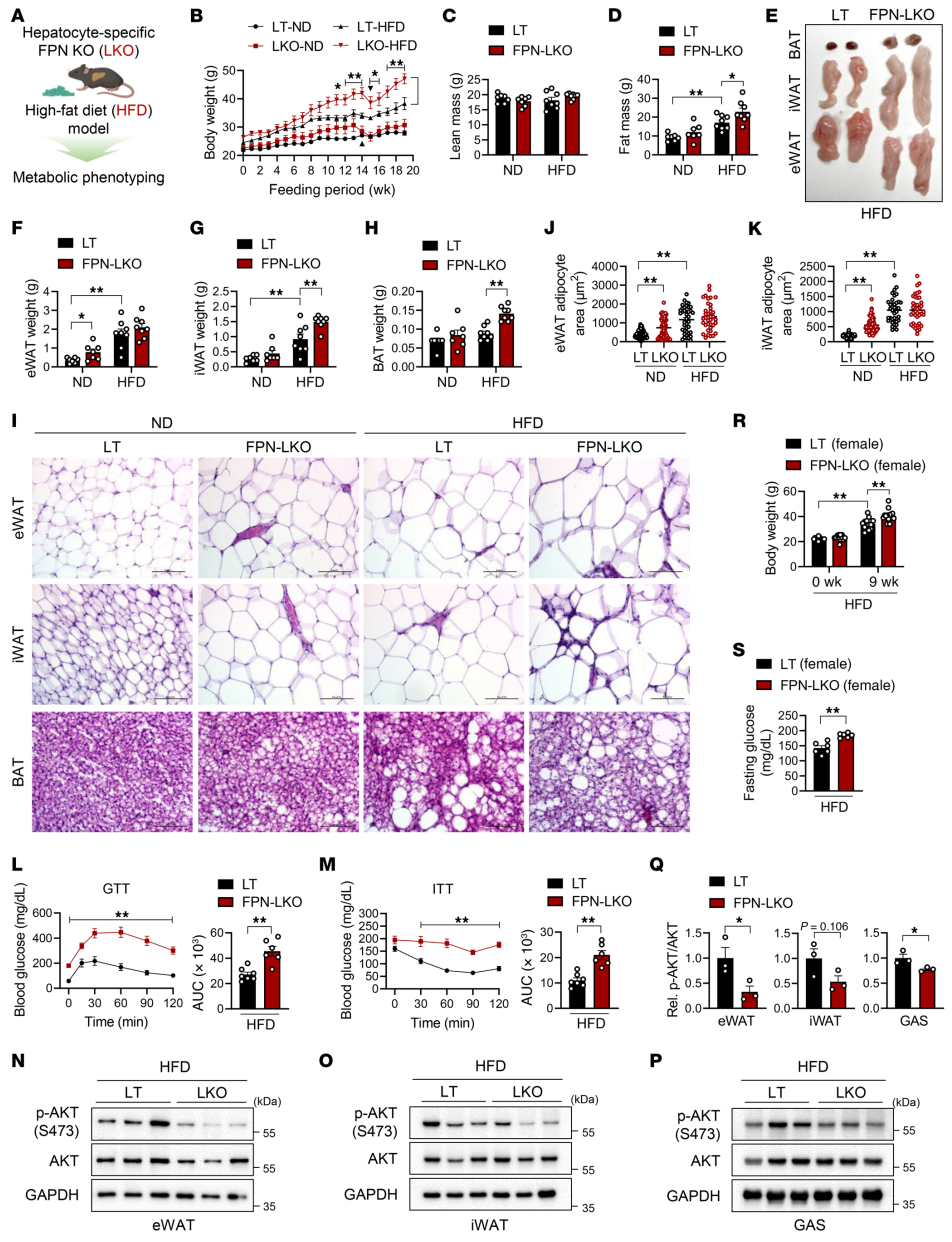
Hepatocyte-specific FPN deletion exacerbates obesity and IR under HFD conditions. (**A**) Study design for metabolic phenotyping of hepatocyte-specific *Fpn*-KO mice fed a HFD. (**B**) Body weight curves of LT control and *Fpn*-LKO mice fed a ND or a HFD for 19 weeks (*n* = 7–8 per group). Arrowheads indicate the timing of GTT and ITT analyses. (**C** and **D**) Body composition analysis measuring lean and fat mass (*n* = 7–8 per group). (**E**–**H**) Representative images of eWAT, iWAT, and BAT from HFD-fed mice and tissue weights of eWAT, iWAT, and BAT (*n* = 7–8 per group). (**I**–**K**) H&E staining of eWAT, iWAT, and BAT. Scale bars: 50 mm. Adipocyte size in H&E-stained sections of eWAT and iWAT (*n* = 3 per group). (**L** and **M**) GTT and ITT analyses in HFD-fed mice (*n* = 6–7 per group). (**N**–**Q**) Western blot analysis of p-Akt (S473) in eWAT, iWAT, and GAS tissues from HFD-fed mice. Relative band intensities were quantified. (**R** and **S**) Body weight (*n* = 12–13 per group) and fasting blood glucose levels (*n* = 6 per group) in female LT and *Fpn*-LKO mice fed a HFD for 9 weeks. Data are presented as the mean ± SEM. *P < 0.05 and **P < 0.01, by unpaired, 2-tailed Student’s *t* test.

To investigate the functional consequences of hepatic FPN downregulation in metabolic disease progression, we generated hepatocyte-specific, *Fpn*-deficient mice (hereafter referred to as *Fpn*-LKO) (Supplemental Figure 4, A and B) and subjected them to a HFD (Figure 3A). Throughout this study, we utilized littermate *Fpn^fl/fl^* mice (hereafter referred to as LT) as appropriate controls. Successful and specific deletion of FPN in hepatocytes was rigorously confirmed in the *Fpn*-LKO mice (Supplemental Figure 4, C–E). IHC staining for FPN provided additional validation of hepatocyte-specific FPN KO and antibody specificity (Supplemental Figure 4F).

On a normal chow diet (ND), *Fpn*-LKO mice exhibited a modest, but not insignificant, increase in body weight compared with LT controls. However, under HFD conditions, *Fpn*-LKO mice gained significantly more body weight relative to controls (Figure 3B). Detailed body composition analyses revealed that total fat mass was substantially higher in HFD-fed *Fpn*-LKO mice, whereas lean body mass remained comparable between the genotypes (Figure 3, C and D). This adiposity phenotype was further evidenced by increased weights of epididymal white adipose tissue (eWAT) and inguinal WAT (iWAT) as well as of brown adipose tissue (BAT) (Figure 3, E–H), with skeletal muscle weights remaining similar between the 2 genotypes (Supplemental Figure 4G). Histological examination demonstrated adipocyte hypertrophy in *Fpn*-LKO mice even under ND conditions (Figure 3, I–K), and elevated adipose tissue inflammation was indicated by increased F4/80 staining and crown-like structure counts (Supplemental Figure 4H).

Glucose tolerance tests (GTTs) and insulin tolerance tests (ITTs) revealed that HFD-fed *Fpn*-LKO mice exhibited pronounced hyperglycemia and markedly reduced insulin sensitivity compared with HFD-fed LT mice (Figure 3, L and M). In ND-fed mice, these metabolic differences were relatively modest (Supplemental Figure 5, A and B). At the molecular level, phosphorylation of Akt (p-Akt), a key indicator of insulin receptor signaling, was significantly diminished in eWAT, iWAT, and gastrocnemius (GAS) muscles of HFD-fed *Fpn*-LKO mice (Figure 3, N–Q). Notably, in the livers of *Fpn*-LKO mice, both p-Akt levels and expression of gluconeogenic enzyme genes (i.e., phosphoenolpyruvate carboxykinase [*Pepck*] and glucose 6-phosphatase [*G6pase*]) remained largely unchanged (Supplemental Figure 5, C and D). Consistently, we observed no significant differences in the expression of key gluconeogenic regulators between genotypes, including cAMP response element–binding protein (CREB), CCAAT/enhancer-binding proteins (C/EBP)-α and C/EBPβ (Supplemental Figure 5E). This suggests that the observed systemic IR primarily resulted from reduced insulin sensitivity in peripheral metabolic tissues, such as adipose tissues and skeletal muscles. Importantly, the increases in body weight gain and fasting blood glucose levels were consistently observed in HFD-fed female *Fpn*-LKO mice (Figure 3, R and S), indicating that these metabolic effects were not sex dependent. Taken together, these findings demonstrate that hepatocyte-specific FPN deficiency significantly promoted obesity and glucose intolerance during the development of diet-induced metabolic disorders.

### Hepatocyte FPN deficiency enhances steatohepatitis development.

We next assessed the effect of hepatocyte FPN deficiency on liver pathophysiology, focusing on lipid metabolism and inflammatory processes. *Fpn*-LKO mice exhibited significant increases in liver weight, serum alanine aminotransferase (ALT) and aspartate aminotransferase (AST) activities, hepatic triglyceride (TG) content, and the number of F4/80^+^ cells compared with controls (Supplemental Figure 6, A–F). To gain mechanistic insights into these alterations, we performed RNA-seq on liver samples from mice of both genotypes. Gene ontology and pathway enrichment analyses identified significant perturbations in genes involved in lipid metabolic processes, inflammatory responses, and cellular stress responses in the livers of *Fpn*-LKO mice compared with controls (Supplemental Figure 6, G and H).

Subsequent quantitative PCR (qPCR) analyses revealed decreased expression of genes related to lipid oxidation, mitochondrial biogenesis, oxidative phosphorylation, and antiinflammatory response in the livers of HFD-fed *Fpn*-LKO mice (Supplemental Figure 7, A and C–E). Interestingly, the expression of lipogenic genes was decreased, while proinflammatory gene expression levels remained largely unaltered (Supplemental Figure 7, B and F). Despite the potential link between iron overload and ferroptosis, we did not observe significant changes in the expression of ferroptosis-associated genes under our experimental conditions (Supplemental Figure 7G). These results suggest that FPN deficiency augments steatohepatitis development primarily through reduced mitochondrial lipid oxidation capacity and diminished expression of antiinflammatory mediators.

### Hepatocyte FPN deficiency reduces energy expenditure and thermogenic capacity.

**Figure 4:**
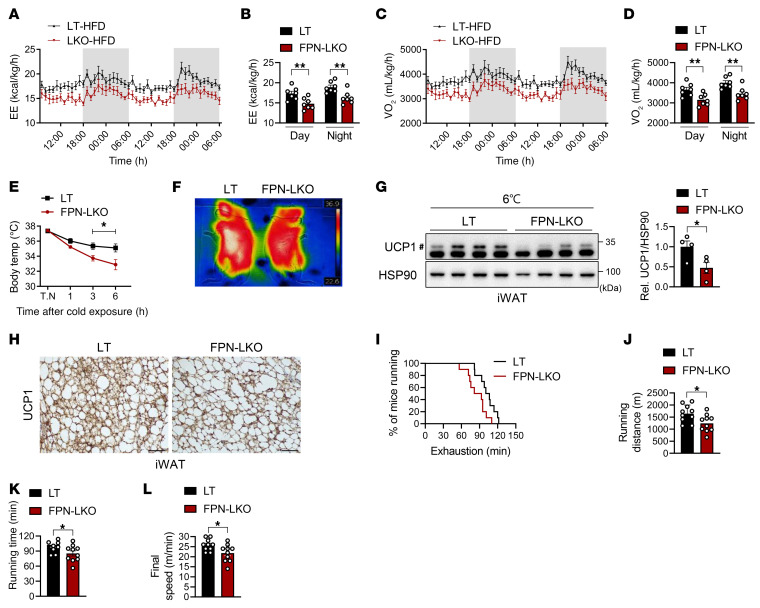
Hepatic FPN deficiency reduces energy expenditure and impairs thermogenic capacity. (**A**–**D**) Comprehensive metabolic cage analyses measuring energy expenditure (EE) (**A** and **B**) and oxygen consumption rates (VO_2_) (**C** and **D**) in LT control versus *Fpn*-LKO mice (*n* = 7 per group) fed a HFD during light/dark cycles. (**E**) Rectal temperature (temp.) measurements in LT and *Fpn*-LKO (*n* = 5 per group) after cold exposure at 6°C for the indicated times. T.N., thermoneutral condition. (**F**) Surface body temperature measurements and representative thermal imaging after cold exposure at 6°C for 48 hours. (**G** and **H**) Western blotting and immunohistochemical staining for UCP1 in iWAT from the mice shown in **F** following cold exposure. The UCP1 band is indicated by a pound sign, and molecular weight markers are shown. Scale bars: 50 μm. Rel., relative. (**I**–**L**) Endurance exercise performance tests assessing running distance, duration, and final speed achieved by LT control versus *Fpn*-LKO mice (*n* = 10 per group) on treadmill exercise protocols. Data are presented as the mean ± SEM. *P < 0.05 and **P < 0.01, by unpaired, 2-tailed Student’s *t* test.

Given the pronounced obesity-related metabolic dysfunction observed in *Fpn*-LKO mice, we conducted comprehensive metabolic phenotyping, including metabolic cage studies. Food intake and physical activity remained comparable between LT control and *Fpn*-LKO mice under both ND and HFD conditions (Supplemental Figure 8, A and B). In contrast, energy expenditure (Figure 4, A and B) and the oxygen consumption rate (VO_2_) (Figure 4, C and D) were significantly lower in *Fpn*-LKO mice compared with LT controls during both the light and dark cycles. These differences were particularly noticeable in HFD-fed mice, whereas similar trends were observed in ND-fed mice, although without reaching statistical significance (Supplemental Figure 8, C–F). Additionally, the respiratory exchange ratio was reduced in ND-fed *Fpn*-LKO mice (Supplemental Figure 8, G and H), but we observed no significant difference in HFD-fed mice (Supplemental Figure 8, I and J).

Since impaired thermogenesis is closely linked to obesity-related metabolic disorders (28), we next evaluated thermogenic responses following cold stress exposure. *Fpn*-LKO mice exhibited significantly lower rectal and surface body temperatures than did control mice upon cold challenge (Figure 4, E and F). At the molecular level, expression of uncoupling protein 1 (UCP1), a key mediator of adaptive thermogenesis, was substantially reduced in iWAT (Figure 4, G and H) but remained unchanged in BAT (Supplemental Figure 8K) of cold-exposed *Fpn*-LKO mice. We validated the specificity of the UCP1 band in iWAT by control experiments (Supplemental Figure 8L).

To assess the functional metabolic outcomes, we further compared exercise performance between LT control and *Fpn*-LKO mice. Endurance exercise testing revealed that *Fpn*-LKO mice had significantly impaired exercise capacity, as evidenced by reduced running distance and duration (Figure 4, I–L). Collectively, these findings support the conclusion that hepatocyte-specific FPN ablation contributed to obesity development through reduced energy expenditure and impaired thermogenic capacity, establishing a link between hepatic iron homeostasis and whole-body energy metabolism.

### Hepatocyte iron accumulation increases fetuin-A and LECT2 expression via FoxO1 activation.

To elucidate the mechanistic basis underlying the metabolic phenotypes driven by hepatic FPN deficiency, we first measured iron levels in metabolic tissues, given FPN’s fundamental role as an iron exporter and the known effects of adipose tissue iron on metabolic dysfunction (22, 23). As anticipated, hepatic iron content and the expression of ferritin heavy chain (FTH) and light chain (FTL) were significantly elevated in the livers of HFD-fed *Fpn*-LKO mice compared with levels in HFD-fed LT control mice (Supplemental Figure 9, A and B). We further confirmed hepatocellular iron accumulation by Perls’ staining (Supplemental Figure 9C). Interestingly, serum iron levels, total iron-binding capacity (TIBC), and transferrin saturation remained unchanged between the genotypes (Supplemental Figure 9, D–F). Hepatic *Hamp1* transcript levels did not differ between LT controls and Fpn-LKO mice under ND conditions. HFD feeding reduced hepatic *Hamp1* expression, and this reduction was further enhanced by FPN deficiency (Supplemental Figure 9G), which may reflect disease progression in advanced MASLD, as hepcidin expression is often reduced in chronic liver disease (29). Notably, iron content in adipose tissues and skeletal muscles was comparable between *Fpn*-LKO and control mice (Supplemental Figure 9, H–J). These results suggest that hepatocyte-specific FPN deficiency, which causes selective liver iron overload, may influence systemic energy metabolism independently of iron level alterations in peripheral metabolic tissues.

On the basis of these observations, we hypothesized that FPN loss in hepatocytes may alter humoral signals, leading to the production of secreted factors involved in metabolic regulation. To investigate this possibility, we isolated primary hepatocytes from HFD-fed LT control and *Fpn*-LKO mice and collected conditioned media (CM), which were then applied to differentiated 3T3-L1 adipocytes or C2C12 myotubes in the presence of insulin (Supplemental Figure 10A). CM of FPN-deficient hepatocytes significantly impaired insulin-stimulated Akt phosphorylation in both adipocytes and myotubes (Supplemental Figure 10, B and D), whereas heat-inactivated CM failed to exert such effects (Supplemental Figure 10, C and E). These results strongly implicate hepatocyte-derived secreted proteins in mediating the observed metabolic disturbances, which directed our attention toward potential hepatokines.

**Figure 5:**
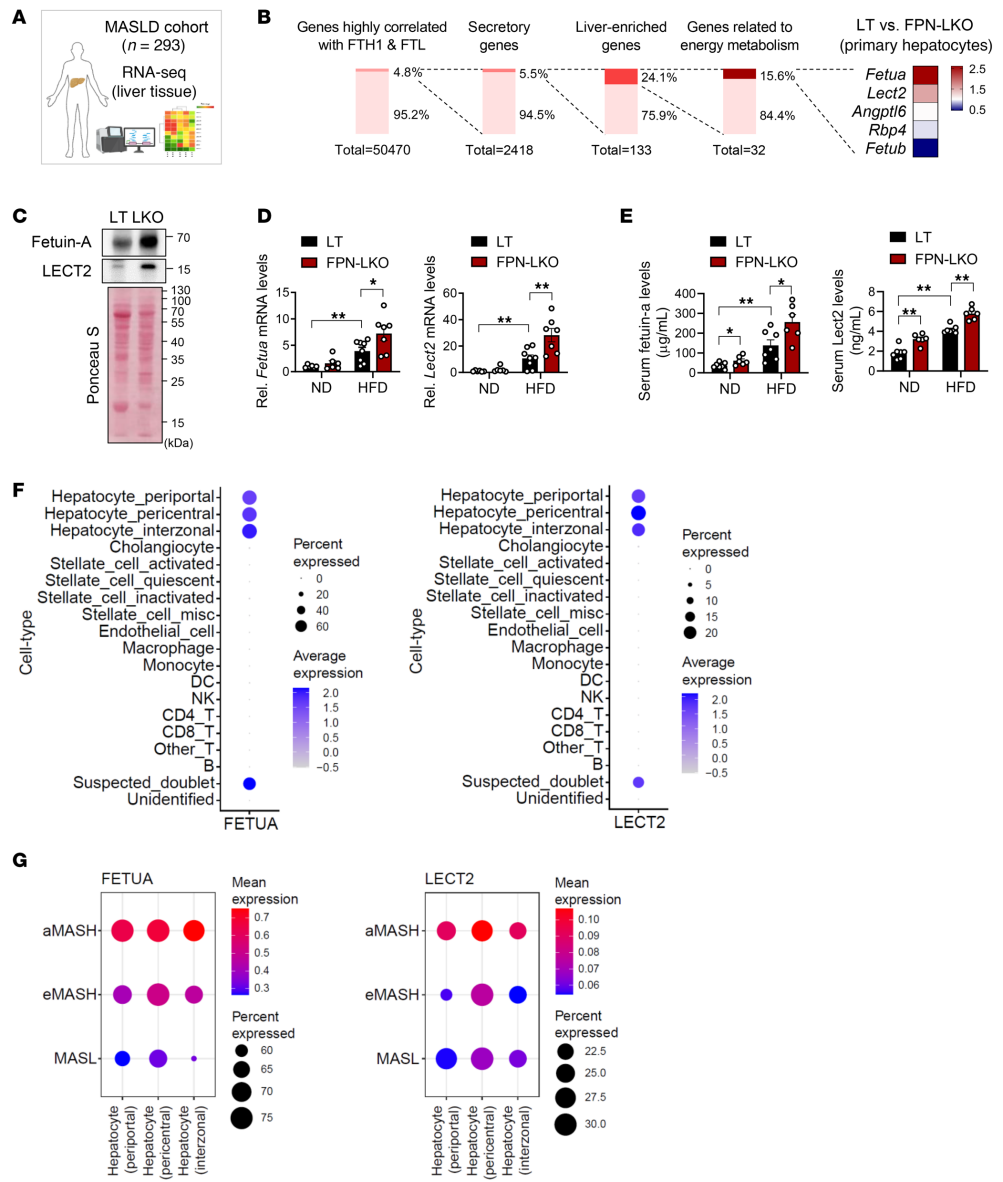
Hepatic FPN deficiency increases the expression of hepatokines, fetuin-A, and LECT2. (**A** and **B**) Overview of bulk RNA-seq analysis strategy used to identify hepatokines associated with hepatic iron overload in MASLD patient liver samples. Sequential filtering criteria are illustrated schematically in **B**. (**C**) Western blot analysis of secreted fetuin-A and LECT2 proteins from CM derived from primary hepatocytes isolated from HFD-fed LT mice versus *Fpn*-LKO mice. (**D** and **E**) Hepatic mRNA expression and serum levels of fetuin-A and LECT2 in ND- versus HFD-fed LT and *Fpn*-LKO mice (*n* = 6–8 per group). (**F**) snRNA-seq analyses demonstrating cell-type–specific expression patterns of fetuin-A and LECT2 across human liver cells. (**G**) snRNA-seq analyses depicting fetuin-A and LECT2 expression levels in hepatocytes from patients with MASLD according to MASLD severity. Data are presented as the mean ± SEM. *P < 0.05 and **P < 0.01, by unpaired, 2-tailed Student’s *t* test.

To identify key hepatokines involved in this process, we performed RNA-seq analysis of liver tissues from patients with MASLD (Figure 5A). We implemented a systematic approach to identify candidate factors, initially selecting genes strongly correlated with ferritin expression and then sequentially refining the candidates according to secretion potential, liver-specific expression, and established roles in energy metabolism (Figure 5B). Among the final 5 hepatokine candidates, fetuin-A (*Fetua*) and leukocyte cell-derived chemotaxin 2 (*Lect2*) were significantly upregulated in FPN-deficient hepatocytes (Supplemental Figure 11A). Increased production and secretion of fetuin-A and LECT2 proteins were validated in CM from these hepatocytes (Figure 5C). Consistently, hepatic expression of *Fetua* and *Lect2* was markedly increased by HFD feeding compared with a ND, and this induction was exacerbated by hepatic FPN deficiency (Figure 5D). This pattern was corroborated by measurements of serum levels of these hepatokines (Figure 5E), suggesting a combined contribution of steatotic stress and iron overload to fetuin-A and LECT2 induction. Single-nucleus RNA-seq (snRNA-seq) further revealed hepatocyte-specific expression patterns for fetuin-A and LECT2 (Figure 5F), with their expression levels increasing proportionally with disease severity in patients with MASLD (Figure 5G), in parallel with increased hepatocyte ferritin levels (Supplemental Figure 11B).

**Figure 6:**
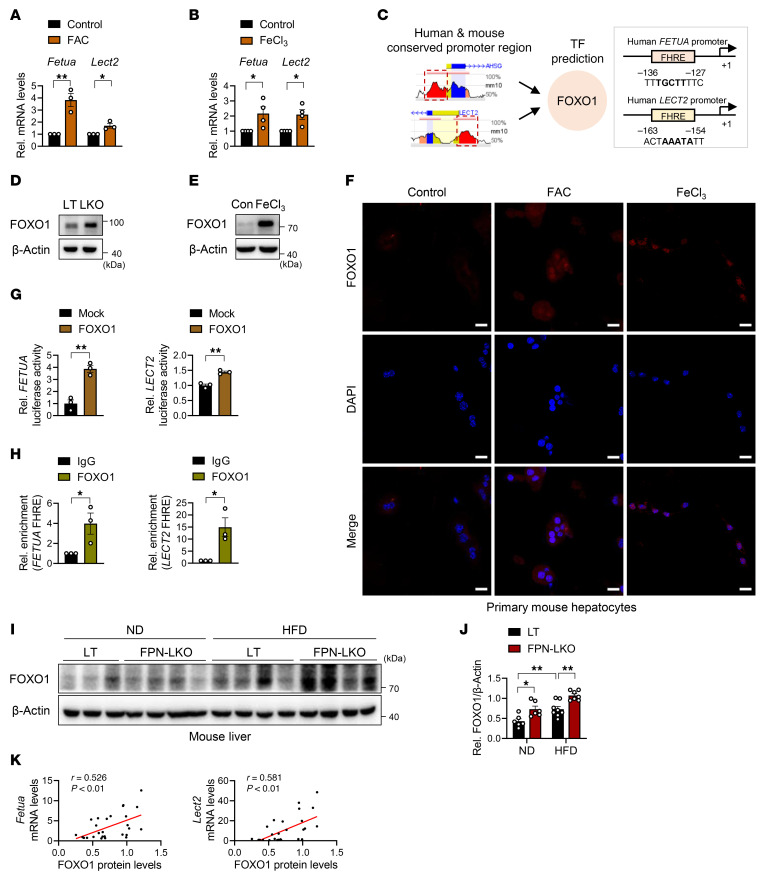
Hepatic iron overload induces fetuin-A and LECT2 expression via FoxO1 activation. (**A** and **B**) qPCR analyses showing increased *Fetua* and *Lect2* mRNA levels induced by the iron treatments FAC (100 μM, *n* = 3 per group) or FeCl_3_ (100 μM, *n* = 4 per group). (**C**) Identification of conserved FoxO1-binding motifs (forkhead response element [FHRE]) within *FETUA* and *LECT2* promoters. TF, transcription factor. (**D** and **E**) Western blot analysis of FoxO1 in primary hepatocytes from LT and *Fpn*-LKO mice (**D**) or in primary mouse hepatocytes treated with FeCl_3_ (**E**). (**F**) IF staining demonstrating FoxO1 nuclear translocation in primary mouse hepatocytes treated with FAC or FeCl_3_. Scale bars: 20 μm. (**G**) Luciferase reporter assays confirming FoxO1-mediated transcriptional activation of *FETUA* and *LECT2* in HepG2 cells overexpressing FoxO1 (*n* = 3 per group). (**H**) ChIP assays validating direct FoxO1 binding to promoter regions of target genes (*n* = 3 per group). (**I** and **J**) Western blot analysis of FoxO1 and its quantification in the livers of ND- or HFD-fed LT and *Fpn*-LKO mice (*n* = 6–8 per group). (**K**) Positive correlations between hepatic FoxO1 expression and transcript levels of *Fetua* and *Lect2*. Pearson correlation coefficients (*r*) and corresponding *P* values are shown. Data are presented as the mean ± SEM. *P < 0.05 and **P < 0.01, by unpaired, 2-tailed Student’s *t* test.

To determine whether iron accumulation directly drives fetuin-A and LECT2 expression, we treated primary mouse hepatocytes with ferric ammonium citrate (FAC) or ferric chloride (FeCl_3_). Both treatments significantly upregulated *Fetua* and *Lect2* mRNA levels (Figure 6, A and B). Since transcript levels were altered, we analyzed the promoter regions of these genes using the Evolutionary Conserved Regions (ECR) browser to identify potential transcriptional regulators. FoxO1 binding motifs were identified within the proximal promoter regions of *FETUA* and *LECT2*, with these elements being conserved across human and murine species (Figure 6C). FPN deficiency in hepatocytes increased FoxO1 expression (Figure 6D), an effect replicated by direct iron treatment (Figure 6E). Immunofluorescence (IF) staining demonstrated FoxO1 nuclear translocation and activation in iron-treated hepatocytes (Figure 6F), as well as in hepatocytes from HFD-fed mice (Supplemental Figure 11C). Luciferase reporter assays confirmed that FoxO1 overexpression significantly enhanced *FETUA* and *LECT2* transcriptional activity (Figure 6G), while ChIP assays validated direct FoxO1 binding to the respective promoter regions of these genes (Figure 6H). Consistent with these findings, HFD feeding increased hepatic FoxO1 protein levels, which were further elevated by hepatocyte-specific FPN deficiency (Figure 6, I and J). Moreover, hepatic FoxO1 expression positively correlated with *Fetua* and *Lect2* transcript levels (Figure 6K), supporting a functional link between iron-induced FoxO1 activation and hepatokine expression in vivo.

We further examined the effects of known regulatory pathways for these hepatokines. Notably, FPN deficiency in hepatocytes did not affect NF-κB or ERK signaling (previously implicated in fetuin-A regulation) (30, 31) or AMPK signaling (associated with LECT2 regulation) (32) (Supplemental Figure 11D). These findings demonstrate that iron accumulation resulting from FPN deficiency promoted the expression of the hepatokines fetuin-A and LECT2 through FoxO1-mediated transcriptional activation, establishing a molecular link between hepatic iron accumulation and systemic metabolic dysfunction.

To investigate the molecular mechanisms underlying iron-mediated FoxO1 induction and subsequent hepatokine regulation, we examined the potential involvement of oxidative stress, given the established role of iron in ROS generation. Iron treatment increased FoxO1 expression in primary hepatocytes, and this effect was significantly attenuated by the iron chelator deferoxamine (DFO) (Supplemental Figure 11, E and F), supporting a direct role of iron. Moreover, iron-induced FoxO1 expression was markedly suppressed by the ROS scavenger *N*-acetylcysteine (NAC) (Supplemental Figure 11, E and F), indicating the contribution of oxidative stress. Considering the close link between ROS and JNK signaling (33), we further evaluated this pathway and found that treatment with the JNK inhibitor (JNKi) (SP600125) significantly reduced iron-induced FoxO1 activation (Supplemental Figure 11, E and F). Consequently, the inhibitory effects of DFO, NAC, and JNKi were also observed on iron-induced *Fetua* and *Lect2* expression (Supplemental Figure 11G). These results suggest that hepatic iron overload induced the expression of FoxO1 and these hepatokines, at least in part, through ROS-dependent JNK signaling.

### Hepatocyte iron overload drives metabolic dysfunction via fetuin-A and LECT2.

**Figure 7:**
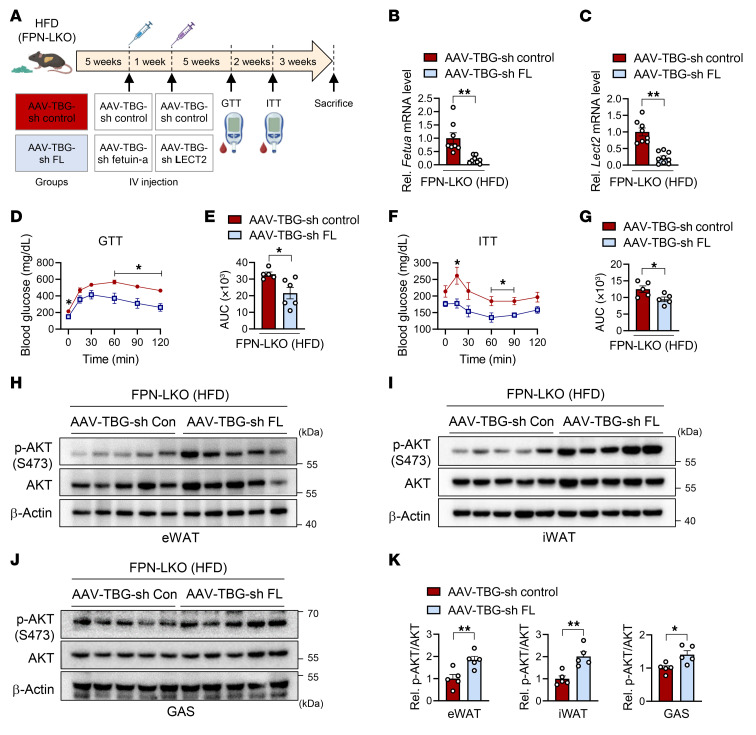
Silencing of fetuin-A and LECT2 ameliorates systemic metabolic dysfunction. (**A**) Experimental design illustrating hepatocyte-specific silencing of fetuin-A or LECT2 via AAV-mediated shRNA delivery under the control of the *TBG* promoter during HFD feeding of *Fpn*-LKO mice. (**B** and **C**) qPCR analysis validating effective knockdown of *Fetua* and *Lect2* mRNA levels (*n* = 8–9 per group). (**D** and **E**) GTT analysis (*n* = 5–6 per group). (**F** and **G**) ITT analysis (*n* = 5 per group). (**H**–**K**) Western blot analysis of p-Akt (S473) status across peripheral tissues including eWAT, iWAT, and GAS muscle tissues following knockdown intervention, with quantification of band intensities (*n* = 5 per group). Data are presented as the mean ± SEM. *P < 0.05 and **P < 0.01, by unpaired, 2-tailed Student’s *t* test.

Having established that fetuin-A and LECT2 expression was elevated in FPN-deficient hepatocytes, we next investigated whether targeted silencing of these hepatokines could ameliorate metabolic dysfunction in *Fpn*-LKO mice. We used a hepatocyte-specific genetic knockdown strategy using an adeno-associated virus (AAV) delivery system carrying shRNAs targeting fetuin-A and LECT2 under the control of the thyroxine-binding globulin (TBG) promoter. These viral vectors were administered to *Fpn*-LKO mice during the course of HFD feeding (Figure 7A). Efficient knockdown of fetuin-A and LECT2 was confirmed in liver tissues (Figure 7, B and C). Body weight gain remained largely unaffected under these experimental conditions (Supplemental Figure 12A).

Notably, GTT and ITT demonstrated that inhibition of both fetuin-A and LECT2 expression significantly improved hyperglycemia and IR in HFD-fed *Fpn*-LKO mice (Figure 7, D–G). At the molecular level, p-Akt levels were markedly elevated in eWAT, iWAT, and GAS muscle of hepatokines-silenced *Fpn*-LKO mice (AAV-TBG-sh FL) compared with control *Fpn*-LKO mice (AAV-TBG-sh Con) (Figure 7, H–K), indicating substantially restored insulin sensitivity in peripheral metabolic tissues.

**Figure 8:**
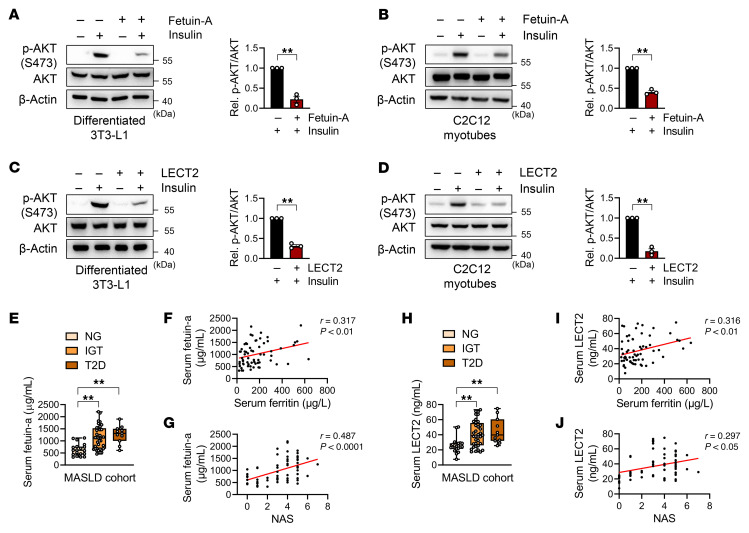
Exogenous fetuin-A and LECT2 impair insulin sensitivity in adipocytes and myotubes. (**A**–**D**) Inhibitory effects of exogenous fetuin-A (**A** and **B**) and LECT2 (**C** and **D**) on insulin signaling in differentiated 3T3-L1 adipocytes and C2C12 myotubes (*n* = 3 per group). (**E**–**J**) Serum levels of fetuin-A (**E**) and LECT2 (**H**), along with their positive correlations with serum ferritin (**F** and **I**) and NAS (**G** and **J**) levels. Box-and-whisker plots (median, IQR, minimum–maximum) with all individual data points are shown. Pearson correlation coefficients (*r*) and corresponding *P* values are indicated. Data are presented as the mean ± SEM. **P < 0.01, by unpaired, 2-tailed Student’s *t* test (**A**–**D**) or 1-way ANOVA followed by Tukey’s multiple-comparison test (**E** and **H**).

Next, we examined whether exogenous fetuin-A and LECT2 directly impair insulin signaling. Treatment with fetuin-A significantly inhibited insulin-stimulated Akt phosphorylation in differentiated 3T3-L1 adipocytes and C2C12 myotubes (Figure 8, A and B). We observed similar inhibitory effects following recombinant LECT2 treatment (Figure 8, C and D), indicating the causal roles of these hepatokines in promoting peripheral IR in adipose tissue and skeletal muscle.

To determine whether either hepatokine plays a predominant role, we performed GTT and ITT analyses following individual knockdown of fetuin-A or LECT2. Silencing of each hepatokine alone resulted in metabolic improvements comparable to those observed with combined knockdown (Supplemental Figure 12, B–F), indicating no clear additive or synergistic effects under our experimental conditions. We further assessed the individual and combined effects of exogenous hepatokines on peripheral insulin signaling and found that cotreatment with fetuin-A and LECT2 enhanced the inhibitory effect in adipocytes (Supplemental Figure 12, G and H), whereas in myotubes, the effects of single and combined treatments were comparable (Supplemental Figure 12, I and J). These findings suggest tissue-specific interactions between fetuin-A and LECT2, providing a potential explanation for the comparable metabolic improvements observed in vivo after single versus combined knockdown.

Moreover, we assessed their serum levels in human clinical samples and found that fetuin-A and LECT2 concentrations were significantly higher in individuals with impaired glucose tolerance or T2D than in those with normoglycemia within the MASLD cohort (Figure 8, E and H). Serum levels of these hepatokines were positively correlated with both serum ferritin and NAS levels (Figure 8, F, G, I, and J). These findings further support the clinical relevance of fetuin-A and LECT2 in MASLD-associated metabolic dysfunction and suggest that elevated hepatokine levels are linked to worsening glycemic control and disease severity.

**Figure 9:**
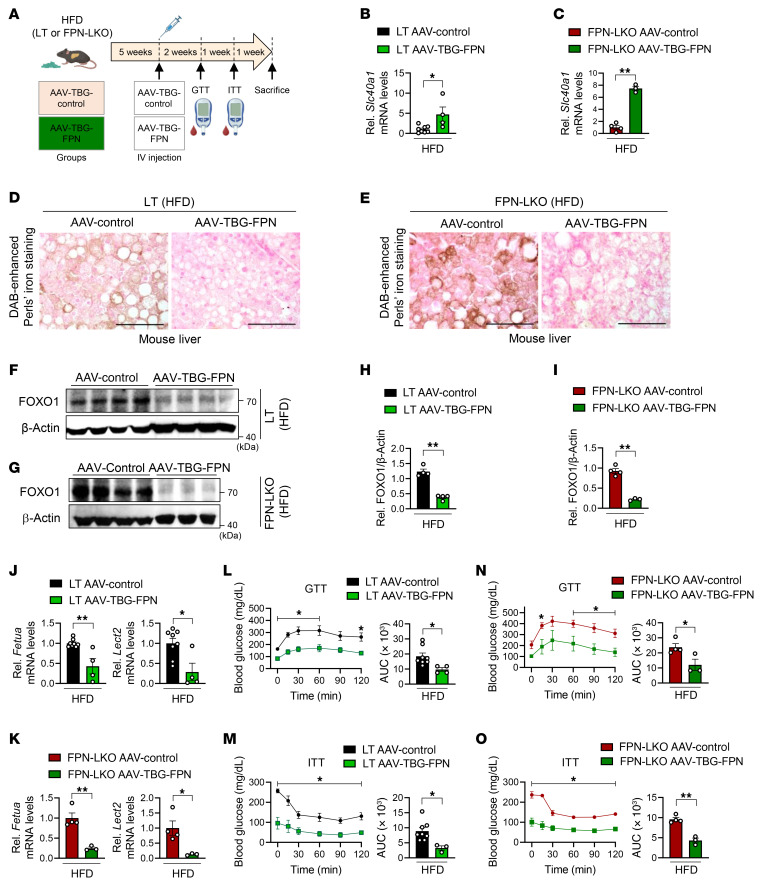
Hepatocyte-specific FPN overexpression improves systemic metabolic dysfunction. (**A**) Experimental design illustrating hepatocyte-specific overexpression of FPN via AAV-mediated delivery under the control of the *TBG* promoter during HFD feeding in LT and *Fpn*-LKO mice. (**B** and **C**) qPCR analysis confirming hepatic FPN overexpression in LT mice (**B**) (*n* = 4–8 per group) and *Fpn*-LKO mice (**C**) (*n* = 3–4 per group). (**D** and **E**) DAB-enhanced Perls’ staining demonstrating reduced hepatic iron levels following FPN overexpression. Scale bars: 50 μm. (**F**–**I**) Western blot analysis showing decreased FoxO1 expression following FPN overexpression in LT mice (**F** and **H**) (*n* = 4 per group) and *Fpn*-LKO mice (**G** and **I**) (*n* = 3–4 per group). (**J** and **K**) qPCR analysis demonstrating downregulation of *Fetua* and *Lect2* following FPN overexpression in LT mice (**J**) (*n* = 4–8 per group) and *Fpn*-LKO mice (**K**) (*n* = 3–4 per group). (**L** and **M**) GTT and ITT analyses in LT mice (*n* = 3–8 per group). (**N** and **O**) GTT and ITT analyses in *Fpn*-LKO mice (*n* = 3–4 per group). Data are presented as the mean ± SEM. *P < 0.05 and **P < 0.01, by unpaired, 2-tailed Student’s *t* test.

To further investigate the role of hepatic iron in hepatokine-driven metabolic dysfunction, we applied a hepatocyte-specific FPN overexpression strategy using AAV-TBG-FPN to directly enhance hepatic iron export (Figure 9A). Efficient FPN overexpression was confirmed in the livers of HFD-fed LT mice (Figure 9B). Forced FPN expression reduced hepatic iron deposition (Figure 9D), decreased FoxO1 expression (Figure 9, F and H), suppressed downstream *Fetua* and *Lect2* expression (Figure 9J), and significantly improved glucose tolerance and insulin sensitivity (Figure 9, L and M), supporting the in vivo relevance of the iron/FoxO1/hepatokine axis in metabolic dysfunction. We observed similar alterations in this signaling axis and metabolic improvements following FPN reexpression in *Fpn*-LKO mice (Figure 9, C, E, G, I, K, N, and O), confirming a hepatocyte-autonomous role of FPN.

**Figure 10:**
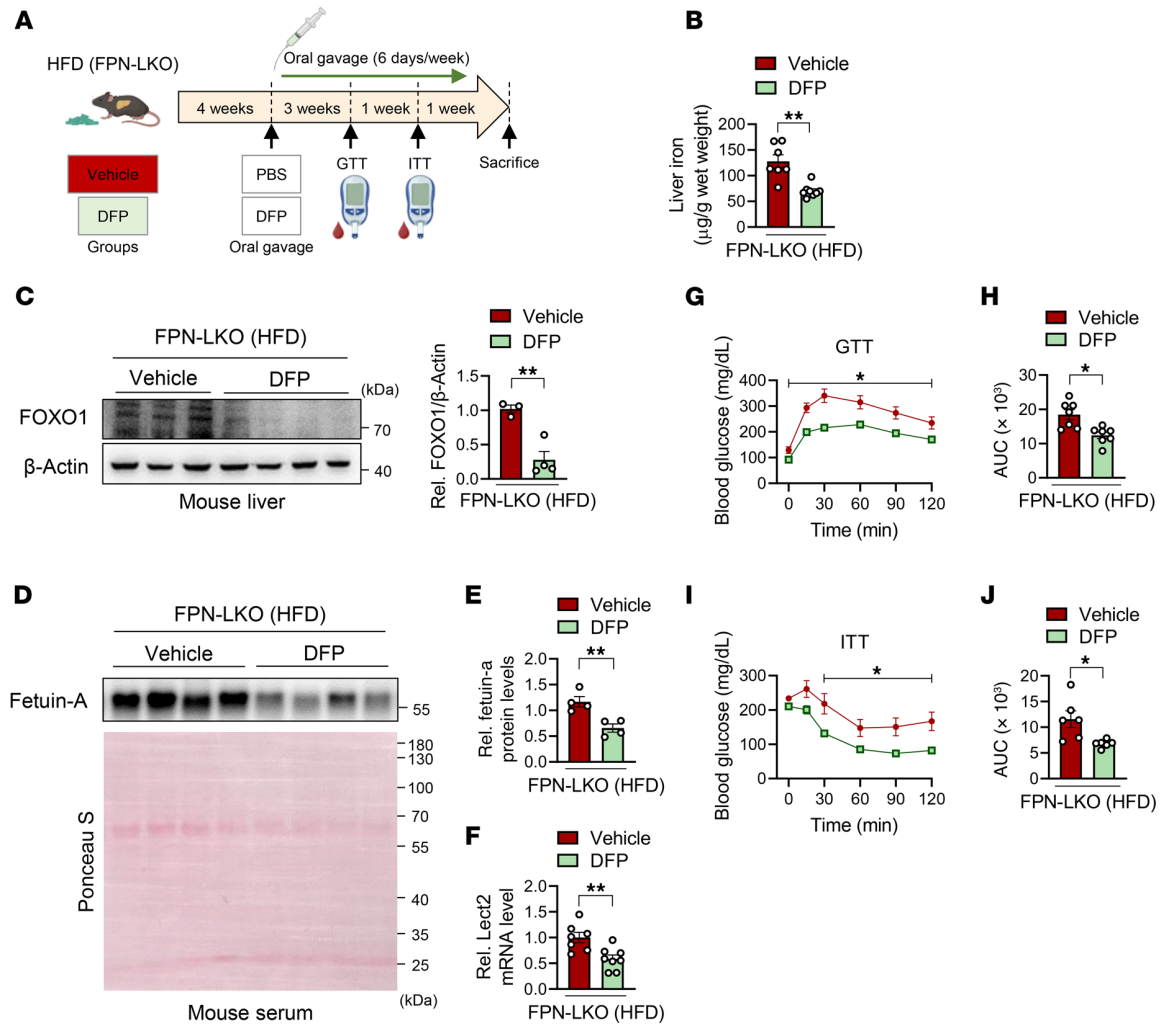
Oral iron chelation ameliorates systemic metabolic dysfunction. (**A**) Therapeutic administration protocol of an oral iron chelator, DFP, in HFD-fed *Fpn*-LKO mice. (**B**) Quantification of hepatic iron content (*n* = 7–8 per group). (**C**) Western blot analysis of hepatic FoxO1 expression (*n* = 3–4 per group). (**D** and **E**) Western blot analysis of serum fetuin-A and corresponding quantification (*n* = 4 per group). (**F**) qPCR analysis of hepatic *Lect2* mRNA levels (*n* = 7–8 per group). (**G** and **H**) GTT analysis (*n* = 7 per group). (**I** and **J**) ITT analysis (*n* = 6 per group). Data are presented as the mean ± SEM. *P < 0.05 and **P < 0.01, by unpaired, 2-tailed Student’s *t* test.

We next evaluated the therapeutic potential of pharmacological iron chelation by administering deferiprone (DFP), an FDA-approved, orally bioavailable iron chelator, to *Fpn*-LKO mice. After 4 weeks of HFD feeding, *Fpn*-LKO mice were treated with either vehicle or DFP (administered 6 days per week for 5 weeks), while continuing HFD feeding (Figure 10A). Mouse body weights remained comparable between treatment groups (Supplemental Figure 13A). As expected, DFP treatment significantly reduced hepatic iron accumulation (Figure 10B). This reduction in hepatic iron levels was accompanied by a corresponding decrease in the expression of FoxO1 (Figure 10C) and the hepatokines fetuin-A and LECT2 (Figure 10, D–F). Consistent with these molecular changes, glucose tolerance and insulin sensitivity were significantly improved in DFP-treated mice, as assessed by GTTs and ITTs (Figure 10, G–J). Moreover, CM derived from hepatocytes isolated from DFP-treated *Fpn*-LKO mice markedly restored insulin-stimulated Akt phosphorylation in both adipocytes and myotubes compared with CM from vehicle-treated *Fpn*-LKO hepatocytes (Supplemental Figure 13, B–D), further supporting a humoral regulatory role of hepatic iron in modulating systemic insulin sensitivity.

Given the reported effects of iron chelation on mitophagy and the importance of mitochondrial quality control in MASLD pathogenesis (34, 35), we further examined the expression of genes related to mitophagy, mitochondrial function, and mitochondrial biogenesis. However, DFP treatment did not significantly alter the expression of mitophagy-associated genes and instead reduced the transcript levels of several genes involved in mitochondrial biogenesis (Supplemental Figure 13E). These findings suggest that, under our experimental conditions, DFP did not markedly affect mitochondrial quality control or dynamics.

To further validate the effect of iron overload, we used an in vivo iron dextran-loading model (Supplemental Figure 14A). Iron dextran administration impaired glucose tolerance under both ND and HFD conditions (Supplemental Figure 14, B–E), indicating a beneficial effect of iron chelation in ameliorating metabolic dysfunction.

Collectively, these findings provide compelling evidence that attenuating hepatic iron overload mitigates metabolic dysfunction by suppressing the iron/FoxO1/hepatokine axis, highlighting the therapeutic potential of targeting iron homeostasis and hepatokine regulation in MASLD-associated metabolic disorders.

## Discussion

MASLD is increasingly recognized as both a consequence and driver of systemic metabolic diseases, yet the precise molecular mechanisms underlying this bidirectional relationship remain incompletely understood. Our study provides compelling evidence that hepatic iron overload, driven by FPN downregulation in hepatocytes, serves as a critical pathogenic factor linking MASLD to systemic metabolic dysfunction. Specifically, our findings across both clinical cohorts and experimental models demonstrate that hepatic FPN downregulation in MASLD led to iron accumulation, which subsequently and profoundly altered hepatokine secretion profiles, particularly of fetuin-A and LECT2 via FoxO1 activation, ultimately promoting obesity, IR, and impaired thermogenesis. Our results establish a mechanistic framework that links hepatic iron homeostasis disruption to systemic metabolic consequences, helping to address a significant knowledge gap in MASLD pathophysiology and offering perspectives on metabolic disease development.

Clinical studies have consistently shown strong associations between elevated serum ferritin levels and metabolic syndrome components across diverse populations (14, 18, 36, 37), but mechanistic understanding has remained limited. Our clinical analyses extend these findings by demonstrating that serum ferritin independently predicted IR in patients with MASLD, even after rigorous adjustment for confounding factors such as age, BMI, lipid profiles, and hepatic pathology. Importantly, this association strengthens with increasing steatosis severity, highlighting the utility of ferritin as both a diagnostic and prognostic biomarker in MASLD management.

Following our identification of reduced FPN expression in both human and murine MASLD, we generated and characterized *Fpn*-LKO mice. Consistent with clinical observations, these mice exhibit a constellation of metabolic abnormalities, including exacerbated obesity, glucose intolerance, and IR, when challenged with a HFD. Notably, these metabolic perturbations were accompanied by significantly reduced energy expenditure, impaired thermogenic capacity in adipose tissues, and decreased exercise performance — hallmarks of metabolic dysfunction that extend beyond the liver. These findings align with previous studies demonstrating the critical role of energy expenditure and thermogenesis in obesity-related metabolic disorders. Remarkably, unlike adipocyte-specific *Fpn*-KO mice that do not exhibit significant metabolic disturbances upon high-calorie feeding (38), our hepatocyte-specific model clearly demonstrated cell-type– and tissue-specific roles of FPN and iron metabolism in systemic metabolic regulation. This tissue specificity underscores the central role of hepatocyte iron homeostasis in orchestrating whole-body metabolic responses. These conclusions are further supported by the metabolic improvements achieved through hepatocyte-specific FPN overexpression.

The obesity and diabetic phenotypes were manifested in both male and female *Fpn*-LKO mice, indicating sex-independent effects of hepatic iron dysregulation. This observation aligns with our clinical findings showing strong correlations between serum ferritin levels and metabolic parameters across both sexes, suggesting broad applicability of the iron-metabolism connection. Iron has long been recognized as a crucial micronutrient with numerous physiological functions, but emerging evidence points to tissue-specific roles for iron-regulatory proteins that extend beyond traditional iron handling. Our work significantly expands this understanding by demonstrating that hepatocyte-specific disruption of iron export machinery can profoundly influence systemic energy homeostasis and insulin sensitivity without necessarily altering systemic iron parameters.

To understand the mechanisms underlying the metabolic phenotypes observed in *Fpn*-LKO mice, we conducted detailed analyses of iron content across multiple metabolic tissues. As expected, FPN deficiency led to significant iron accumulation specifically within the liver. Intriguingly, despite this hepatic iron overload, serum iron levels remained unchanged between *Fpn*-LKO and control mice, as did iron content in peripheral metabolic tissues including adipose depots and skeletal muscle. This compartmentalized pattern of iron accumulation aligns with previous research demonstrating that hepatic FPN deficiency primarily affects iron mobilization from liver stores and has a minimal effect on systemic iron parameters under iron-sufficient dietary conditions (39). The observation that peripheral tissue iron content remained unaltered in our model is particularly significant, as it indicates that the metabolic effects of hepatocyte FPN deficiency were not mediated by direct iron-induced changes in adipose tissue and muscle metabolism. Instead, our findings point to an alternative mechanism: altered liver-derived humoral signals that influence peripheral tissue insulin sensitivity.

The concept of cell-type–specific iron compartmentalization has gained increasing attention in metabolic disease research. Recent studies have demonstrated that adipocyte iron content can influence lipid handling through fat-gut crosstalk (22), while macrophage iron loading in adipose tissue affects inflammatory responses and metabolic deterioration (40). Our work extends this paradigm by establishing that hepatocyte-specific iron accumulation has distinct and far-reaching metabolic consequences through the modulation of hepatokine secretion.

A pivotal finding of our study is the identification of fetuin-A and LECT2 as critical hepatokines mediating the systemic metabolic consequences of hepatic iron overload. Through the unbiased RNA-seq analysis of liver samples from patients with MASLD, followed by systematic refinement based on the correlation with iron storage markers, secretion potential, and metabolic relevance, we identified these 2 hepatokines as key mediators linking hepatic iron accumulation to peripheral IR.

Fetuin-A has been established as a clinically relevant hepatokine whose circulating levels correlate strongly with the risk of IR and T2D (7, 41–44). Mechanistically, fetuin-A promotes IR by acting as an adaptor protein that facilitates free fatty acid–induced activation of TLR4 signaling in adipocytes (45, 46) and by suppressing glucose uptake in myocytes (47). Similarly, LECT2 has recently emerged as an important hepatokine associated with obesity-related IR (32, 48). Functionally, LECT2 has been shown to impair insulin signaling in both myocytes (32) and adipocytes (49), while also acting as a ligand of Tie1 to promote sinusoidal capillarization and liver fibrogenesis (50). Previous studies have demonstrated beneficial effects on insulin sensitivity following genetic deletion of these hepatokines in mice (32, 51, 52). In line with these reports, our data demonstrate that exogenous fetuin-A and LECT2 directly suppressed insulin-stimulated Akt phosphorylation in both adipocytes and myotubes, indicating their causal roles in peripheral IR. Interestingly, cotreatment with both hepatokines exerted additive inhibitory effects on adipocytes but not on myotubes, suggesting potential tissue-specific differences in signaling integration or pathway saturation. The mechanistic basis for this divergence warrants further investigation. Circulating fetuin-A and LECT2 levels were elevated in metabolically impaired individuals with MASLD and positively associated with ferritin and disease severity, reinforcing their clinical relevance. Notably, our findings reveal what we believe to be a previously unrecognized regulation of fetuin-A and LECT2 by iron status, adding an important dimension to our understanding of their roles in metabolic disease.

Our hepatocyte-specific knockdown experiments, coupled with iron chelation therapy, provide compelling evidence for the causal roles of these hepatokines in iron-induced metabolic dysfunction. Notably, unlike constitutive KO models, our acute hepatocyte-specific silencing of fetuin-A and LECT2 in *Fpn*-LKO mice reversed IR without significantly altering body weight gain, suggesting context-dependent metabolic effects that may relate to the timing, magnitude, or tissue specificity of intervention. These results highlight the direct contribution of these hepatokines to IR pathogenesis rather than merely representing a secondary consequences of obesity.

Another key contribution of our study is the identification of a regulatory mechanism linking iron status to hepatokine production. Recent studies have begun to elucidate the complex interplay between hepatokines and various metabolic stressors, including lipotoxicity. While previous studies have suggested that palmitate (30) or high glucose (31) can induce fetuin-A expression via NF-κB or ERK pathways in hepatocytes, we discovered that iron overload, either through FPN deficiency or direct iron treatment, potently stimulated the expression of both fetuin-A and LECT2. Conversely, hepatocyte-specific FPN overexpression or pharmacological iron chelation effectively suppressed their production, establishing these hepatokines as bona fide iron-responsive factors. Therefore, our work adds iron overload as a significant regulator of the hepatokine secretory profile, expanding our understanding of the diverse environmental factors that can influence liver-derived humoral signals.

Through careful promoter analysis and functional validation, we identified FoxO1 as the transcriptional mediator connecting iron accumulation to enhanced hepatokine expression. FoxO1 is widely recognized as a master regulator of glucose and lipid metabolism, playing crucial roles in hepatic gluconeogenesis, lipogenesis, and insulin signaling (53). We found that iron treatment substantially upregulated FoxO1 expression and nuclear translocation in hepatocytes, whereas iron chelation markedly attenuated this induction. These findings are consistent with previous reports of iron-induced FoxO1 activation in other cell types, including chondrocytes (54) and cardiomyocytes (55), suggesting a broader role for iron/FoxO1 signaling across diverse tissues. Importantly, FoxO1 expression was elevated in FPN-deficient livers and reduced following hepatocyte-specific FPN overexpression, further supporting a causal link between hepatic iron status and FoxO1 activation in vivo.

The FoxO family of transcription factors serve as important integrators of metabolic and stress signals, responding to insulin signaling, oxidative stress, and nutrient availability (53, 56). Recent studies have implicated FoxO1 in various aspects of iron-related cellular responses, including ferroptosis sensitivity and adaptation to iron overload (57, 58). Our finding that FoxO1 directly bound to and activated the promoters of fetuin-A and LECT2 in response to iron establishes a connection between iron metabolism and hepatokine regulation. Notably, iron-induced FoxO1 activation and subsequent hepatokine expression were attenuated by ROS scavenging and JNK inhibition, indicating a ROS-dependent JNK signaling mechanism upstream of FoxO1. Similar ROS/JNK/FoxO1 signaling has been reported in other cell types (59, 60), highlighting the broader relevance of this stress-responsive axis. It is noteworthy that our findings did not show alterations in previously established regulatory pathways for fetuin-A and LECT2, such as NF-κB, ERK, or AMPK signaling, further underscoring the specificity of the iron/FoxO1 axis in their regulation. Recent work has highlighted the therapeutic potential of targeting FoxO transcription factors in metabolic diseases (53). Our findings suggest that modulating the iron/FoxO1/hepatokine axis could represent an approach to addressing metabolic dysfunction in MASLD, potentially offering greater specificity than global FoxO1 inhibition.

Emerging clinical evidence aligns with our findings, as metabolic hyperferritinemia has been associated with increased risks of T2D, cardiovascular complications, and adverse long-term outcomes for patients with MASLD (61–63), reinforcing its potential utility in clinical risk stratification. Elevated serum ferritin in advanced MASLD likely reflects a complex interplay between iron burden and inflammatory status, underscoring the need for cautious interpretation when considered alone. Within this framework, the direct histologic demonstration of hepatic iron accumulation in our study strengthens the inference that iron overload contributes to hepatokine-driven metabolic dysfunction.

The strong correlation between serum ferritin and IR parameters in our MASLD cohort, coupled with the metabolic improvements observed following iron chelation in our mouse model, suggests that stratifying patients on the basis of iron status could help identify those most likely to benefit from iron-targeted interventions. Recent consensus statements on the definition and classification of metabolic hyperferritinemia provide a framework for such stratification, potentially enabling targeted therapeutic interventions aimed at correcting hepatic iron dysregulation before significant metabolic dysfunction occurs.

Our findings provide compelling evidence that addressing hepatic iron overload could represent an effective therapeutic strategy for managing systemic metabolic disorders associated with MASLD. Clinical studies have previously suggested beneficial effects of systemic iron reduction via phlebotomy or chelation on diabetic phenotypes, though the mechanisms remained unclear (64). Our work reveals altered hepatokine secretion to be a key mediator of these effects, providing a mechanistic rationale for iron-targeted interventions.

However, whole-body iron depletion strategies carry risks such as potential iron deficiency anemia, highlighting the importance of targeted iron modulation strategies. Thus, our demonstration that hepatocyte-specific iron accumulation drove metabolic dysfunction suggests that liver-directed iron chelation could provide metabolic benefits while minimizing systemic side effects. The efficacy of the orally administered iron chelator DFP in our mouse model supports the clinical potential of this approach. Selective iron reduction achieved by hepatocyte-specific FPN overexpression, as well as iron chelation with DFP, effectively reverses IR, emphasizing the therapeutic potential of targeted liver-directed interventions. Recent developments in iron chelation technology are particularly encouraging, with a chelator designed specifically for MASH showing promise in preclinical models (26). The FDA-approved status of several iron chelators, including DFP used in our study, could facilitate the rapid clinical translation of our findings. Experimental approaches to achieve tissue-specific iron modulation, such as targeted nanoformulations or conjugated chelators, represent an exciting frontier that could further enhance the therapeutic index of iron-directed interventions.

Beyond iron chelation, our findings suggest that targeting specific hepatokines could represent an alternative approach to addressing metabolic dysfunction in MASLD. While fibroblast growth factor 21 has been extensively studied as a potential therapeutic target (7), with several analogs in clinical development (65), our work identifies fetuin-A and LECT2 as additional candidates. The observation that silencing these hepatokines improved insulin sensitivity in our model, coupled with their clinical correlations with metabolic dysfunction, supports their potential as therapeutic targets. Interestingly, certain existing interventions may already act in part through modulation of these hepatokines. Lifestyle modifications promoting weight loss (41, 66) and thiazolidinedione therapy (67) have been associated with reduced circulating fetuin-A levels, potentially contributing to the metabolic benefits associated with these interventions. Our finding that iron chelation suppressed fetuin-A and LECT2 production may provide a pharmacological approach to modulating these hepatokines.

Several important questions emerge from our findings and warrant further investigation. The upstream mechanisms leading to FPN downregulation in MASLD remain to be fully elucidated and may involve transcriptional, posttranscriptional, and/or posttranslational regulation. Understanding these pathways could identify additional therapeutic targets to prevent hepatic iron accumulation. Although our data support a role for ROS-dependent JNK signaling in iron-mediated FoxO1 activation, additional upstream pathways may also be involved and remain to be elucidated. Further analyses could identify other hepatokines or extracellular vesicles potentially contributing to systemic metabolic dysfunction.

The heterogeneity of MASLD and its associated metabolic comorbidities underscore the need for precision medicine approaches tailored to specific pathophysiological drivers. Our identification of the iron/FoxO1/hepatokine axis represents an important addition to the evolving understanding of MASLD pathomechanisms and will potentially enable more targeted therapeutic strategies for patients with evidence of iron dysregulation. The recent reclassification of MASLD into distinct endotypes based on molecular profiles (8) raises the possibility that iron-driven metabolic dysfunction represents a specific disease subtype amenable to tailored interventions. Future clinical studies incorporating iron parameters into patient stratification could help validate this concept and optimize therapeutic approaches.

In conclusion, our study identifies hepatic iron overload due to FPN downregulation as a pathogenic mechanism driving systemic metabolic dysfunction through altered hepatokine secretion mediated by FoxO1 activation. These findings establish what we believe to be a previously unexplored pathophysiological link between MASLD and its metabolic comorbidities, while identifying hepatic iron homeostasis as a promising therapeutic target. Modulation of liver iron content and/or its downstream hepatokine effects may represent a promising strategy for addressing the growing clinical challenge of MASLD-associated cardiometabolic complications.

## Methods

### Sex as a biological variable.

Our study included both male and female animals, with comparable results observed across sexes. Similarly, sex-stratified human clinical analyses yielded consistent findings.

Additional details can be found in Supplemental Methods.

### Human study.

We established a prospective cohort as part of the ongoing Boramae MASLD registry (NCT02206841) (68). The study included 656 Korean individuals who visited Seoul Metropolitan Government Boramae Medical Center, and their baseline characteristics are described in Supplemental Table 1. All participants underwent liver biopsy for histological assessment and serum collection. For subgroup analyses, the participants were categorized according to histologic NAS, IR status, and fibrosis stage as follows: normal (NAS = 0), MASL–no IR (NAS 1–2 and HOMA-IR <2.0), MASL-IR (NAS 1–2 and HOMA-IR ≥2.5), eMASH (NAS 3–4 and F0–1), and aMASH (NAS ≥5 and F ≥2).

### Mouse models.

WT C57BL/6N and C57BL/6J male mice were obtained from Samtako Bio Korea and The Jackson Laboratory, respectively. FPN-floxed (*Slc40a1*-floxed) mice (129S-*Slc40a1^tm2Nca^*/J, strain no. 017790) and *albumin-Cre* mice (B6.Cg-*Speer6-ps1^Tg(Alb-cre)21Mgn^*/J, strain no. 003574) were purchased from The Jackson Laboratory. Hepatocyte-specific *Fpn*-KO (*Fpn*-LKO) mice were generated by crossing *Fpn^fl/fl^* and *Alb-Cre* mice, followed by backcrossing with C57BL/6J mice for 10 generations. *Fpn^fl/fl^* mice served as LT controls. Genotypes were confirmed via PCR using specific primers (Supplemental Table 4). Mice were housed under controlled barrier conditions at 22°C ± 2°C, 50% ± 5% humidity, and a 12-hour light-dark cycle, with ad libitum access to food and water. Unless stated otherwise, they were fed a ND (Samtako Bio Korea). Mice were randomly assigned to groups according to sex and genotype. Eight-week-old male and female *Fpn*-LKO and LT mice were fed either a ND or a HFD with 60 kcal percentage fat (D12492, Research Diets,). Additionally, 6-week-old C57BL/6N male mice were fed either a ND, an l-amino acid diet with 60 kcal percentage of fat with 0.1% methionine and no added choline (CDAHFD; A06071302, Research Diets) for 12 weeks, or a diet containing 40 kcal percentage of fat (mostly palm oil), 20 kcal percentage of fructose, and 2% cholesterol (HFHFrHC) for 12 weeks.

### Cell culture.

Primary hepatocytes were isolated from male C57BL/6 WT, *Fpn^fl/fl^* LT control, and *Fpn*-LKO mice through a previously described procedure (69). Briefly, hepatocytes were isolated using collagenase type IV perfusion and 40% Percoll gradient centrifugation. Cells were seeded on collagen-coated plates and incubated in Medium 199 (M4530, MilliporeSigma). HepG2 (HB-8065), 3T3-L1 (CL-173), and C2C12 (CRL-1772) cell lines were sourced from American Type Culture Collection (ATCC) and maintained in DMEM with 10% FBS, 100 units/mL penicillin, and 100 μg/mL streptomycin. For adipocyte differentiation, confluent 3T3-L1 cells were exposed to differentiation media containing 0.5 mM 3-isobutyl-1-methylxanthine, 1 μM dexamethasone, and 10 μg/mL insulin (MDI cocktail) for 2 days, followed by culturing in DMEM with 10% FBS and 10 μg/mL insulin for another 2 days. The media were then replaced every 2 days for 4 days. For myotube differentiation, confluent C2C12 cells were maintained in media with 2% horse serum instead of 10% FBS for 6 days, with media changes every 2 days.

### IHC and IF staining.

For IHC, antigen retrieval was performed on deparaffinized sections using a citrate buffer solution (S1699, Dako, Agilent Technologies). The sections were then stained with specific primary antibodies against FPN (MTP11-A, Alpha Diagnostic International) or UCP1 (U6382, MilliporeSigma), followed by incubation with an HRP-conjugated horse anti–rabbit or anti–mouse IgG polymer detection kit (MP-7401 or MP-7402, Vector Laboratories). Visualization was carried out using a DAB substrate kit (SK-4100, Vector Laboratories).

For IF staining, antigen-retrieved tissue sections or cells were incubated with primary antibodies against FPN, F4/80 (ab6640, Abcam), and perilipin (70R-1297, Biosynth), followed by secondary antibodies: Alexa Fluor 488–conjugated goat anti–mouse IgG (A-11001, Invitrogen, Thermo Fisher Scientific) and DyLight 594-conjugated goat anti–rat IgG (A110-105D4, Bethyl Laboratories, Fortis Life Sciences). Sections were then counterstained with DAPI and visualized using a confocal laser-scanning microscope (Carl Zeiss).

For live-cell fluorescence imaging of intracellular iron, FerroOrange (36104, Cell Signaling Technology) was used in accordance with the manufacturer’s instructions. Following incubation with 1 μM FerroOrange in serum-free media for 30 minutes, the cells were examined using confocal microscopy.

### Perls’ Prussian blue iron staining.

Ferric iron in liver sections was detected using the Prussian blue–based Iron Stain kit (ab150674, Abcam). Sections were deparaffinized, incubated in freshly prepared potassium ferrocyanide and hydrochloric acid working solution, counterstained with Nuclear Fast Red, dehydrated, and then mounted. To enhance detection sensitivity, DAB-based signal amplification was subsequently performed as previously described (26, 70).

### Metabolic studies.

For the GTT, glucose (2 g/kg) was administered via oral gavage after mice had been fasted for 12 hours. For the ITT, insulin (0.75 U/kg for ND, and 1 U/kg for HFD) was injected i.p. following a 6-hour fast. Blood glucose levels were measured from the tail vein using a glucometer before glucose or insulin administration (baseline) and at the designated time points. For the metabolic cage studies, mice were individually housed in the Oxymax/CLAMS system (Columbus Instruments). After 24 hours of acclimation, metabolic parameters were continuously monitored for 72 hours under ad libitum feeding at 20°C–23°C with a 12-hour dark/12-hour light cycle (7 pm–7 am). The Oxymax system recorded energy expenditure, the respiratory exchange ratio (VO_2_/VCO_2_), food intake, and physical activity. Data from the last 48 hours of monitoring were used for analysis.

### Measurement of iron content.

Tissue iron levels were quantified using inductively coupled plasma–mass spectrometry (ICP-MS), with support from the Center for University-wide Research Facilities (CURF) at Jeonbuk National University. Briefly, weighed tissues were digested in nitric acid (HNO_3_) and diluted for analysis. Data are expressed as micrograms of iron per gram of wet tissue weight. TIBC and serum iron levels were measured using a commercial kit (ab239715, Abcam) following the manufacturer’s instructions. Transferrin saturation (percentage) was calculated on the basis of TIBC and serum iron values.

### AAV-based genetic modulation of hepatokines and FPN.

For hepatocyte-specific genetic knockdown of fetuin-A and LECT2, AAVs were generated by VectorBuilder. Each AAV8 carried 3 distinct shRNAs targeting mouse fetuin-A or LECT2 under the control of the *TBG* promoter. After 5 weeks of HFD feeding over a total 16-week feeding period, mice received a retro-orbital i.v. injection of a scrambled control AAV and AAV-TBG-sh fetuin-A, followed by a second injection of the control virus and AAV-TBG-sh LECT2 after a 1-week interval (5 × 10^11^ gc/mouse). For hepatocyte-specific FPN overexpression, mice were i.v. injected with AAV8-TBG-FPN or the corresponding control virus (5 × 10^11^ gc/mouse) after 5 weeks of HFD feeding during a total 9-week feeding period.

### Statistics.

The *n* values shown as dots in the figures indicate biological replicates. All results were obtained from at least 3 independent samples or experiments. Differences between 2 groups were evaluated using an unpaired, 2-tailed Student’s *t* test. For comparisons among multiple groups, 1-way ANOVA was conducted, followed by Tukey’s post hoc test for multiple comparisons. Statistical significance was defined as a *P*  value of less than 0.05. All statistical analyses were performed using GraphPad Prism 9.0 software (GraphPad Software). Data represent the mean ± SEM.

### Study approval.

This study was approved by the IRB of the Seoul Metropolitan Government Boramae Medical Center (no. 16-2013-45) and conducted in accordance with the principles of the Declaration of Helsinki. All participants were informed about the study protocol and provided written informed consent. Animal experiments were approved by the IACUC of Jeonbuk National University (no. NON2023-097) and Seoul National University (no. SNU-180410-6) and conducted in accordance with institutional guidelines.

### Data availability.

All data generated or analyzed during this study are available with this work. Raw and processed RNA-seq datasets generated in this study have been deposited in the Gene Expression Omnibus (GEO) database of the NCBI (GEO GSE292556). Values for all data points in graphs are reported in the Supporting Data Values file.

## Author contributions

HJJ, AK, HR, and CYH conceived the study and designed the experiments. HJJ, AK, HR, GHK, SJJ, HY, YJH, JMY, HCY, MYS, and JP conducted the experiments and analyzed the data. WK collected clinical samples. AKP, DHL, and WK analyzed the clinical cohort data. YHJ and WK performed bulk RNA-seq analysis, and SEH, HJY, MC and WK conducted snRNA-seq analysis. DYO, PES, BHP, and EJB provided technical support and valuable suggestions. CSC, SHK, KWK, BHP, EJB, and SGK contributed materials and additional technical support. HJJ, AK, HR, WK, and CYH wrote and revised the manuscript. WK and CYH supervised the study. The co–first authors are listed in order of academic seniority, from trainee to faculty member.

## Conflict of interest

The authors have declared that no conflict of interest exists.

## Funding support

- National Research Foundation of Korea (NRF) (2020R1C1C1003652, 2021R1I1A3041149, RS-2024-00344251, RS-2026-25476540, CYH; 2021R1A2C2005820, RS-2021-NR056442, RS-2022-NR067269, RS-2023-00223831, RS-2024-00440883, RS-2025-25458964, to WK; RS-2024-00357518, to HR; 2018R1A2A1A05078694, RS-2024-00441114, RS-2024-00454443, to SGK) funded by the Ministry of Science and ICT (MSIT).
- Bio & Medical Technology Development Program of the NRF (RS-2026-25517589, CYH; 2022M3E5F2017607, to EJB) funded by the MSIT.
- Basic Science Research Program through the NRF funded by the Ministry of Education (RS-2024-00409681, HJJ; RS-2025-25418088, to GHK).

## Supplementary Material

Supplemental data

Unedited blot and gel images

Supporting data values

## Figures and Tables

**Table 1:**
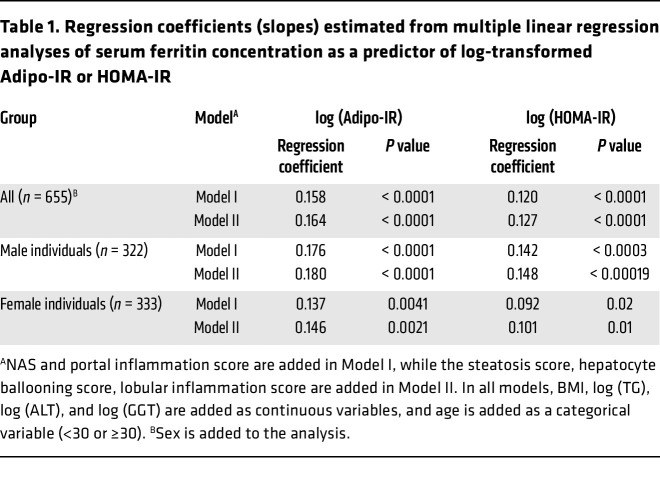
Regression coefficients (slopes) estimated from multiple linear regression analyses of serum ferritin concentration as a predictor of log-transformed Adipo-IR or HOMA-IR

## Version 1 04/28/2026

In-Press Preview

## Version 2 06/15/2026

Electronic publication
