# Supplementary material for: Iron overload in steatotic hepatocytes drives systemic metabolic dysfunction via alterations in hepatokine production
Source: J Clin Invest. 2026 Apr 28;136(12):e196374. doi: 10.1172/JCI196374 (PMC13262725; doi:10.1172/JCI196374)
Supplement: Supplemental data [file jci-136-196374-s284.pdf]

Supplemental material for

Iron overload in steatotic hepatocytes drives systemic metabolic dysfunction via alterations in hepatokine production

Additional Methods

Supplemental Figure 1–14 and Supplemental Table 1–5

Additional Methods

Histology, H&E and Oil Red O staining

Tissues were fixed in 10% neutral buffered formalin solution (#HT501128, Sigma-Aldrich, Merck KGaA, Darmstadt, Germany). After paraffin embedding, samples were sectioned at a thickness of 6 μm and mounted on slides. The sections were stained with hematoxylin and eosin (H&E) for morphological analysis using a light microscope (Olympus, Tokyo, Japan). Adipocyte area within the selected fat tissue sections was assessed using iSolution DT 36 software (Carl Zeiss, Oberkochen, Germany). Crown-like structures were quantified by counting them in five distinct high-power view fields per section. For Oil Red O staining, cryosections (10 μm thick) from tissues embedded in OCT compound and frozen using liquid nitrogen-cooled isopentane were incubated with the dye for 15 minute and then washed with phosphate-buffered saline (PBS).

Blood biochemical analyses

Serum ALT (#EALT-100, BioAssay Systems, Hayward, CA, USA) and AST (#EASTR-100, BioAssay Systems) activities were measured using commercially available assay kits according to the manufacturer's instructions.

Body temperature measurement

For cold exposure, thermoneutralized mice (30°C) were individually housed in a 6°C cold chamber. Rectal temperature was measured with a rectal thermometer at the indicated time, and surface body temperature was assessed via infrared thermography using a GTC-600 infrared camera at 48 hours. BAT and iWAT tissues were collected for UCP1 analysis.

Endurance exercise test

Mice were acclimated for two days before testing. The treadmill was set at a 5% incline with an initial speed of 10 m/min for 30 minutes. The speed was then increased by 2 m/min every 10 minutes until it reached 16 m/min, and the mice continued running until exhaustion. Running time and distance were measured for each mouse.

RNA isolation and qPCR assays

Total RNA was extracted using the RNA Iso Kit (#9109, Takara Bio Inc., Shiga, Japan) and reverse-transcribed with oligo-(dT)16 primers to generate complementary DNA (cDNA). Quantitative polymerase chain reaction (qPCR) was performed according to the manufacturer's instructions using a QuantStudio 5 Real-Time PCR System (Applied Biosystems, Thermo Fisher Scientific) and TOPreal SYBR Green qPCR PreMIX (Enzynomics, Daejeon, Korea). A melting curve analysis was done to confirm amplicon specificity. Primer sequences are listed in Supplemental Table 4.

Western blot analysis

Tissues and cells were lysed using Tissue Protein Extraction Reagent or Mammalian Protein Extraction Reagent (#78510 or #78501, Thermo Fisher Scientific). Protein lysates were separated by SDS-PAGE and transferred to PVDF membranes. After blocking with 5% skim milk, blots were probed with primary antibodies targeting proteins of interest, followed by HRP-conjugated secondary antibodies. The information about the antibodies used is listed in Supplemental Table 5. Immunoreactive protein bands were visualized using a chemiluminescence system

(#WBKLS0500, Millipore, Merck KGaA) and the Fusion Solo S instrument (Vilber, Collégien, France).

Bulk and single-nucleus RNA-sequencing

For the MASLD cohort, liver tissues from 293 participants were used for total RNA isolation, followed by bulk RNA-seq, as previously described (1). Briefly, total RNA isolated from the liver tissue was used for sequencing on a HiSeq2500 platform. Reads were mapped and quantified using the human genome (hg19/GRCh37) based on GENCODE v19. Differentially expressed genes (DEGs) were identified using the DESeq2 packages (2), with adjustments for batch effects. snRNA-seq data were accessed from the NIH Bioproject PRJNA1221860 (GEO ID: GSE289173). The dataset includes 25 liver biopsy samples from MASLD patient. Cell type annotations and patient information were obtained from the associated paper (3).

For the murine model, RNA was extracted from liver tissues, and the total RNA concentration was assessed using the Quant-IT RiboGreen assay (#R11490, Invitrogen). RNA integrity was determined using the TapeStation RNA ScreenTape system (#5067-5576, Agilent Technologies), and only samples with a RIN exceeding 7.0 were selected for RNA library preparation. Library construction was performed independently for each sample using 0.5 μg of total RNA and the Illumina TruSeq Stranded Total RNA Library Prep Gold Kit (#20020599, Illumina, San Diego, CA, USA). The library preparation process began with rRNA removal, followed by mRNA fragmentation using divalent cations under elevated temperatures. The fragmented RNA was then used for first-strand cDNA synthesis with SuperScript II reverse transcriptase (#18064014, Invitrogen) and random primers. Second-strand cDNA synthesis was carried out using DNA Polymerase I, RNase H, and dUTP. The resulting cDNA underwent end repair, addition of a single

"A" nucleotide, and adapter ligation. After PCR amplification, the libraries were purified, quantified, and validated using KAPA Library Quantification kits for Illumina Sequencing platforms and the TapeStation D1000 ScreenTape (#5067-5582, Agilent Technologies). The indexed libraries were subsequently sequenced in a paired-end format (2 × 100 bp) on an Illumina NovaSeq platform (Illumina) by Macrogen (Seoul, Korea).

Relative gene abundance was determined in read counts using StringTie. To identify DEGs, statistical analysis was conducted based on gene abundance estimates for each sample. Genes with more than one instance of zero read count were excluded. To enable log2 transformation, a value of 1 was added to each filtered gene's read count. The filtered data were then log2-transformed and normalized using TMM method. Statistical significance was assessed using exactTest in edgeR, based on fold changes and the assumption of no differences between groups. The false discovery rate (FDR) was controlled by adjusting *P*-values with the Benjamini–Hochberg method. Hierarchical clustering of DEGs was conducted using complete linkage and Euclidean distance metrics. Functional annotation, gene enrichment, and pathway analyses for significant gene sets were performed using gProfiler (https://biit.cs.ut.ee/gprofiler/gost) and the Kyoto Encyclopedia of Genes and Genomes (KEGG) pathway database (http://www.genome.jp/kegg/pathway.html). For gene set enrichment analysis (GSEA), RNA-Seq data were analyzed using GSEA 4.1.0 software and hallmark gene sets from the Molecular Signatures Database (http://software.broadinstitute.org/gsea/msigdb) v7.4. FDR was used to determine the statistical significance of normalized enrichment scores (NES), with gene sets considered significant at FDR < 0.25.

Transient transfection and luciferase reporter assay

Human *FETUA* and *LECT2* promoter reporter vectors were obtained from GeneCopoeia (Rockville, MD, USA). Cells were transfected with each reporter construct along with either a Mock or FoxO1 overexpressing plasmid using Lipofectamine 2000 (#11668027, Invitrogen, Thermo Fisher Scientific). The promoter activities were assessed using the Secrete-Pair Dual Luminescence Assay Kit (#LF032, GeneCopoeia), which enables the quantification of *Gaussia* Luciferase (GLuc) and Secreted Alkaline Phosphatase (SEAP) activities in cell culture media.

ChIP assay

ChIP assays were performed using the Enzymatic Chromatin IP kit (#9003, Cell Signaling Technology). Cells were fixed with 1% formaldehyde for 15 minutes at room temperature to cross-link chromatin complexes, then quenched with 125 mM glycine. Following cell lysis, chromatin was harvested, fragmented, and immunoprecipitated overnight at 4°C with antibodies against FoxO1 (#2880, Cell Signaling Technology) or nonspecific IgG (#2729, Cell Signaling Technology). Data were normalized to input levels, and primer sequences for ChIP are listed in Supplemental Table 4.

Measurement of Fetuin-A and LECT2

Serum levels of Fetuin-A and LECT2 were measured using commercially available ELISA kits (mouse Fetuin-A, #MFTA00, R&D Systems, Minneapolis, MN, USA; mouse LECT2, #MBS2705836, MyBioSource, San Diego, CA, USA; human Fetuin-A, #CSB-E12882h, Cusabio, Houston, TX, USA; human LECT2, #CSB-EL012855HU, Cusabio) according to the manufacturers' instructions.

Hepatokine treatment experiments

Differentiated 3T3-L1 adipocytes or C2C12 myotubes were treated with exogenous Fetuin-A (100 μg/mL; # F3004, Sigma-Aldrich), and/or LECT2 (200 ng/mL; # CSB-MP012855MO, Cusabio) for 3 hours, followed by insulin stimulation.

Iron modulation experiments

For pharmacological iron chelation, following 4 weeks of HFD feeding, mice were administered vehicle (PBS) or DFP (100 mg/kg; #379409, Sigma-Aldrich) via oral gavage six days per week for 5 weeks while remaining on HFD. For iron loading, after 2 weeks of HFD feeding, mice received intravenous injections of iron dextran at 2-hour intervals for a total of three administrations. Metabolic assessments were performed 24 h after the final injection.

References

1. Yoo T, et al. Disease-specific eQTL screening reveals an anti-fibrotic effect of AGXT2 in non-alcoholic fatty liver disease. *J Hepatol.* 2021;75(3):514-523.
2. Love MI, et al. Moderated estimation of fold change and dispersion for RNA-seq data with DESeq2. *Genome Biol.* 2014;15(12):550.
3. Hong SE, et al. Single-cell eQTL analysis identifies genetic variation underlying metabolic dysfunction-associated steatohepatitis. *Nat Genet.* 2025;57(7):1638-1648.

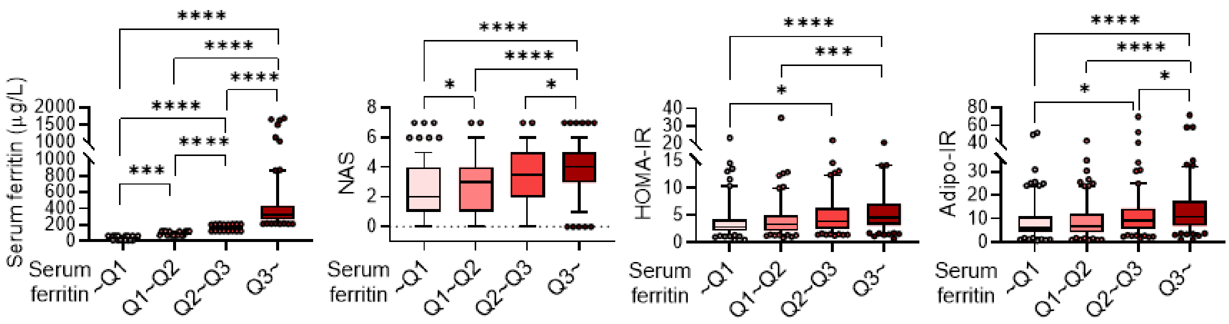

Supplemental Figure 1. Association of metabolic parameters with serum ferritin quartiles.

Analyses of NAS, HOMA-IR, and Adipo-IR stratified by serum ferritin quartiles. Box-and-whisker plots show the median, interquartile range, and 5th–95th percentiles. Statistical analyses were performed using one-way ANOVA followed by Tukey's multiple comparisons test. *p < 0.05, ***p < 0.001, ****p < 0.0001.

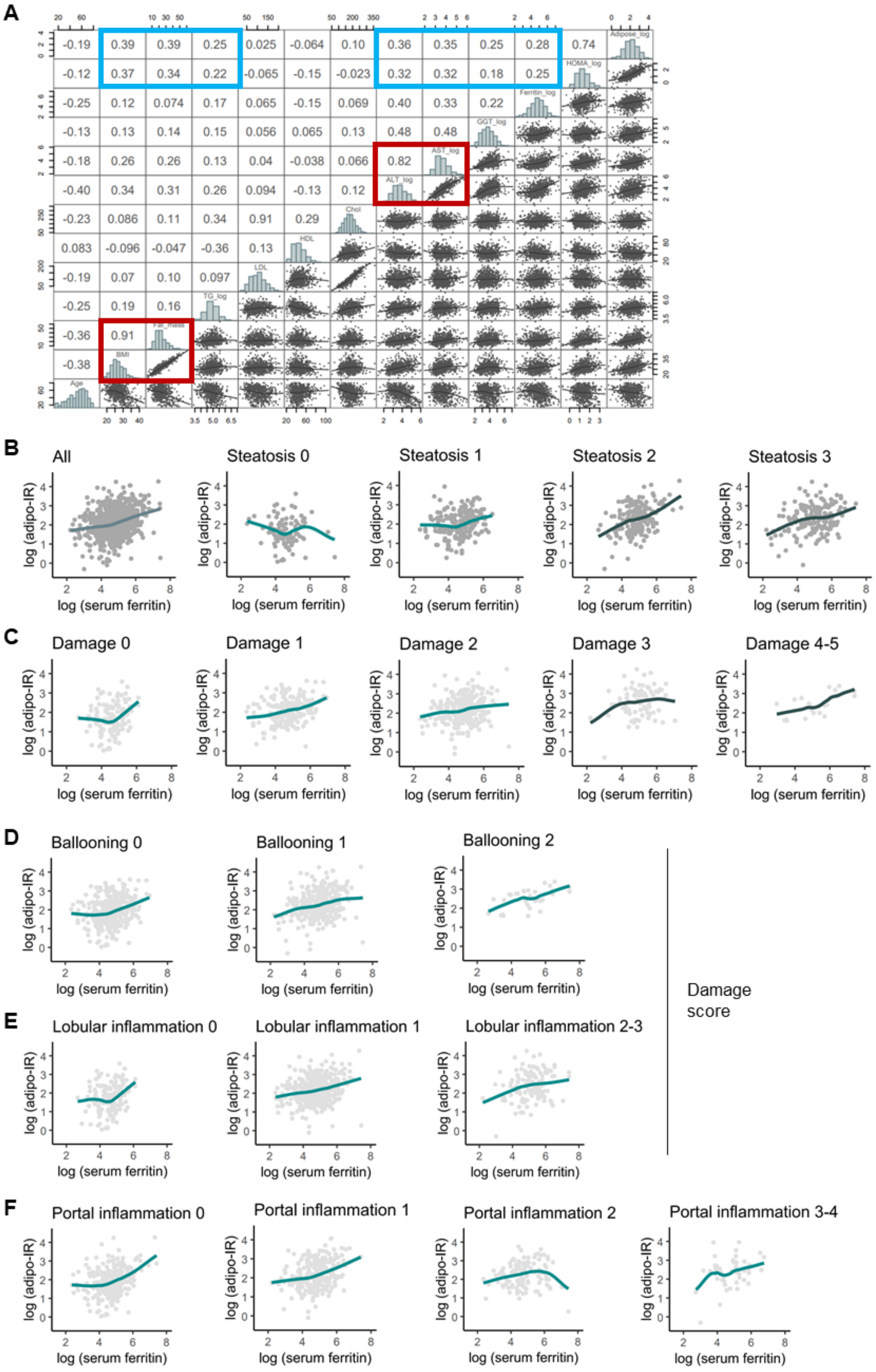

A
B
All
Steatosis 0
Steatosis 1
Steatosis 2
Steatosis 3
C
Damage 0
Damage 1
Damage 2
Damage 3
Damage 4-5
D
Ballooning 0
Ballooning 1
Ballooning 2
E
Lobular inflammation 0
Lobular inflammation 1
Lobular inflammation 2-3
Damage score
F
Portal inflammation 0
Portal inflammation 1
Portal inflammation 2
Portal inflammation 3-4
log (adipo-IR)
log (serum ferritin)

Supplemental Figure 2. Relationship between clinicopathological variables and insulin resistance.

(**A**) Pearson's correlation coefficients between clinicopathological variables and insulin resistance parameters in MASLD patients. Blue rectangles indicate variables with a correlation coefficient of 0.25 or higher with insulin resistance (either adipo-IR or HOMA-IR), which were considered covariates in the regression analysis. Red rectangles indicate a very high correlation between two given variables. To avoid multicollinearity, only one of the variables with a higher correlation coefficient was selected from each red rectangle.

(**B**–**C**) Association between serum ferritin and adipo-IR according to steatosis severity (**B**) or liver damage status (**C**).

(**D**–**F**) Scatter plots with LOESS lines illustrating the relationship between serum ferritin and adipo-IR based on hepatocyte ballooning (**D**), lobular inflammation (**E**), and portal inflammation levels (**F**).

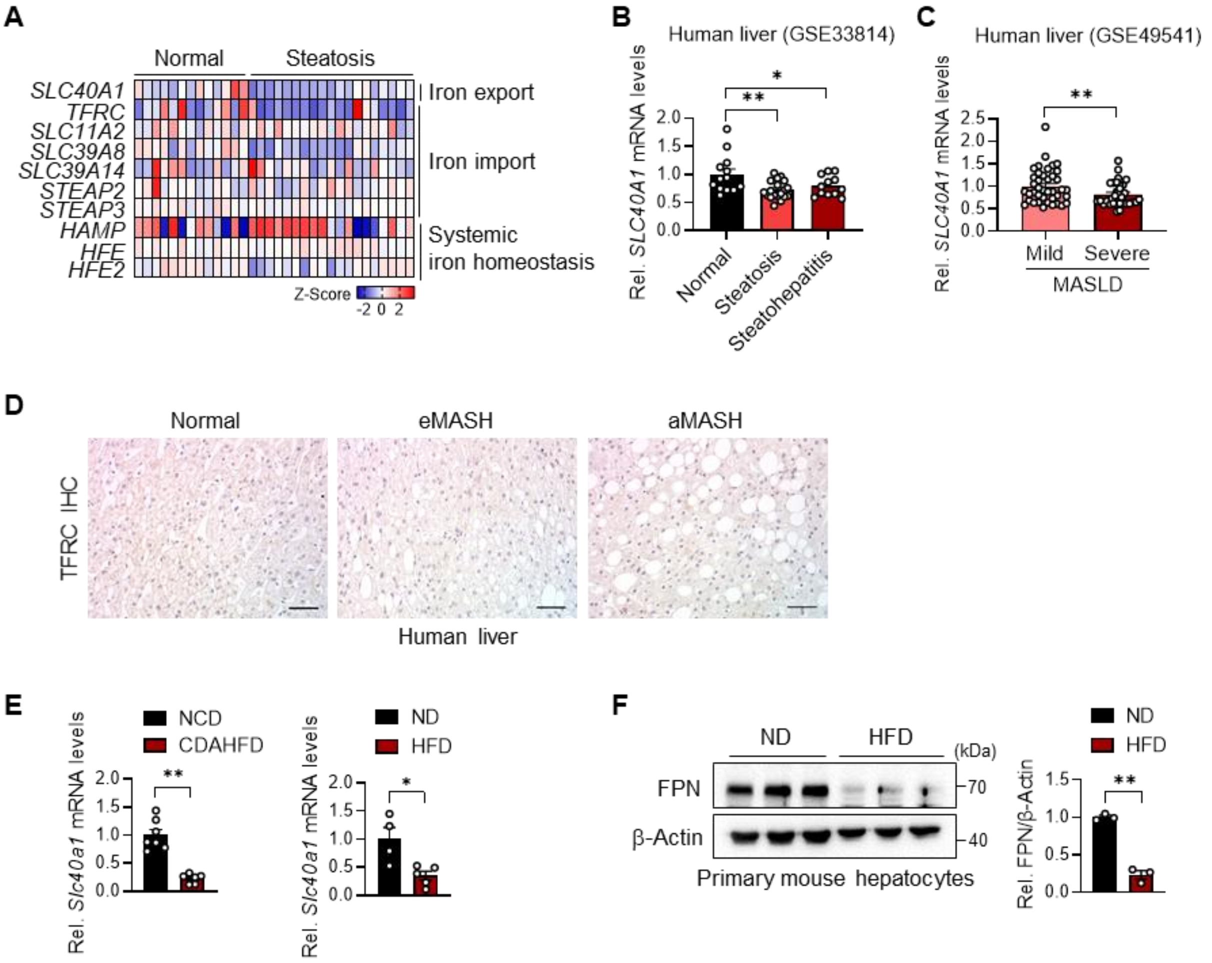

Supplemental Figure 3. Identification of FPN as a key iron-regulatory molecule altered in MASLD.

(**A**) Heatmap depicting hepatic gene expression related to iron homeostasis in healthy controls versus patients with hepatic steatosis (GSE33814).

(**B**–**C**) Hepatic FPN mRNA expression comparing healthy controls (*n* = 13), steatosis (*n* = 19), and steatohepatitis (*n* = 12) patients (**B**, GSE33814), as well as mild (*n* = 40) vs. severe MASLD (*n* = 32) cases (**C**, GSE49541).

(**D**) Immunohistochemical staining of TFRC in liver tissues from normal controls and patients with MASLD. eMASH, early MASH defined as MASH with no or mild fibrosis (F0–1); aMASH,

advanced MASH defined as MASH with fibrosis stage F ≥ 2. Scale bars: 20 μm.

(**E**) Hepatic Fpn mRNA levels in mice fed CDAHFD (*n* = 6–7 per group) or HFD (*n* = 4–5 per group).

(**F**) Western blot analysis of FPN in primary hepatocytes isolated from HFD-fed mice (*n* = 3 per group).

Data are presented as mean ± SEM. Statistical analyses were conducted using unpaired two-tailed Student's *t*-tests (**C**, **E**, and **F**) or one-way ANOVA followed by Tukey's multiple comparisons test (**B**). $*p < 0.05$, $**p < 0.01$.

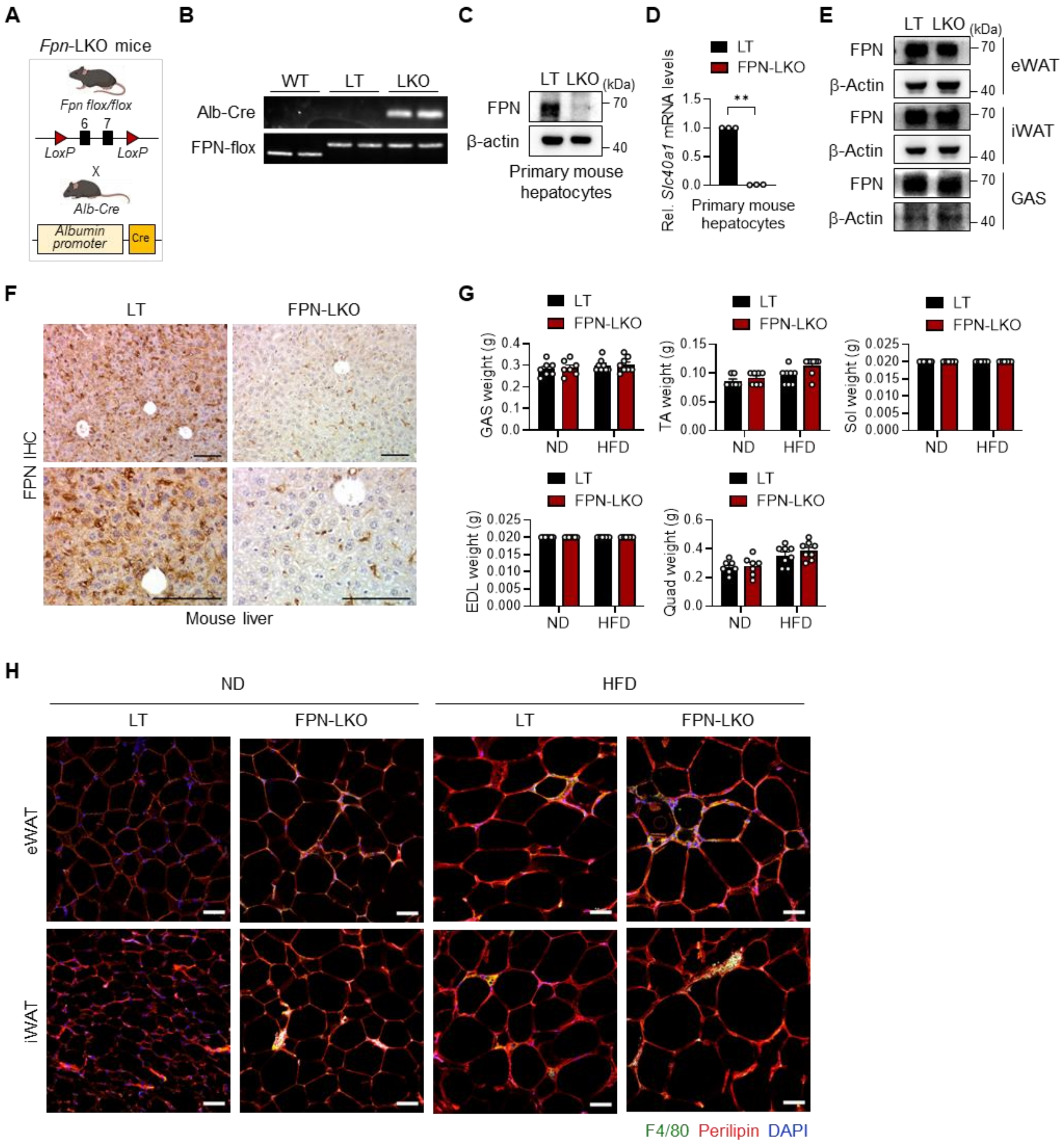

Supplemental Figure 4. Generation of hepatocyte-specific FPN-deficient mice.

(**A**) Strategy for generating *Fpn*-LKO mice by crossing *Fpn*$^{flox/flox}$ and *Alb-Cre* mice.

(**B**) Genotyping of *Fpn*-LKO mice.

(**C**–**E**) Validation of hepatocyte-specific FPN deletion in primary hepatocytes and other metabolic tissues from *Fpn*-LKO mice.

(**F**) Immunohistochemical staining of FPN confirming hepatocyte-specific FPN deletion. Scale bars represent 20 μm (upper images) or 50 μm (lower images).

(**G**) Skeletal muscle weights of LT control and *Fpn*-LKO mice fed ND or HFD (n = 7–8 per group).

(**H**) Immunostaining of F4/80 and perilipin in eWAT and iWAT. Scale bars: 50 μm.

Data are presented as mean ± SEM. Statistical analyses were conducted using unpaired two-tailed Student's t-tests. **$p < 0.01$.

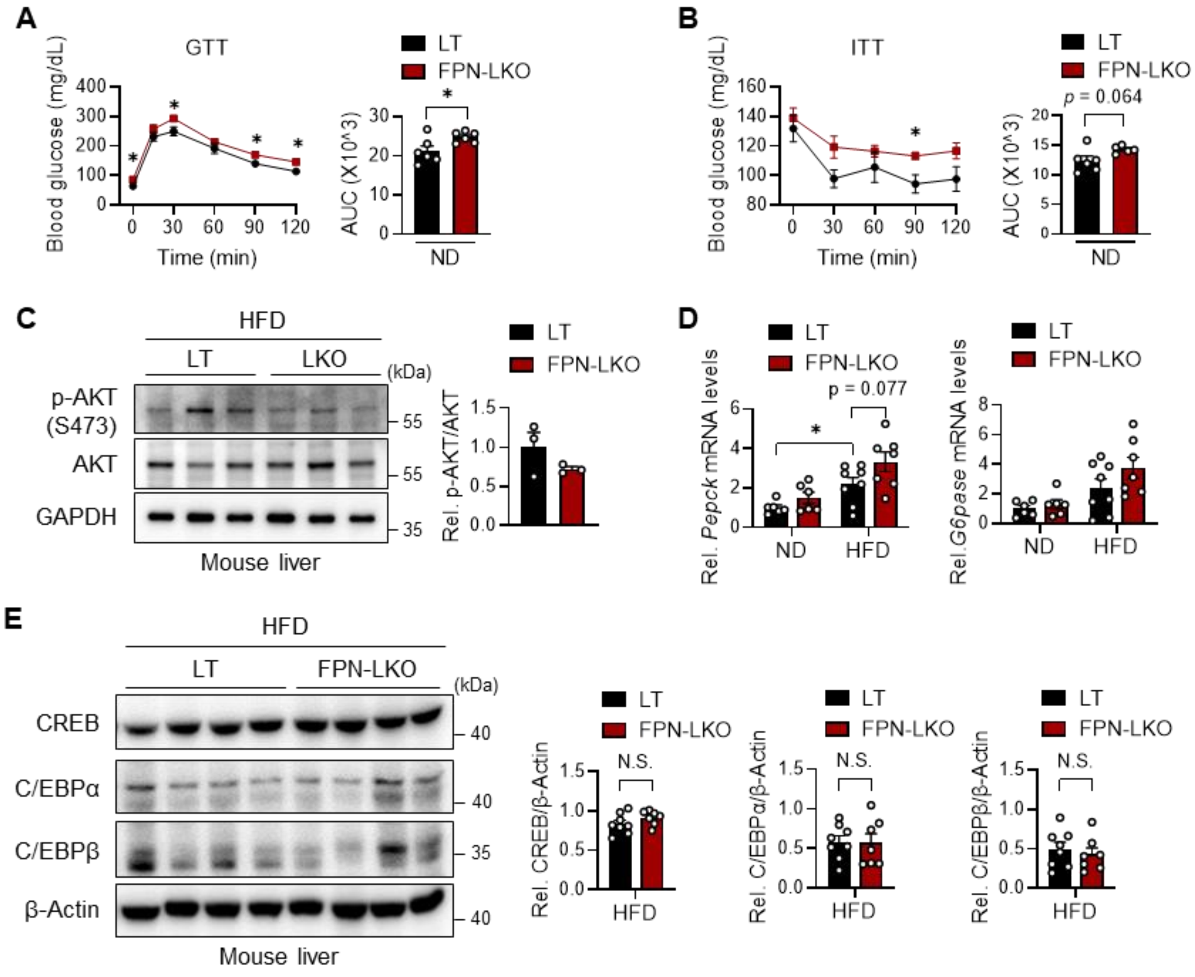

Supplemental Figure 5. No changes in hepatic gluconeogenesis in Fpn-LKO mice.

(**A**) GTT analysis in the ND-fed mice (n = 6 per group).

(**B**) ITT analysis in the ND-fed mice (n = 5–6 per group).

(**C**) Western blot analysis of p-Akt (S473) in liver tissues from the HFD-fed mice. The relative band intensities were quantified.

(**D**) qPCR analysis for hepatic gluconeogenic enzyme genes (n = 6–8 per group).

(**E**) Western blot analysis of transcriptional regulators of gluconeogenesis (n = 7–8 per group).

Data are presented as mean ± SEM. Statistical analyses were conducted using unpaired two-tailed Student's *t*-tests. *$p < 0.05$, N.S., not significant.

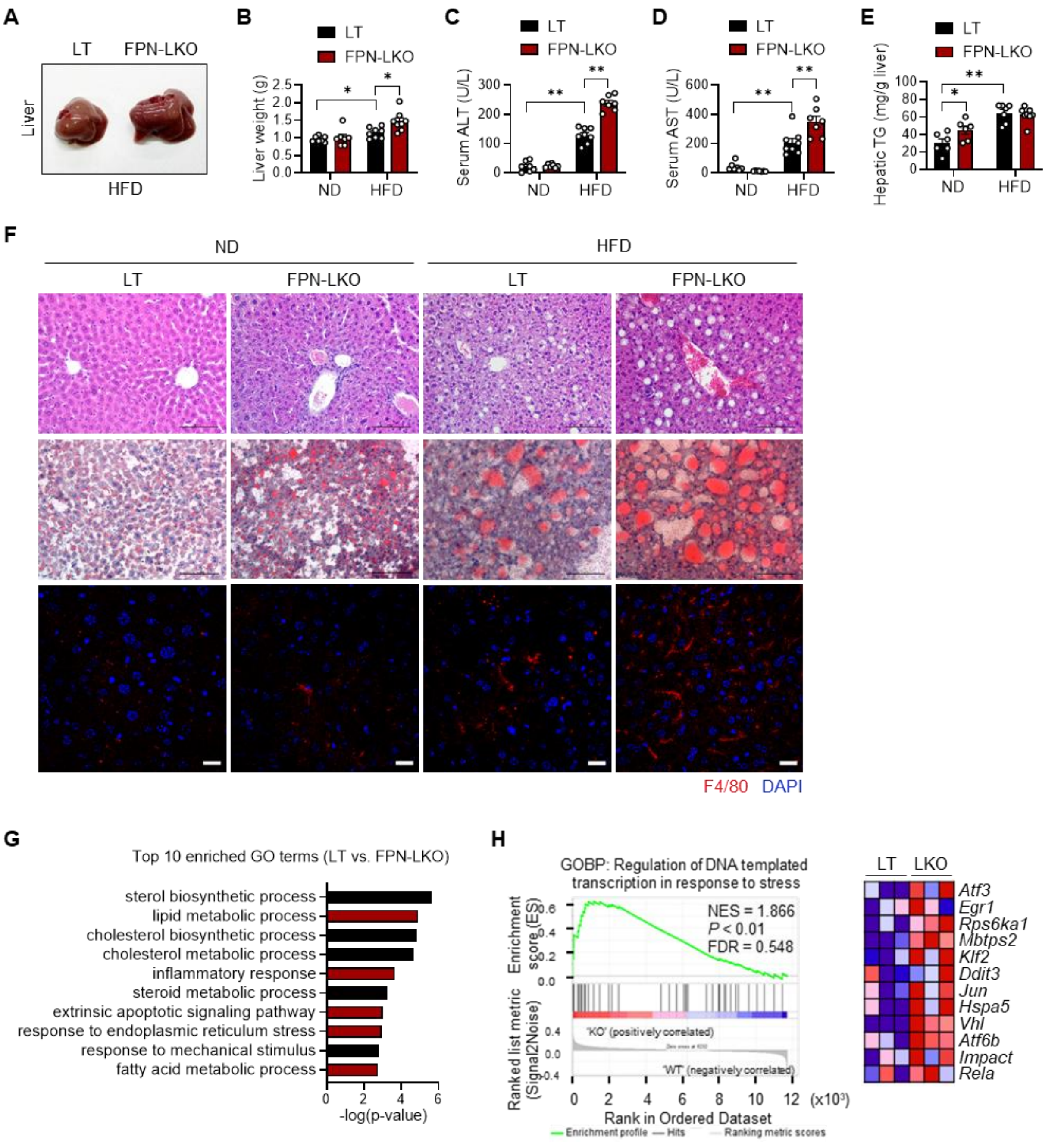

Supplemental Figure 6. Steatohepatitis development in Fpn-LKO mice.

(**A**–**B**) Gross morphology and liver weights of LT control and *Fpn*-LKO mice fed ND or HFD (n = 7–8 per group).

(**C**–**D**) Serum ALT and AST activities (n = 7–8 per group).

(**E**) Hepatic triglyceride (TG) levels (n = 6–8 per group).

(**F**) H&E and Oil Red O staining of liver sections. Scale bars: 50 μm. Immunostaining for F4/80 in liver sections. Scale bars: 20 μm.

(**G**–**H**) Gene ontology (GO) and gene set enrichment analysis (GSEA) plots based on liver RNA-seq data from LT control and *Fpn*-LKO mice (n = 3 per group).

Data are presented as mean ± SEM. Statistical analyses were conducted using unpaired two-tailed Student's *t*-tests. *$p < 0.05$, **$p < 0.01$.

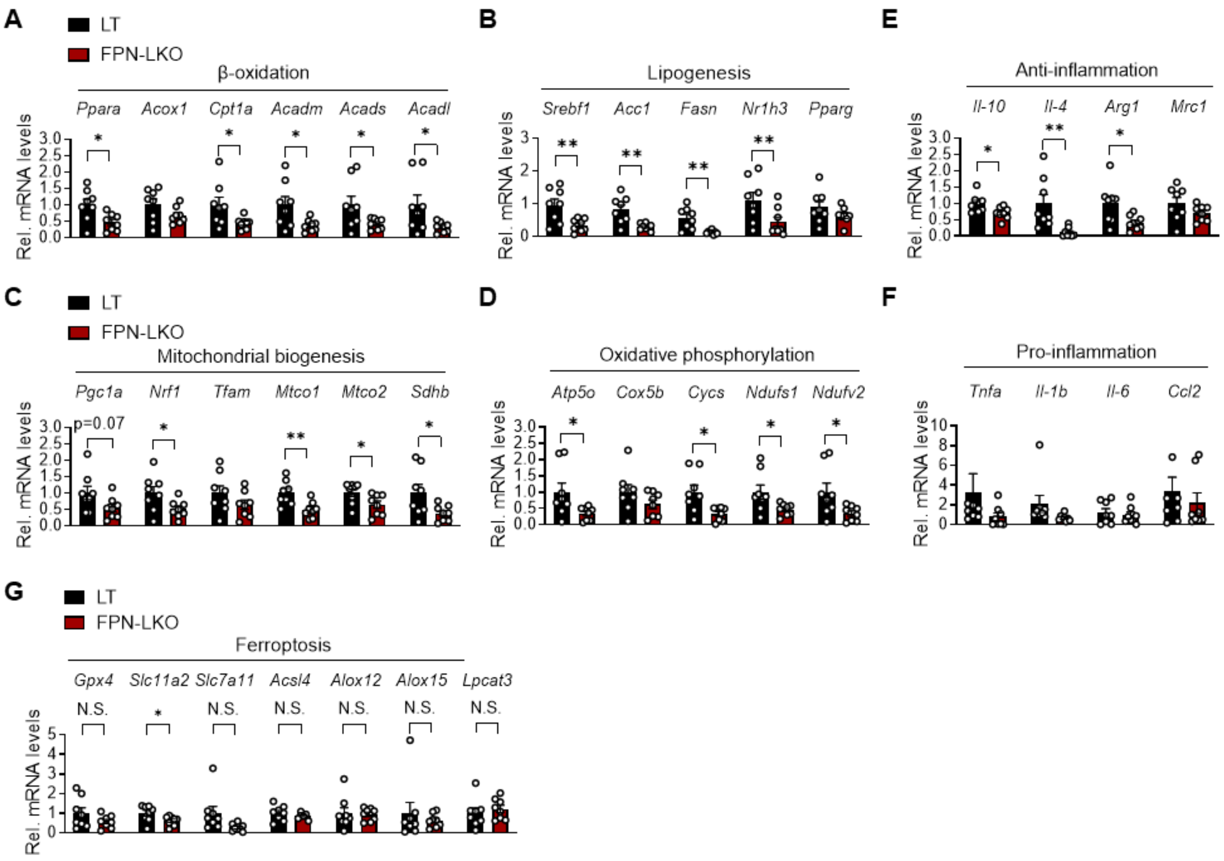

Supplemental Figure 7. Hepatic transcript levels in Fpn-LKO mice.

(**A**–**G**) qPCR analysis of genes involved in lipid metabolism (**A** and **B**), mitochondrial function (**C** and **D**), inflammation (**E** and **F**), and ferroptosis (**G**) (*n* = 8 per group).

Data are presented as mean ± SEM. Statistical analyses were conducted using unpaired two-tailed Student's *t*-tests. *p < 0.05, **p < 0.01. N.S., not significant.

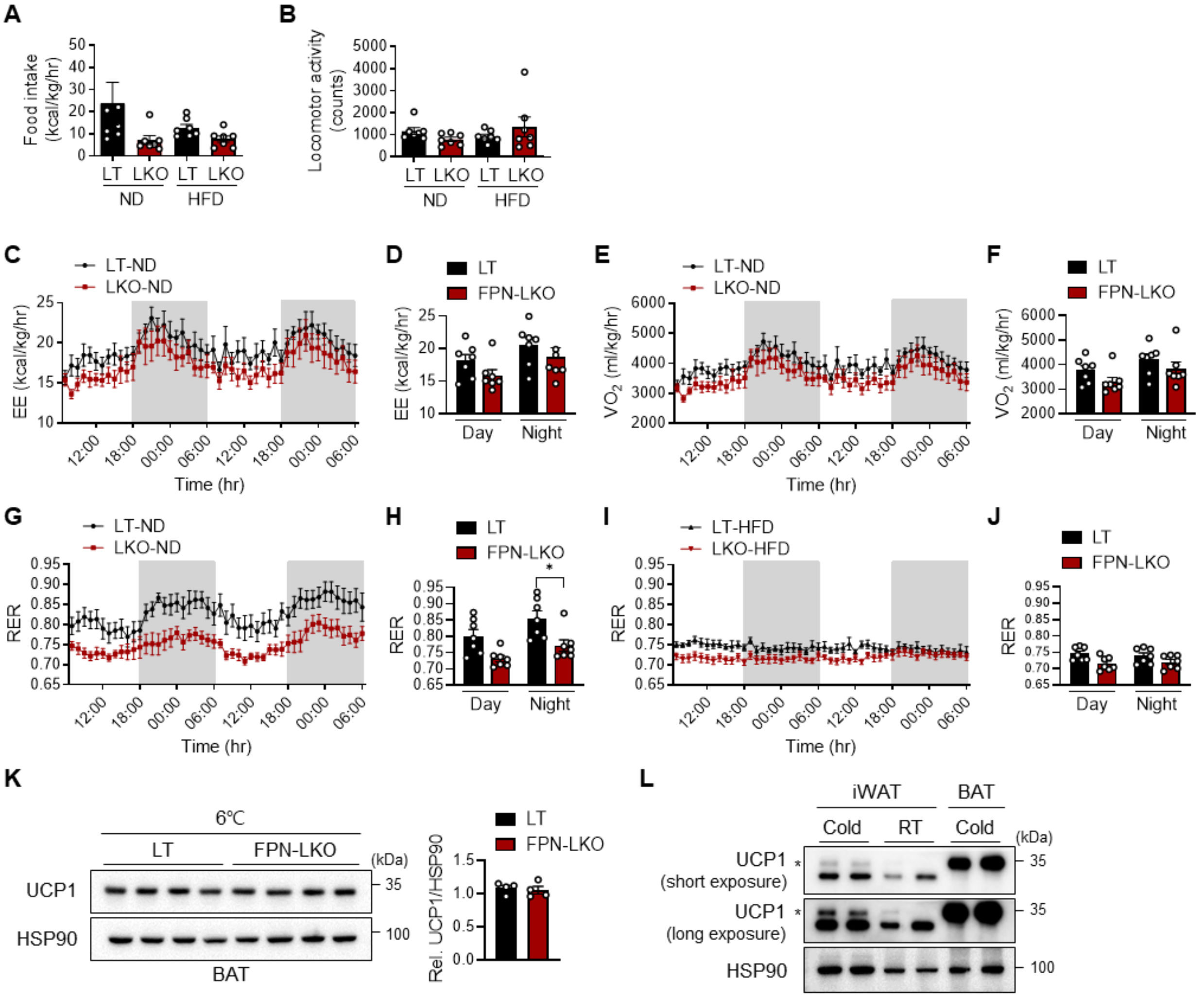

Supplemental Figure 8. No changes in food intake and locomotor activities in Fpn-LKO mice.

(**A**–**B**) Metabolic cage studies showing no significant differences in energy intake and physical activity of LT control versus *Fpn*-LKO mice fed ND or HFD (n = 7 per group).

(**C**–**F**) Energy expenditure and oxygen consumption rate in the ND-fed mice (n = 7 per group).

(**G**–**J**) Respiratory exchange ratio (RER) in the ND- or HFD-fed mice (n = 7 per group).

(**K**) Western blot analysis of UCP1 in BAT from mice following cold exposure at 6°C for 48 h. The relative band intensities were quantified.

(**L**) Western blot analysis of UCP1 in iWAT, with BAT used as a positive control.

Data are presented as mean ± SEM. Statistical analyses were conducted using unpaired two-tailed Student’s *t*-tests. *$p < 0.05$.

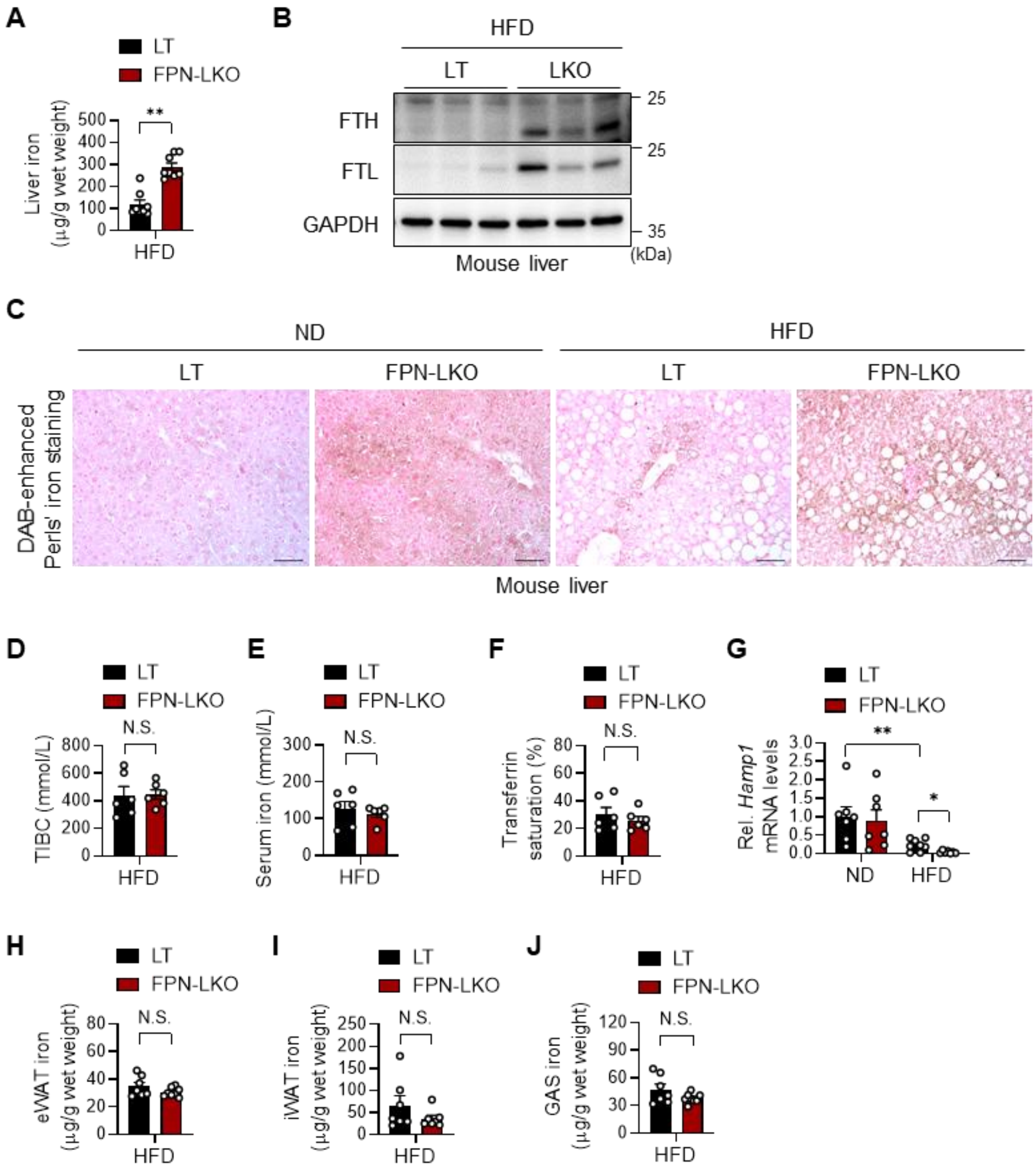

Supplemental Figure 9. Hepatic iron overload without alterations in iron levels of other metabolic tissues in Fpn-LKO mice.

(**A**–**B**) Hepatic iron accumulation evaluated by measuring liver iron content and performing Western blot analysis of ferritin heavy and light chain (FTH and FTL) expression in HFD-fed LT and *Fpn*-LKO mice (*n* = 8 per group).

(**C**) DAB-enhanced Perls' staining confirming hepatic iron accumulation in *Fpn*-LKO mice. Scale bars: 20 μm.

(**D**–**F**) Measurement of total iron-binding capacity (TIBC), serum iron content, and transferrin saturation in the HFD-fed mice (n = 6 per group).

(**G**) qPCR analysis of hepatic hepcidin expression in LT and *Fpn*-LKO mice (n = 7–8 per group).

(**H**–**J**) Measurement of iron levels in eWAT (n = 7–8 per group), iWAT (n = 7 per group), and GAS tissues (n = 7–8 per group).

Data are presented as mean ± SEM. Statistical analyses were conducted using unpaired two-tailed Student's *t*-tests. *$p < 0.05$, **$p < 0.01$, N.S., not significant.

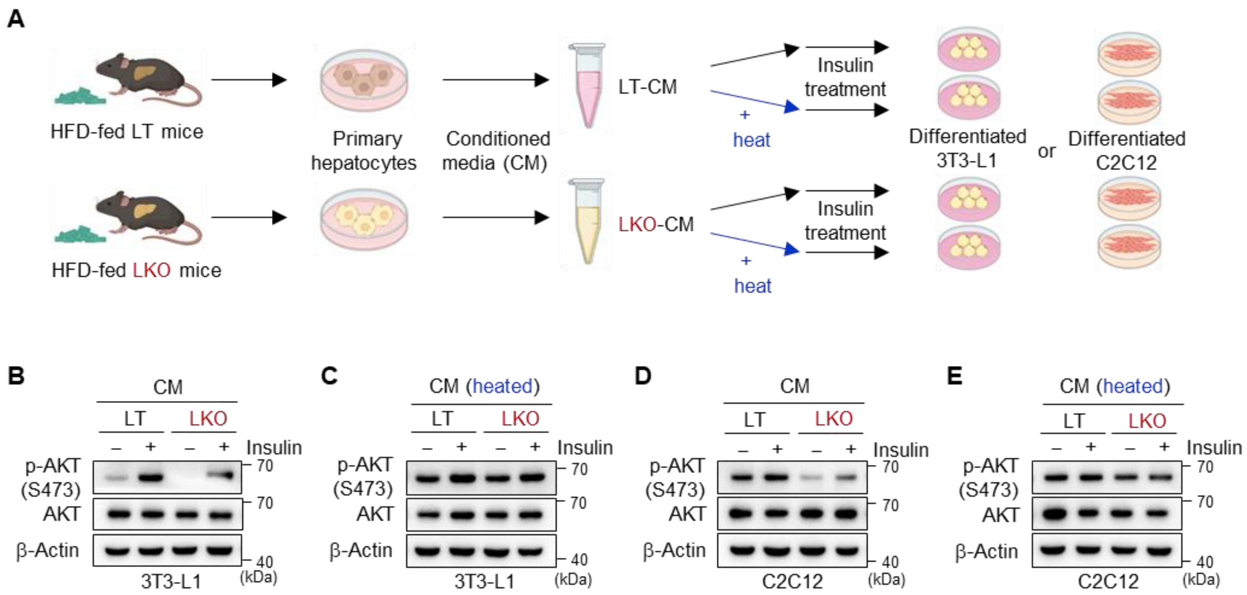

Supplemental Figure 10. Effects of conditioned media from FPN-deficient hepatocytes on insulin signaling in adipocytes and myotubes.

(**A**) Schematic of the experimental design to investigate potential inter-organ crosstalk between the liver and adipose tissue or skeletal muscle in metabolic dysfunction induced by hepatic FPN deficiency. Conditioned media (CM) were collected from primary hepatocytes of LT control and *Fpn*-LKO mice, and used either in their native form or after heat inactivation (90°C for 30 min). (**B**–**E**) Western blot analysis of p-Akt (S473) in differentiated 3T3-L1 adipocytes (**B**, **C**) and C2C12 myotubes (**D**, **E**) following insulin stimulation (10 nM, 15 min) after 24 h treatment with CM described in panel **A**.

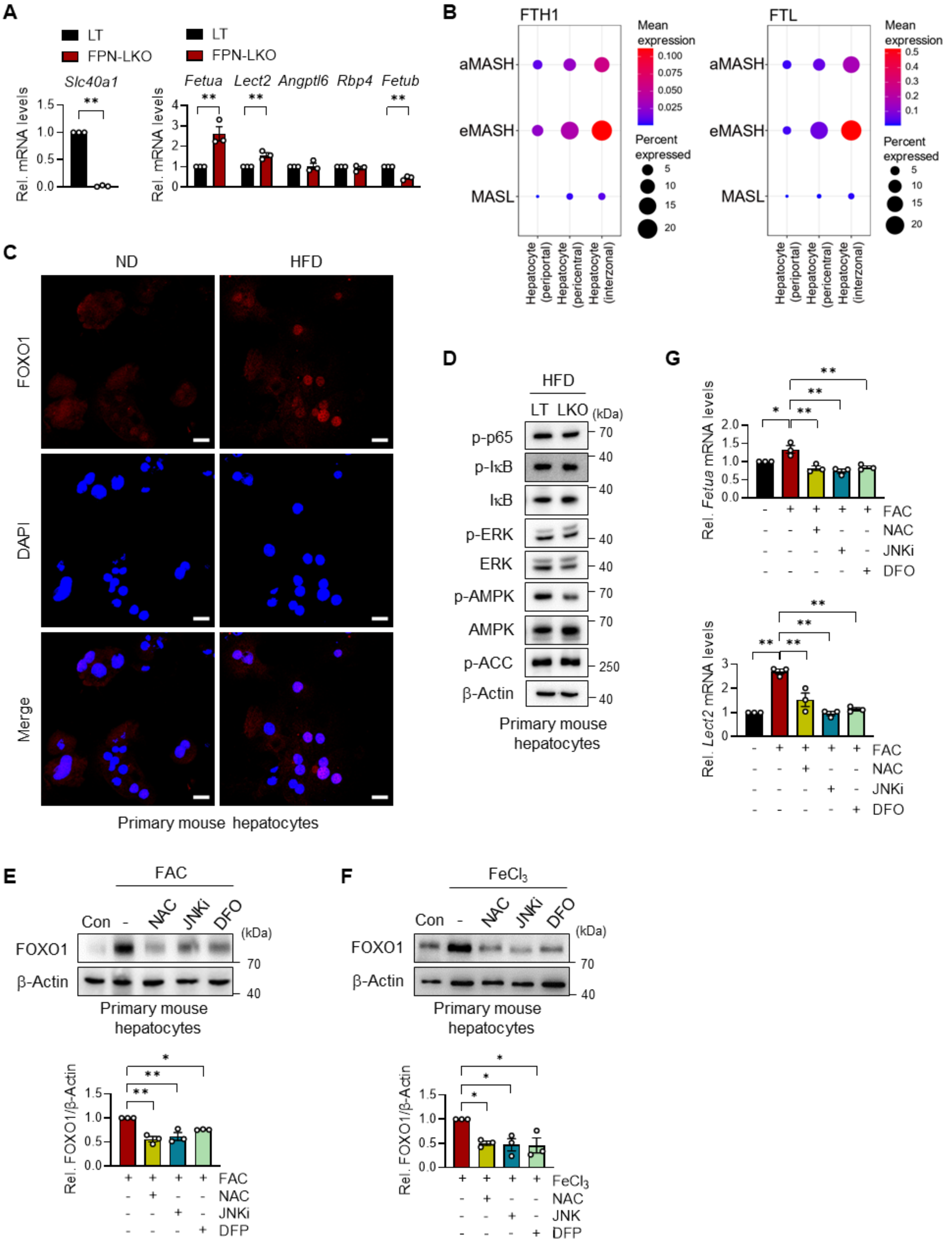
A
LT
FPN-LKO
Slc40a1
Fetua
Lect2
Angptl6
Rbp4
Fetub
Rel. mRNA levels
B
FTH1
FTL
aMASH
eMASH
MASL
Mean expression
Percent expressed
Hepatocyte (periportal)
Hepatocyte (pericentral)
Hepatocyte (interzonal)
C
ND
HFD
FOXO1
DAPI
Merge
Primary mouse hepatocytes
D
HFD
LT
LKO
(kDa)
p-p65
p-IκB
IκB
p-ERK
ERK
p-AMPK
AMPK
p-ACC
β-Actin
Primary mouse hepatocytes
G
Rel. Fetua mRNA levels
Rel. Lect2 mRNA levels
FAC
NAC
JNKi
DFO
E
FAC
Con
NAC
JNKi
DFO
FOXO1
β-Actin
Primary mouse hepatocytes
Rel. FOXO1/β-Actin
FAC
NAC
JNKi
DFP
F
FeCl3
Con
NAC
JNKi
DFO
FOXO1
β-Actin
Primary mouse hepatocytes
Rel. FOXO1/β-Actin
FeCl3
NAC
JNK
DFP

Supplemental Figure 11. Iron-induced FoxO1 expression via ROS-dependent JNK signaling.

(**A**) qPCR validation of mRNA expression of the indicated hepatokines in primary hepatocytes isolated from HFD-fed LT and *Fpn*-LKO mice (n = 3 per group).

(**B**) snRNA-seq analysis showing hepatocyte FTH1 and FTL expression in patients with MASLD according to disease severity.

(**C**) Immunofluorescence staining indicating FoxO1 activation in primary hepatocytes from HFD-fed mice compared to ND-fed mice. Scale bars: 20 μm.

(**D**) Western blot analysis of signaling pathway molecules known to regulate Fetuin-A or LECT2.

(**E**–**F**) Western blot analysis of FoxO1 in primary mouse hepatocytes treated with ferric ammonium citrate (FAC) or ferric chloride ($FeCl_3$), with or without pre-treatment with N-acetylcysteine (NAC, 10 mM), the JNK inhibitor (SP600125, 50 μM), or deferoxamine (DFO, 100 μM).

(**G**) qPCR analysis of Fetuin-A and LECT2 in primary mouse hepatocytes treated similarly to those in panel **E**.

Data are presented as mean ± SEM. Statistical analyses were conducted using unpaired two-tailed Student's *t*-tests (**A**) or one-way ANOVA followed by Tukey's multiple comparisons test (**E**–**G**). $^*p < 0.05$, $^{**}p < 0.01$.

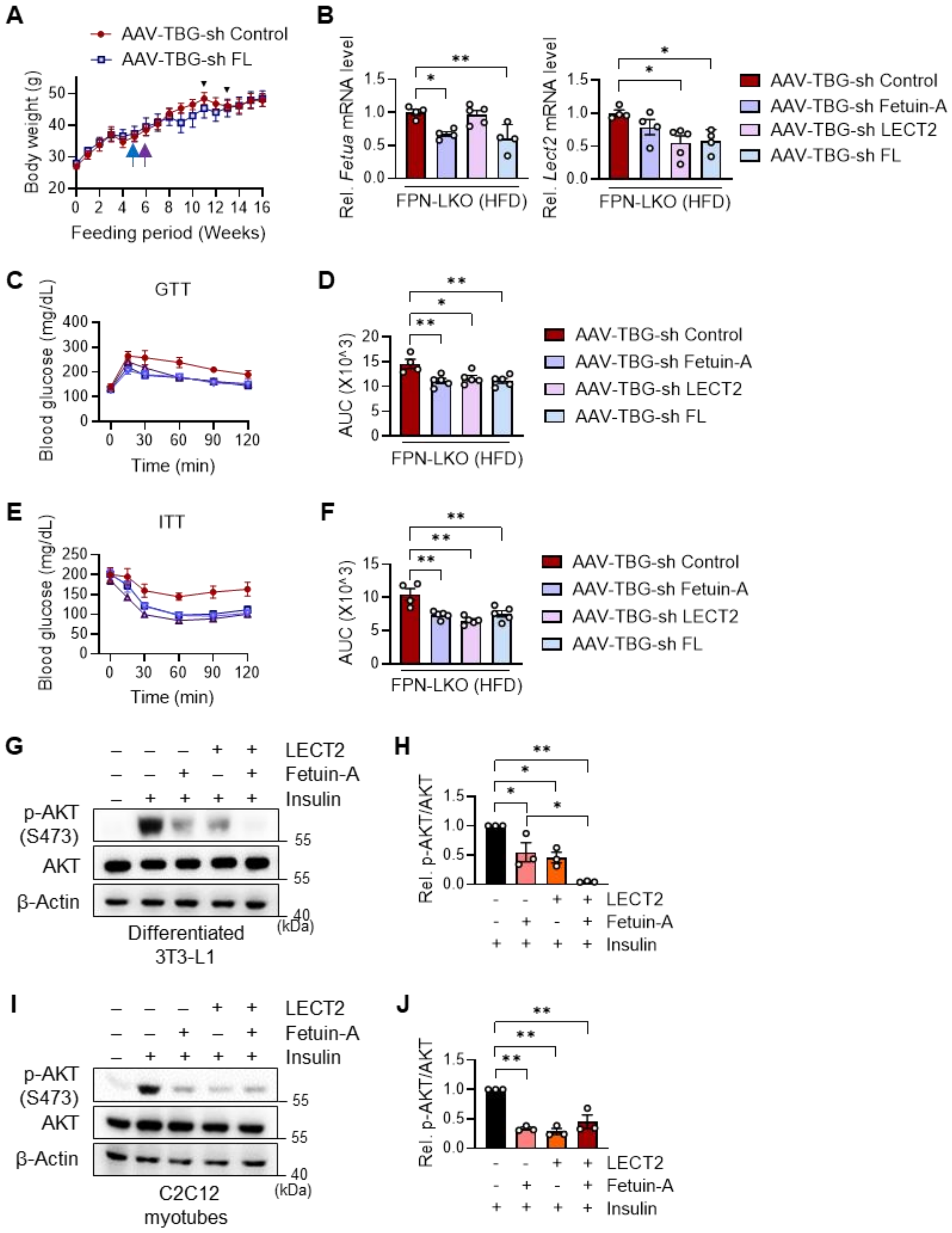

Supplemental Figure 12. Single and combined effects of Fetuin-A and LECT2 on insulin sensitivity.

(**A**) Body weight curves of Fpn-LKO mice injected with AAV vectors for hepatocyte-specific

knockdown of Fetuin-A and LECT2 (AAV-TBG-sh FL) (*n* = 6–7 per group). Arrows indicate the time points of AAV injection, and arrowheads indicate the timing of GTT and ITT analyses.

(**B**) qPCR analysis confirming selective knockdown of Fetuin-A and LECT2 (*n* = 4–5 per group).

(**C**–**D**) GTT analysis (*n* = 4–5 per group).

(**E**–**F**) ITT analysis (*n* = 4–5 per group).

(**G**–**J**) Effects of exogenous Fetuin-A and/or LECT2 on insulin signaling in differentiated 3T3-L1 adipocytes (**G** and **H**) and C2C12 myotubes (**I** and **J**) (*n* = 3 per group).

Data are presented as mean ± SEM. Statistical analyses were conducted using one-way ANOVA followed by Tukey's multiple comparisons test. *p < 0.05, **p < 0.01.

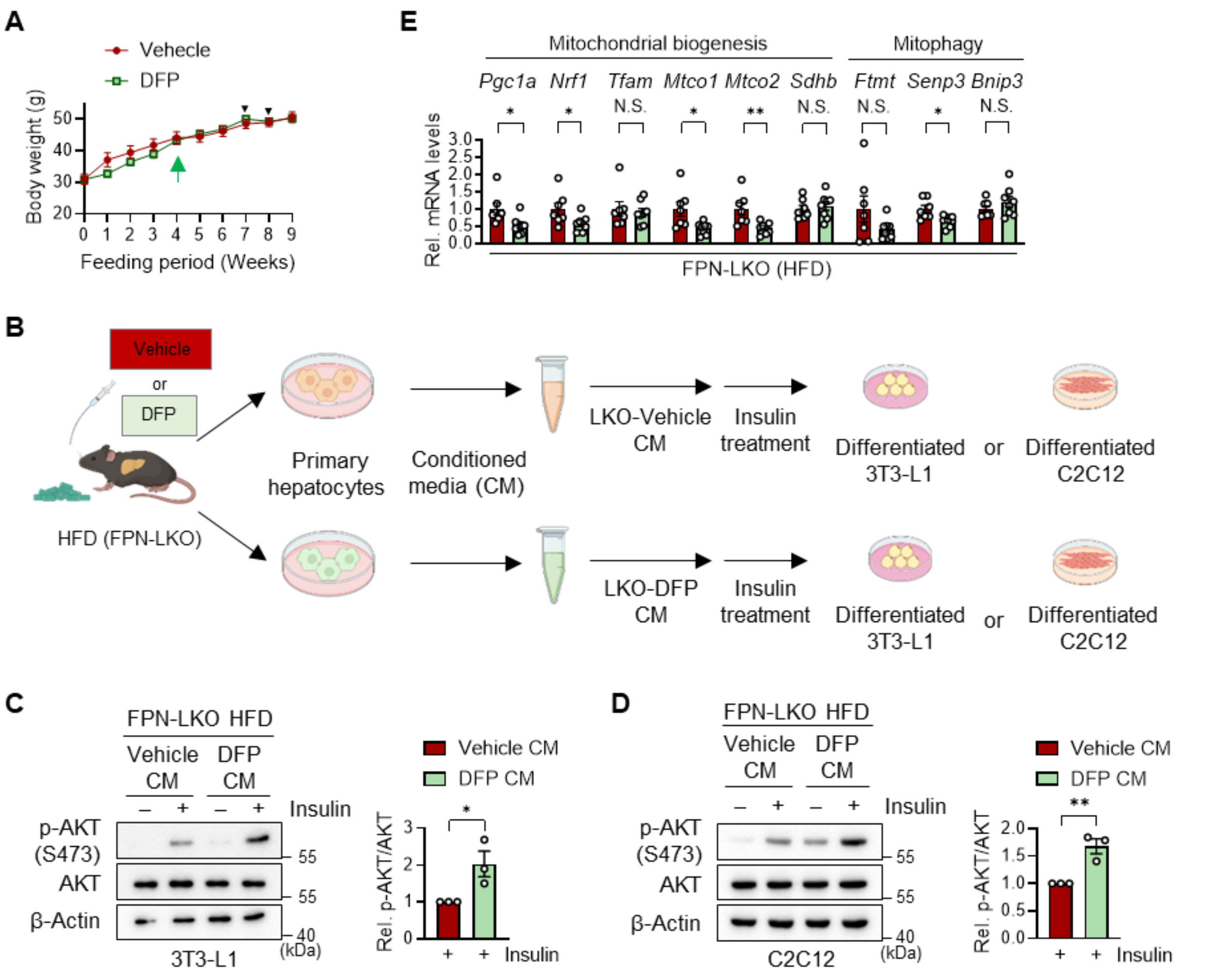

Supplemental Figure 13. Enhancement of insulin signaling by conditioned media from DFP-treated hepatocytes.

(**A**) Body weight curves of *Fpn*-LKO mice treated with deferiprone (DFP) during HFD feeding (*n* = 7–8 per group). Arrows indicate the time points of DFP administration, and arrowheads indicate the timing of GTT and ITT analyses.

(**B**) Experimental design to examine the effect of conditioned media (CM) collected from primary hepatocytes derived from *Fpn*-LKO mice treated with vehicle or DFP during HFD feeding on insulin signaling.

(**C**–**D**) Enhanced effects of CM from DFP-treated hepatocytes on insulin signaling in differentiated

3T3-L1 adipocytes (**C**) and C2C12 myotubes (**D**) (*n* = 3 per group).

(**E**) qPCR analysis of gene involved in mitochondrial biogenesis and mitophagy (*n* = 7–8 per group).

Data are presented as mean ± SEM. Statistical analyses were conducted using unpaired two-tailed Student's *t*-tests. *$p < 0.05$, **$p < 0.01$. N.S., not significant.

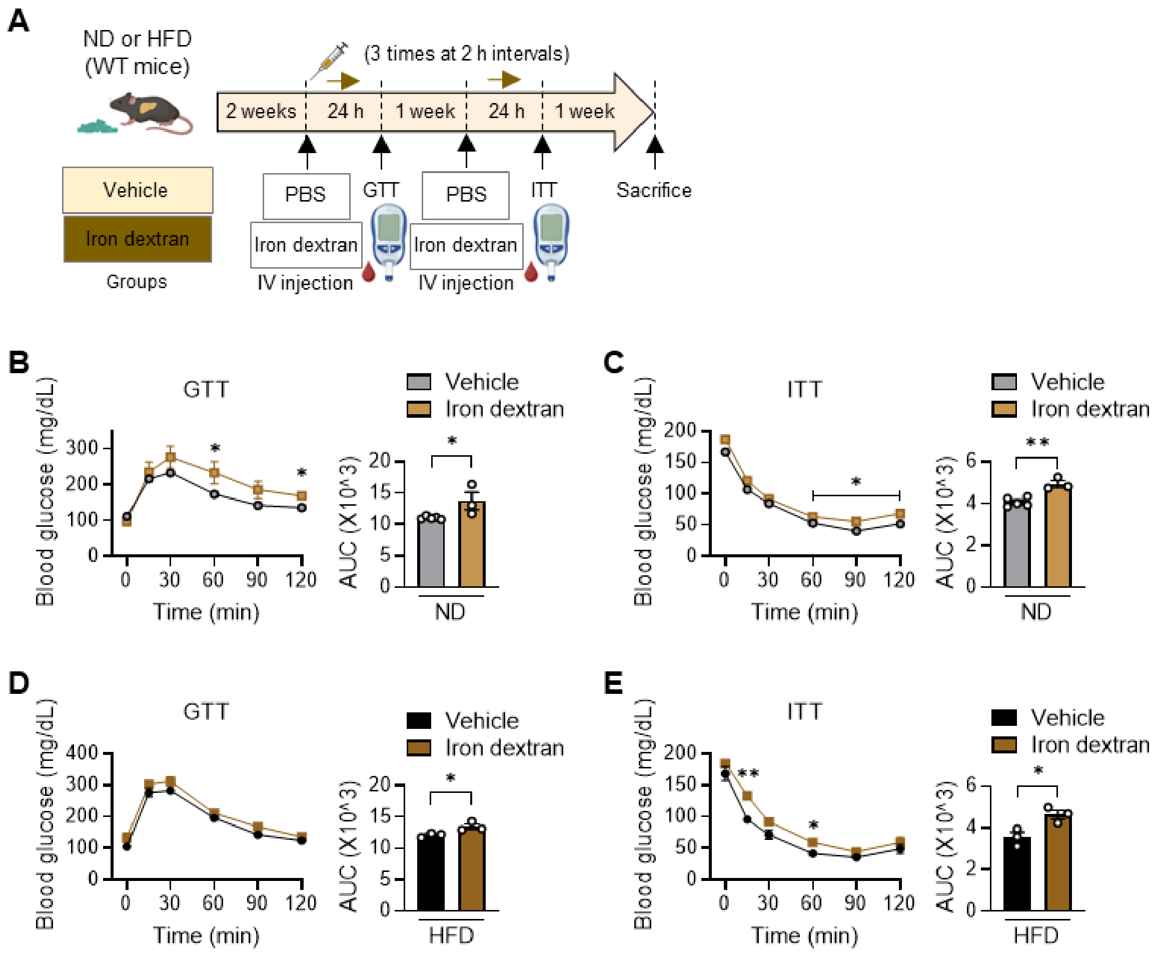

Supplemental Figure 14. Effect of iron dextran injection on insulin sensitivity in mice.

(**A**) Administration protocol of iron dextran in mice fed ND or HFD.

(**B**–**C**) GTT and ITT analyses in ND-fed mice ($n = 3–5$ per group).

(**D**–**E**) GTT and ITT analyses in HFD-fed mice ($n = 3$ per group).

Data are presented as mean ± SEM. Statistical analyses were conducted using unpaired two-tailed Student's *t*-tests. *$p < 0.05$, **$p < 0.01$.

Supplemental Table 1. Baseline characteristics of patients with MASLD.

Clinical and pathological characteristics			All (n=656)		Male (n=323)		Female (n=333)	
			Median	(IQR)	Median	(IQR)	Median	(IQR)
Clinical characteristics	Age (year)		56.0	(22.3)	50.0	(23.0)	60.0	(15.0)
	Adipo-IR		8.2	(8.5)	7.7	(7.4)	9.0	(9.6)
	HOMA-IR		3.7	(3.3)	3.5	(3.1)	3.9	(3.4)
	BMI (kg/m^2)		26.5	(5.3)	26.9	(5.0)	26.0	(5.5)
	Fat mass (kg)		21.7	(9.6)	20.5	(9.7)	22.3	(10.1)
	TG (mg/dL)		134.0	(84.0)	143.0	(88.0)	123.0	(70.0)
	LDL (mg/dL)		102.0	(49.9)	100.0	(52.0)	104.6	(46.8)
	HDL (mg/dL)		45.0	(15.3)	41.0	(15.0)	49.0	(16.0)
	Total cholesterol (mg/dL)		179.0	(54.3)	175.0	(56.0)	182.0	(54.0)
	ALT (IU/L)		40.0	(51.3)	47.0	(54.0)	35.0	(41.0)
	AST (IU/L)		36.0	(29.3)	36.0	(27.0)	37.0	(32.0)
	GGT (IU/L)		41.0	(54.0)	45.0	(58.5)	37.0	(46.0)
	Serum ferritin (ng/mL)		123.3	(142.5)	164.2	(166.3)	94.0	(112.2)
			n	(%)	n	(%)	n	(%)
Hepatic pathological index	Steatosis score	0	108	(16.5)	49	(15.2)	59	(17.7)
		1	186	(28.4)	98	(30.3)	88	(26.4)
		2	193	(29.4)	94	(29.1)	99	(29.7)
		3	169	(25.8)	82	(25.4)	87	(26.1)
	Lobular inflammation score	0	149	(22.7)	82	(25.4)	67	(20.1)
		1	389	(59.3)	197	(61.0)	192	(57.7)
		2	114	(17.4)	42	(13.0)	72	(21.6)
		3	4	(0.6)	2	(0.6)	2	(0.6)
	Portal inflammation score*	0	249	(38.0)	134	(41.5)	115	(34.5)
		1	246	(37.5)	132	(40.9)	114	(34.2)
		2	111	(16.9)	39	(12.1)	72	(21.6)
		3	40	(6.1)	14	(4.3)	26	(7.8)
		4	9	(1.4)	3	(0.9)	6	(1.8)
	Ballooning score	0	314	(47.9)	171	(52.9)	143	(42.9)
		1	309	(47.1)	136	(42.1)	173	(52.0)
		2	33	(5.0)	16	(5.0)	17	(5.1)
	NAS score	0	57	(8.7)	25	(7.7)	32	(9.6)
		1	90	(13.7)	55	(17)	35	(10.5)
		2	92	(14.0)	47	(14.6)	45	(13.5)
		3	109	(16.6)	55	(17)	54	(16.2)
		4	127	(19.4)	63	(19.5)	64	(19.2)
		5	126	(19.2)	54	(16.7)	72	(21.6)
		6	42	(6.4)	17	(5.3)	25	(7.5)
		7	13	(2.0)	7	(2.2)	6	(1.8)

* One data is missing in portal inflammation scores.

Supplemental Table 2. New stratification of hepatic pathological indices and age according to mean of natural log-transformed Adipo-IR and HOMA-IR values.

Covariate	Score or category	n	Log transformed Adipo-IR [log (Adipo-IR)]		Log transformed HOMA-IR [log (HOMA-IR)]		New category
			Mean	SD	Mean	SD	
Steatosis score	0	108	1.62	0.68	0.90	0.48	0-1
	1	186	2.00	0.66	1.30	0.56	0-1
	2	193	2.30	0.71	1.47	0.63	2-3
	3	169	2.32	0.72	1.49	0.64	2-3
Lobular inflammation score	0	149	1.71	0.73	1.05	0.53	0
	1	389	2.17	0.67	1.37	0.62	1
	2	114	2.40	0.77	1.57	0.64	2-3
	3	4	2.22	0.66	1.54	0.40	2-3
Portal inflammation score*	0	249	1.92	0.74	1.18	0.58	0
	1	246	2.16	0.71	1.37	0.61	1
	2	111	2.27	0.67	1.45	0.68	2-4
	3	40	2.40	0.77	1.64	0.62	2-4
	4	9	2.35	0.74	1.56	0.38	2-4
Ballooning score	0	314	1.91	0.72	1.17	0.59	0
	1	309	2.26	0.72	1.45	0.63	1
	2	33	2.51	0.51	1.70	0.49	2
NAS score	0	57	1.48	0.77	0.85	0.51	0
	1	90	1.80	0.63	1.08	0.50	1-2
	2	92	1.88	0.61	1.13	0.49	1-2
	3	109	2.34	0.63	1.55	0.64	3-5
	4	127	2.15	0.69	1.35	0.61	3-5
	5	126	2.31	0.72	1.51	0.61	3-5
	6	42	2.65	0.72	1.66	0.66	6-7
	7	13	2.51	0.54	1.77	0.57	6-7
Age	<19	10	2.54	0.55	1.71	0.45	< 30
	19-30	41	2.64	0.66	1.73	0.60	< 30
	30-40	76	2.24	0.80	1.37	0.67	≥ 30
	40-50	104	2.14	0.68	1.33	0.52	≥ 30
	50-60	159	2.00	0.66	1.18	0.54	≥ 30
	60-70	184	1.99	0.74	1.30	0.70	≥ 30
	>70	82	2.08	0.79	1.42	0.61	≥ 30

* One data is missing in portal inflammation scores.

Supplemental Table 3. Multiple linear regression analysis of the association between serum ferritin and Adipo-IR or HOMA-IR in MASLD.

	Model	Explanatory variables	log (Adipo-IR)			log (HOMA-IR)		
			Regression coefficient	p-value	Global F-test p-value	Regression coefficient	p-value	Global F-test p-value
All (n=655)	Model I	Sex (female vs. male)	0.213	< 0.0001	< 0.0001	0.140	0.0024	< 0.0001
		Age (< 30 vs.≥ 30)	-0.201	0.043		-0.143	0.1	
		BMI	0.044	< 0.0001		0.037	< 0.0001	
		log (TG)	0.207	0.00013		0.142	0.0027	
		log (ALT)	0.036	0.38		0.033	0.367	
		log (GGT)	0.064	0.055		0.004	0.88	
		log (Serum ferritin)	0.158	< 0.0001		0.120	< 0.0001	
		NAS (1-2 vs. 0)	0.169	0.079		0.117	0.17	
		NAS (3-5 vs. 0)	0.305	0.002		0.262	0.0026	
		NAS (6-7 vs. 0)	0.457	0.00058		0.331	0.0046	
		Portal inflammation (1 vs.0)	0.123	0.031		0.102	0.042	
		Portal inflammation (2-4 vs.0)	0.228	0.00077		0.217	0.00028	
	Model II	Sex (female vs. male)	0.223	< 0.0001	< 0.0001	0.154	0.00095	< 0.0001
		Age (< 30 vs. ≥ 30)	-0.204	0.42		-0.167	0.057	
		BMI	0.044	< 0.0001		0.037	< 0.0001	
		log (TG)	0.204	0.0002		0.140	0.0035	
		log (ALT)	0.050	0.24		0.046	0.22	
		log (GGT)	0.061	0.07		0.002	0.94	
		log (Serum ferritin)	0.164	< 0.0001		0.127	< 0.0001	
		Steatosis score (1 vs. 0)	0.146	0.065		0.211	0.0025	
		Steatosis score (2-3 vs. 0)	0.169	0.051		0.162	0.033	
		Ballooning score (1 vs. 0)	0.061	0.3		0.074	0.16	
		Ballooning score (2 vs. 0)	0.133	0.28		0.187	0.085	
		Lobular inflammation score (1 vs. 0)	0.101	0.14		0.043	0.47	
		Lobular inflammation score (2-3 vs. 0)	0.068	0.47		0.045	0.59	
		Portal inflammation (1 vs.0)	0.124	0.031		0.097	0.053	
		Portal inflammation (2-4 vs.0)	0.221	0.0017		0.197	0.0015	
Male (n=322)	Model I	Age (< 30 vs.≥ 30)	-0.122	0.29	< 0.0001	-0.029	0.79	< 0.0001
		BMI	0.046	< 0.0001		0.041	< 0.0001	
		log (TG)	0.339	< 0.0001		0.220	0.00096	
		log (ALT)	0.019	0.74		0.073	0.18	
		log (GGT)	0.043	0.35		-0.045	0.3	
		log (Serum ferritin)	0.176	< 0.0001		0.142	0.0003	
		NAS (1-2 vs. 0)	-0.001	0.995		-0.086	0.49	
		NAS (3-5 vs. 0)	0.157	0.25		0.021	0.879	
		NAS (6-7 vs. 0)	0.358	0.055		0.078	0.66	
		Portal inflammation (1 vs.0)	0.172	0.015		0.118	0.076	
		Portal inflammation (2-4 vs.0)	0.291	0.003		0.262	0.0048	
	Model II	Age (< 30 vs. ≥ 30)	-0.123	0.29	< 0.0001	-0.061	0.58	< 0.0001
		BMI	0.045	< 0.0001		0.041	< 0.0001	
		log (TG)	0.333	< 0.0001		0.210	0.0018	
		log (ALT)	0.023	0.7		0.088	0.118	
		log (GGT)	0.041	0.39		-0.051	0.248	
		log (Serum ferritin)	0.180	< 0.0001		0.148	0.00019	
		Steatosis score (1 vs. 0)	0.024	0.82		0.063	0.52	
		Steatosis score (2-3 vs. 0)	0.140	0.22		-0.025	0.82	
		Ballooning score (1 vs. 0)	0.081	0.28		0.095	0.18	
		Ballooning score (2 vs. 0)	0.254	0.13		0.299	0.056	
		Lobular inflammation score (1 vs. 0)	-0.006	0.94		0.034	0.68	
		Lobular inflammation score (2-3 vs. 0)	0.054	0.67		0.055	0.65	
		Portal inflammation (1 vs.0)	0.177	0.013		0.098	0.14	
		Portal inflammation (2-4 vs.0)	0.283	0.0058		0.201	0.037	
Female (n=333)	Model I	Age (< 30 vs.≥ 30)	-0.437	0.025	< 0.0001	-0.396	0.014	< 0.0001
		BMI	0.042	< 0.0001		0.034	< 0.0001	
		log (TG)	0.064	0.43		0.052	0.45	
		log (ALT)	0.057	0.34		0.008	0.87	
		log (GGT)	0.070	0.14		0.039	0.34	

	log (Serum ferritin)	0.137	0.0041		0.092	0.02	
	NAS (1-2 vs. 0)	0.295	0.038		0.237	0.044	
	NAS (3-5 vs. 0)	0.448	0.0019		0.459	0.00014	
	NAS (6-7 vs. 0)	0.535	0.0051		0.516	0.0012	
	Portal inflammation (1 vs.0)	0.060	0.51		0.070	0.36	
	Portal inflammation (2-4 vs.0)	0.162	0.096		0.167	0.039	
Model II	Age (< 30 vs. ≥ 30)	-0.477	0.015	< 0.0001	-0.411	0.012	< 0.0001
	BMI	0.043	< 0.0001		0.034	< 0.0001	
	log (TG)	0.078	0.35		0.055	0.43	
	log (ALT)	0.070	0.25		0.018	0.73	
	log (GGT)	0.070	0.15		0.044	0.28	
	log (Serum ferritin)	0.146	0.0021		0.101	0.01	
	Steatosis score (1 vs. 0)	0.258	0.035		0.341	0.00088	
	Steatosis score (2-3 vs. 0)	0.234	0.076		0.345	0.0017	
	Ballooning score (1 vs. 0)	0.030	0.75		0.055	0.48	
	Ballooning score (2 vs. 0)	-0.018	0.92		0.103	0.5	
	Lobular inflammation score (1 vs. 0)	0.201	0.065		0.048	0.59	
	Lobular inflammation score (2-3 vs. 0)	0.111	0.43		0.017	0.89	
	Portal inflammation (1 vs.0)	0.066	0.48		0.071	0.36	
	Portal inflammation (2-4 vs.0)	0.158	0.12		0.166	0.049	

Supplemental Table 4. Primer sequences used for qPCR, ChIP, and genotyping.

Application	Species	Genes	Forward	Reverse
qPCR	Mouse	*Slc40a1*	5'-TACCAGAAGACCCCTGCTCT-3'	5'-CCATCTCGGAAAGTGCGGAA-3'
		Pepck	5'-TGTCTTCACTGAGGTGCCAG-3'	5'-CTGGATGAAGTTTGATGCCC-3'
		G6pase	5'-ACACCGACTACTACAGCAACA-3'	5'-CCTCGAAAGATAGCAAGAGTA-3'
		Ppara	5'-AAGACTACCTGCTACCGAAATG-3'	5'-AACATTGGGCCGGTTAAGA-3'
		Acox1	5'-CCTGATTCAGCAAGGTAGGG-3'	5'-TCGCAGACCCTGAAGAAATC-3'
		Cpt1a	5'-GGCATAAACGCAGAGCATTCCTG-3'	5'-CAGTGTCCATCCTCTGAGTAGC-3'
		Acadm	5'-CATTCCGGAAAGTTGCGGTG-3'	5'-GAATCACAGGCATTTGCCCC-3'
		Acads	5'-TTGCCGAGAAGGAGTTGGTC-3'	5'-AGGTAATCCAAGCCTGCACC-3'
		Acadl	5'-CATCGCAGAGAAACATGGCG-3'	5'-TGGCTATGGCACCGATACAC-3'
		Srebf1	5'-GCCGTGGTGAGAAGCGCACAGCCC-3'	5'-CAAGACAGCAGATTTATTCAGCTTTGC-3'
		Acc1	5'-CCTCCGTCAGCTCAGATACA-3'	5'-TTTACTAGGTGCAAGCCAGACA-3'
		Fasn	5'-AACCTGGCCATGGTTTTGAG-3'	5'-GCCTGCGCTGTTCACATATA-3'
		Nr1h3	5'-AAGCCCTGCATGCCTACGT-3'	5'-TGCAGACGCAGTGCAAACA-3'
		Pparg	5'-TTCGCTGATGCACTGCCTATGA-3'	5'-AAGGAATGCGAGTGGTCTTCCA-3'
		Pgc1a	5'-CTCTGGAACTGCAGGCCTAA-3'	5'-TGCCTTGGGTACCAGAACA-3'
		Nrf1	5'-GGAGCACTTACTGGAGTCC-3'	5'-CTGTCCGATATCCTGGTGGT-3'
		Tfam	5'-GCAAAGGATGATTCGGCTCAGGGA-3'	5'-CCGGATCGTTTCACACTTCGACGG-3'
		Mtco1	5'-CTACTATTCGGAGCCTGAGC-3'	5'-GCATGGGCAGTTACGATAAC-3'
		Mtco2	5'-CCATAGGGCACCAATGATACTG-3'	5'-AGTCGGCCTGGGATGGCATC-3'
		Sdhb	5'-CAGAGTCGGCCTGCAGTTTC-3'	5'-GGTCCCATCGGTAAATGGCA-3'
		Atp5o	5'-TCTCGACAGGTTCGGAGCTT-3'	5'-AGAGTACAGGGCGGTTGCATA-3'
		Cox5b	5'-TTCAAGGTTACTTCGCGGAGT-3'	5'-CGGGACTAGATTAGGGTCTTCC-3'
		Cycs	5'-CCAAATCTCCACGGTCTGTTC-3'	5'-ATCAGGGTATCCTCTCCCCAG-3'
		Ndufs1	5'-AGGATATGTTCGCACAACTGG-3'	5'-TCATGGTAACAGAATCGAGGGA-3'
		Ndufv2	5'-GCAAGGAATTTGCATAAGACAGC-3'	5'-TAGCCATCCATTCTGCCTTTG-3'
		Il-10	5'-CTGGACAACATACTGCTAACCG-3'	5'-GGGCATCACTTCTACCAGGTAA-3'
		Il-4	5'-TCGGCATTTTGAACGAGGTC-3'	5'-GAAAAGCCCGAAAGAGTCTC-3'
		Arg1	5'-TTTTTCCAGCAGACCAGCTT-3'	5'-AGAGATTATCGGAGCGCCTT-3'
		Mrc1	5'-CTCGTGGATCTCCGTGACAC-3'	5'-GCAAATGGAGCCGTCTGTGC-3'
		Tnfa	5'-AGGGTCTGGGCCATAGAACT-3'	5'-CCACCACGCTCTTCTGTCTAC-3'
		Il-1b	5'-CATCCAGCTTCAAATCTCGCAG-3'	5'-CACACACCAGCAGGTTATCATC-3'
		Il-6	5'-CCACGGCCTTCCCTACTTC-3'	5'-TTGGGAGTGGTATCCTCTGTGA-3'
		Ccl2	5'-ATTGGGATCATCTTGCTGGT-3'	5'-CCTGCTGTTCACAGTTGCC-3'
		Fetua	5'-CACACTGGAGACCACTTGCCAT-3'	5'-GCCGTCTTGTTTCAGGATGTGG-3'
		Lect2	5'-GGACGTGTGACAGCTATGGC-3'	5'-TCCCAGTGAATGGTGCATACA-3'
		Angptl6	5'-ACTACGACAGCTTCTCCTTG-3'	5'-AGTGCTGAAAGGTTTGTCAT-3'
		Rbp4	5'-GACAAGGCTCGTTTCTCTGG-3'	5'-AAAGGAGGCTACACCCCAGT-3'
		Fetub	5'-CACCAGTTGCTCCTGTGAGT-3'	5'-CAGAGCACCAGAAGTCGGAG-3'
		Rna18s	5'-GTAACCCGTTGAACCCCATT-3'	5'-CCATCCAATCGGTAGTAGCG-3'
		Actb	5'-CTGAGAGGGAAATCGTGCGT-3'	5'-TGTTGGCATAGAGGTCTTTACGG-3'
		Acsl4	5'-CCTTTGGCTCATGTGCTGGAAC-3'	5'-GCCATAAGTGTGGGTTTCAGTAC-3'
		Alox12	5'-CTCTTGTCATGCTGAGGATGGAC-3'	5'-AAGAGCCAGGCAAGTGGAGGAT-3'
		Alox15	5'-GACACTTGGTGGCTGAGGTCTT-3'	5'-TCTCTGAGATCAGGTCGCTCCT-3'
		Lpcat3	5'-CCATCTCTTCCACACCTTCACG-3'	5'-GGATGAGGAACTGAAGCACGAC-3'
		Ftmt	5'-GCTTCCTCTCAGGACTCCACTA-3'	5'-TGGACAGGTACACGTAGGATGC-3'
		Senp3	5'-CTTATGGCAGCCTCATCCCTCT-3'	5'-TTGCCTGGCATCCGCTGATAAG-3'
		Bnip3	5'-GCTCCAAGAGTTCTCACTGTGAC-3'	5'-GTTTTTCTCGCCAAAGCTGTGGC-3'
ChIP	Human	*FETUA*	5'- GGGACCAGCGATGTCCTAAC-3'	5'-TAGACCCATGGAGGGTGGTG-3'
		LECT2	5'-TATTGATGGATGCTTTTAATGGTGT-3'	5'-AGGAGAATGTTCGTGCGAATG-3'
Genotyping	Mouse	*Fpn-flox*	5'-GGCATTCCCAACACTTTAGC-3'	5'-CCCATAGGTTAAACTGCTTCAA-3'
		Albumin-Cre	5'-GCGGTCTGGCAGTAAAAACTATC-3'	5'-GTGAAACAGCATTGCTGTCACTT-3'

Supplemental Table 5. Information on primary antibodies used in Western blotting.

Primary antibodies	Source	Identifier
FPN	Alpha Diagnostic Intl.	#MTP11-A
p-AKT (S473)	Cell Signaling Technology	#4060
AKT	Cell Signaling Technology	#9272
GAPDH	Cell Signaling Technology	#2118
β-Actin	Sigma-Aldrich	#A5441
UCP1	Sigma-Aldrich	#U-6382
HSP90	Enzo Life Sciences	#ADI-SPA-836-F
FTH	Abcam	#ab65080
FTL	Abcam	#ab69090
Fetuin-A	Santa Cruz Biotechnology	#sc-166531
LECT2	Abcam	#ab119429
FOXO1	Cell Signaling Technology	#2880
p-NF-κB p65	Cell Signaling Technology	#3033
p-IκB	Cell Signaling Technology	#2859
IκB	Santa Cruz Biotechnology	#sc-371
p-ERK	Cell Signaling Technology	#4376
ERK	Cell Signaling Technology	#4695
p-AMPK	Cell Signaling Technology	#2535
AMPK	Cell Signaling Technology	#2603
p-ACC	Cell Signaling Technology	#3661
