# Supplementary material for: Iron overload in steatotic hepatocytes drives systemic metabolic dysfunction via alterations in hepatokine production
Source: J Clin Invest. 2026 Apr 28;136(12):e196374. doi: 10.1172/JCI196374 (PMC13262725; doi:10.1172/JCI196374)
Supplement: Unedited blot and gel images [file jci-136-196374-s285.pdf]

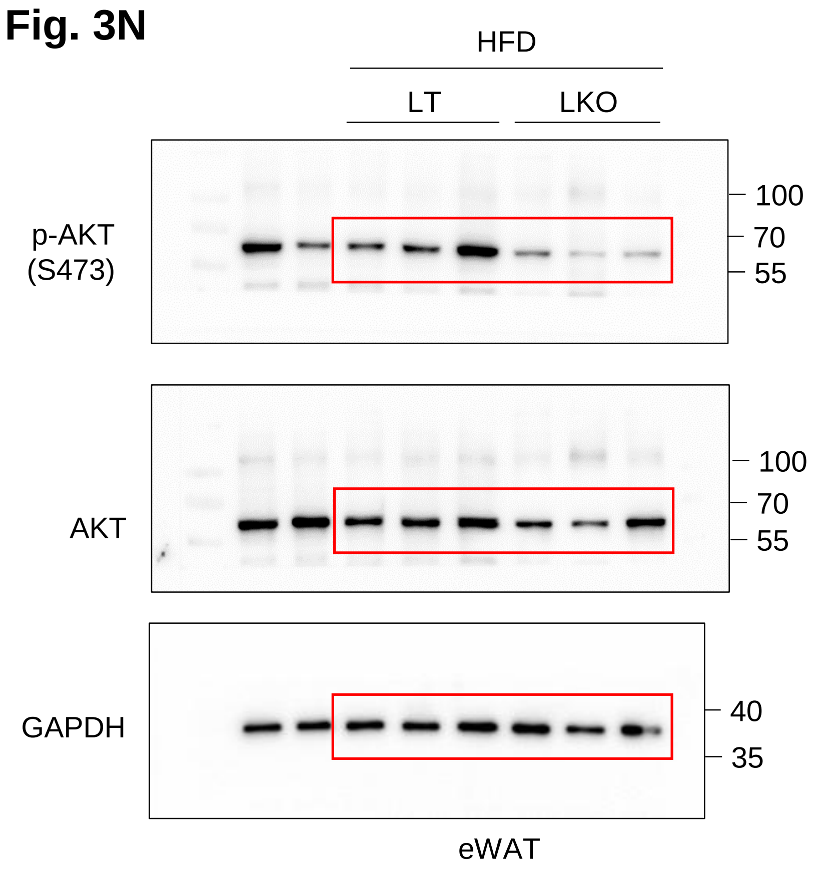

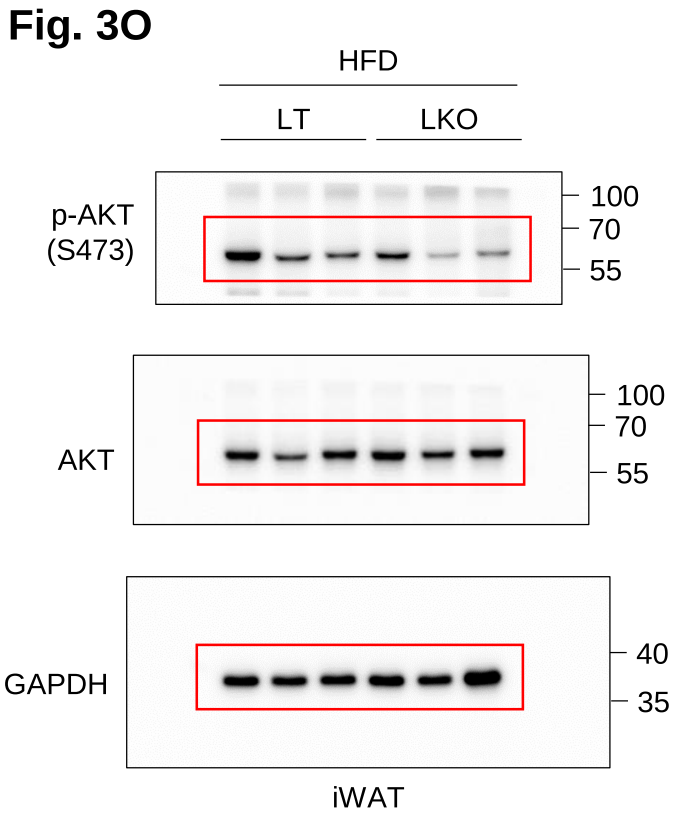

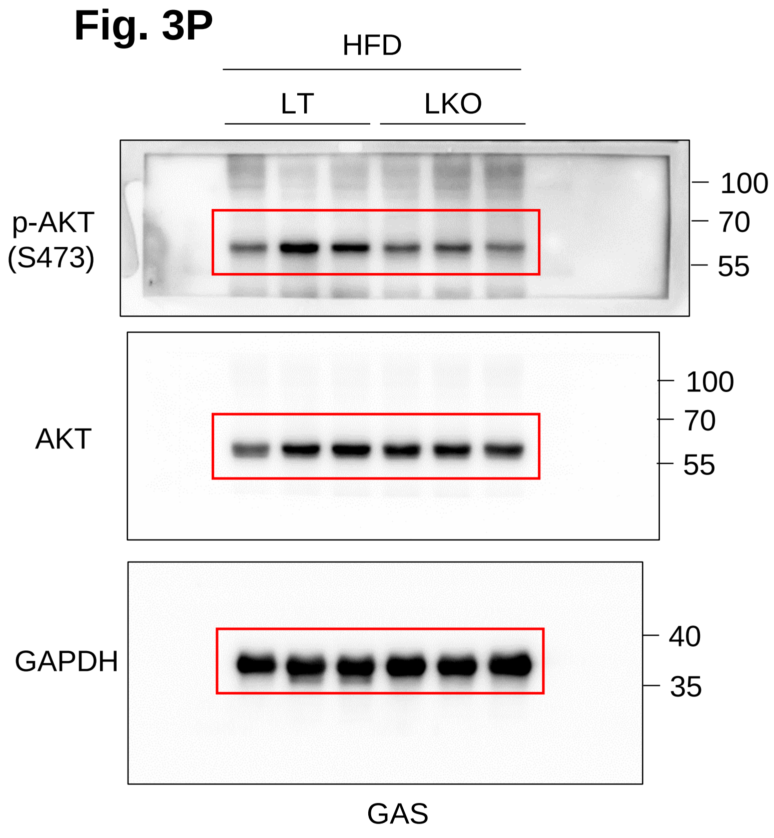

Figure 3 (raw blots)

Fig. 4G

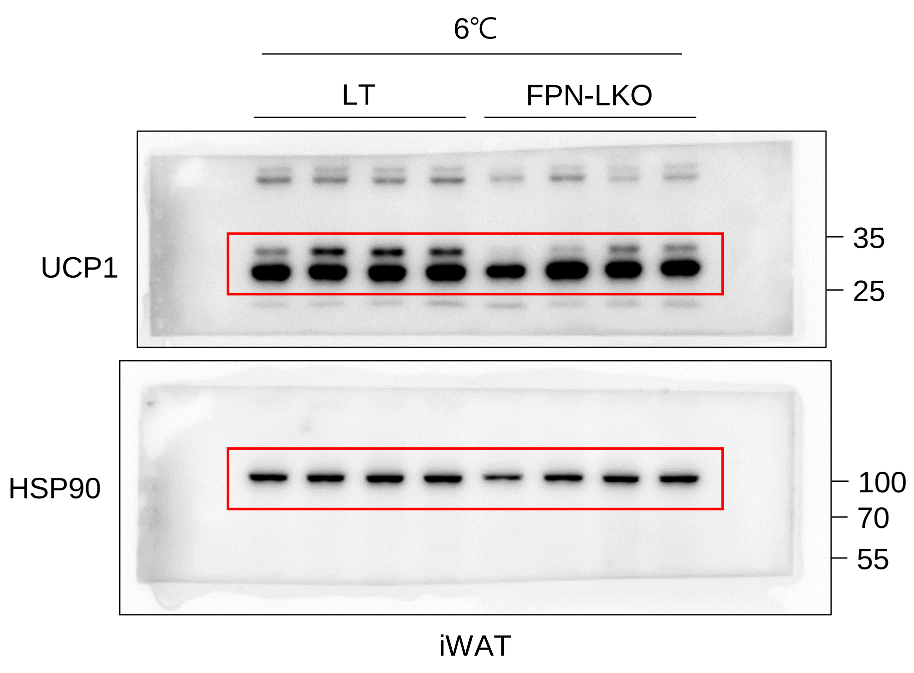

Figure 4 (raw blots)

Fig. 5C

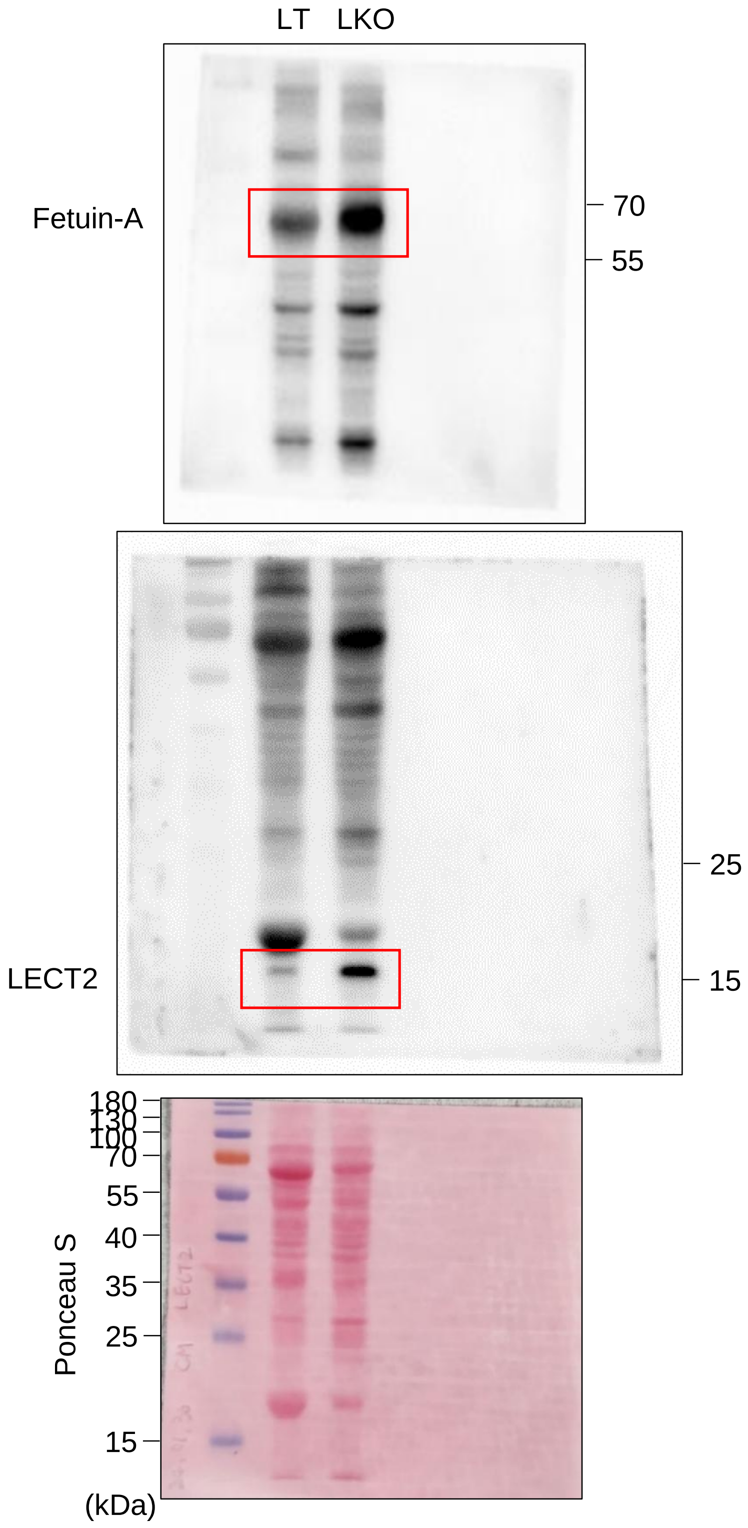

Figure 5 (raw blots)

Fig. 6D

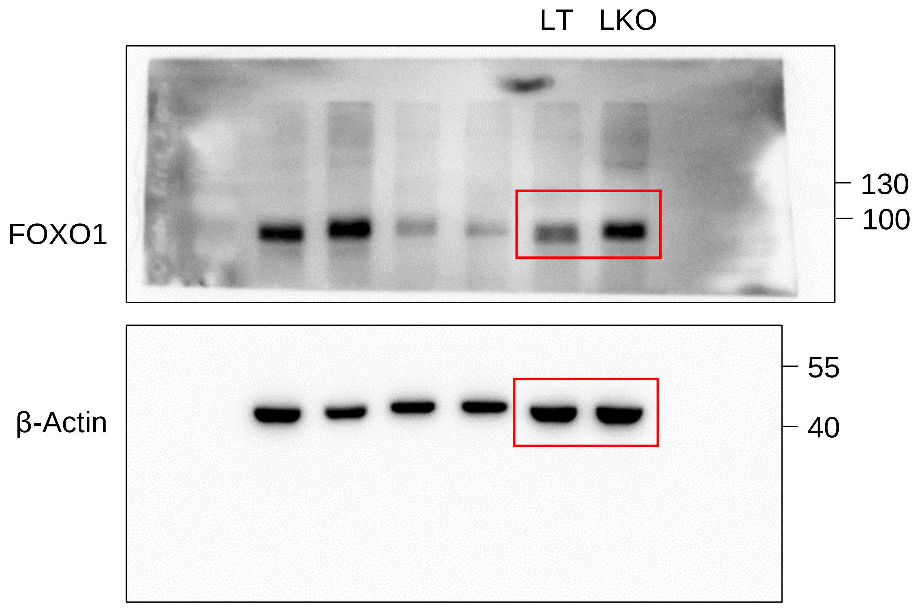

Fig. 6E

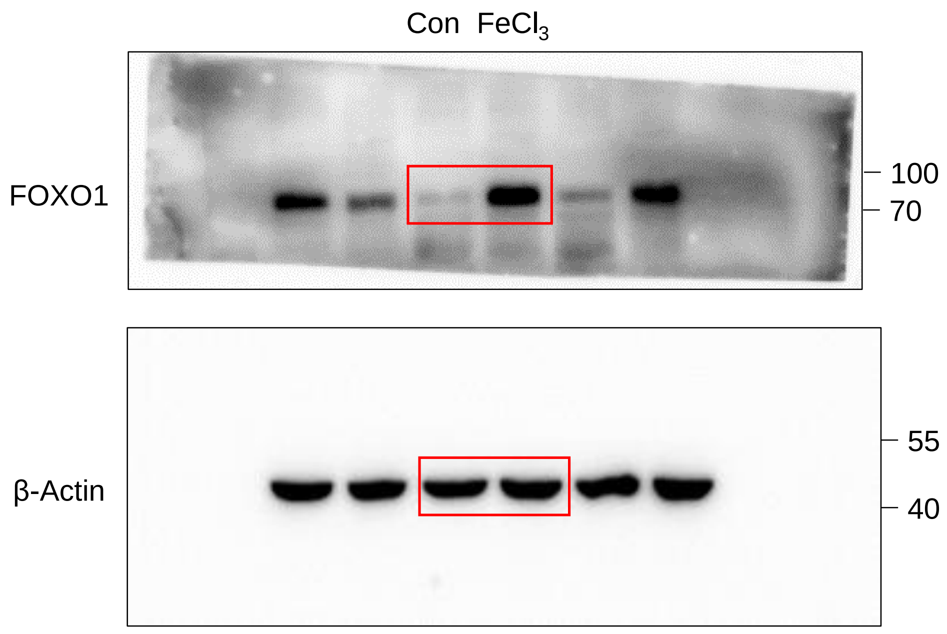

Fig. 6I

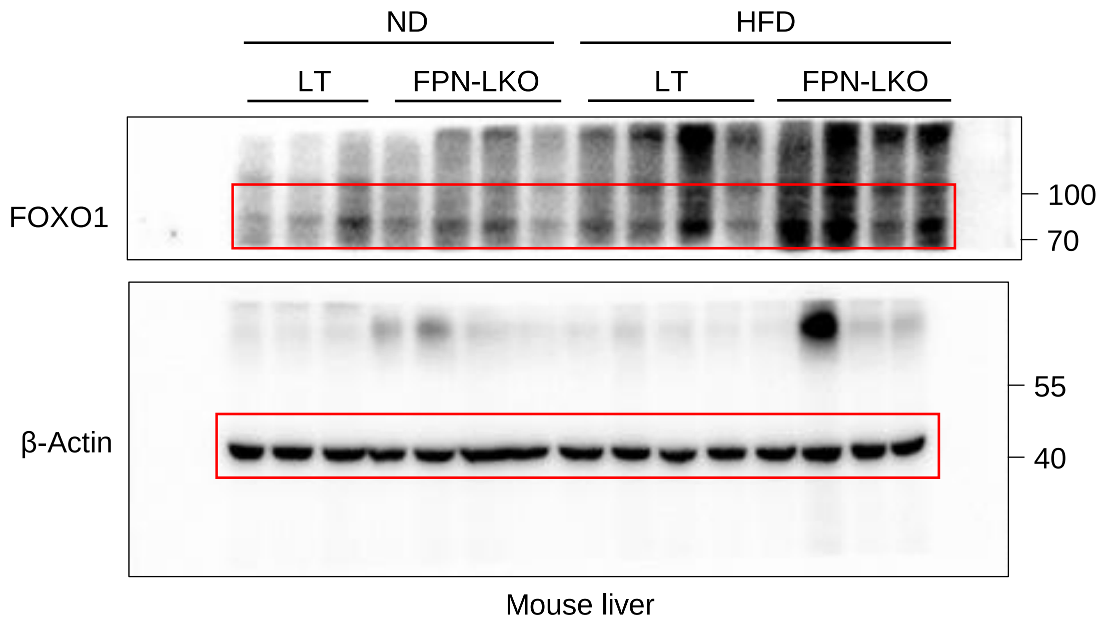

Figure 6 (raw blots)

Fig. 7H

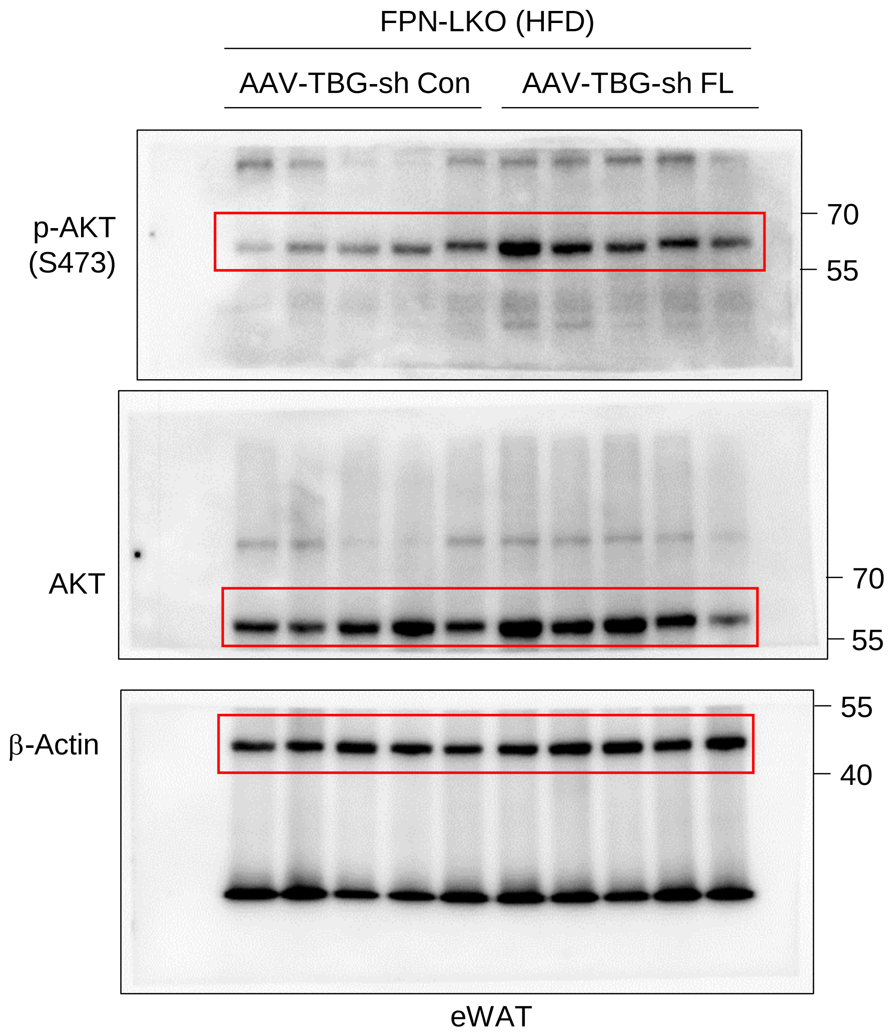

Fig. 7I

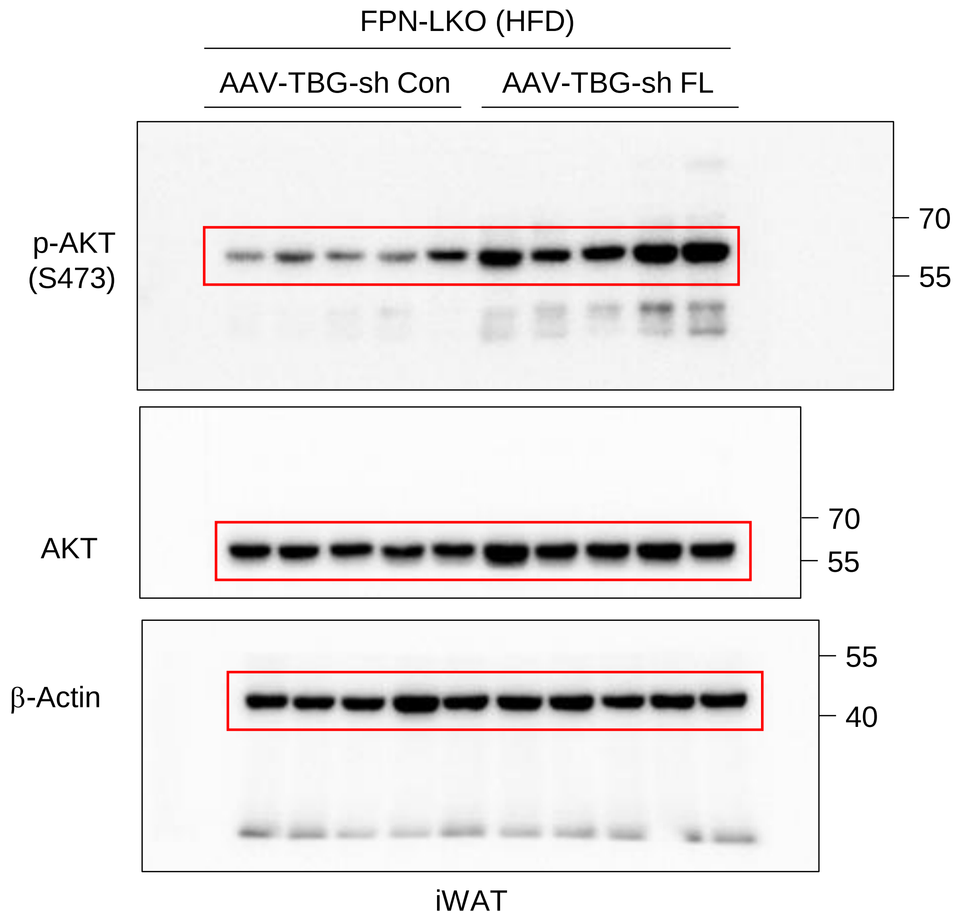

Fig. 7J

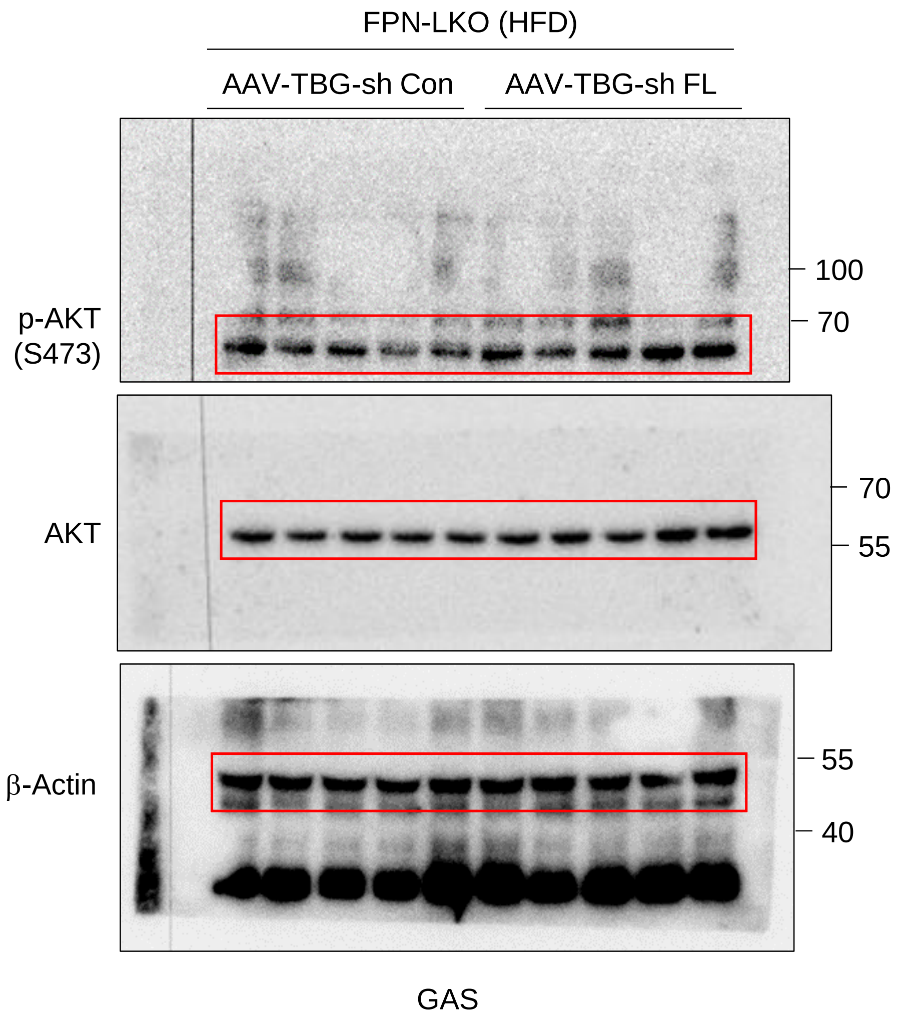

Figure 7 (raw blots)

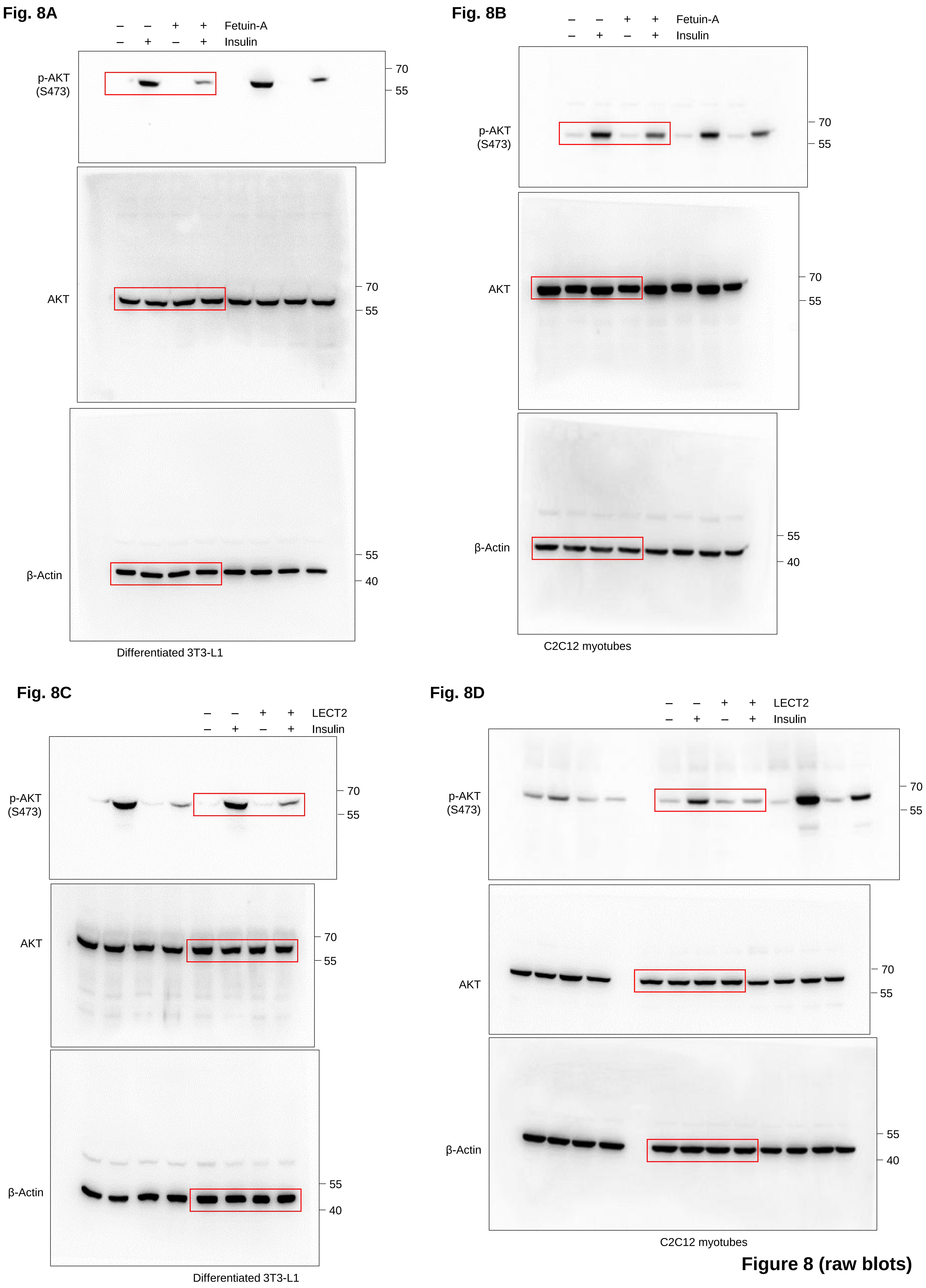

Figure 8 (raw blots)

Fig. 9F

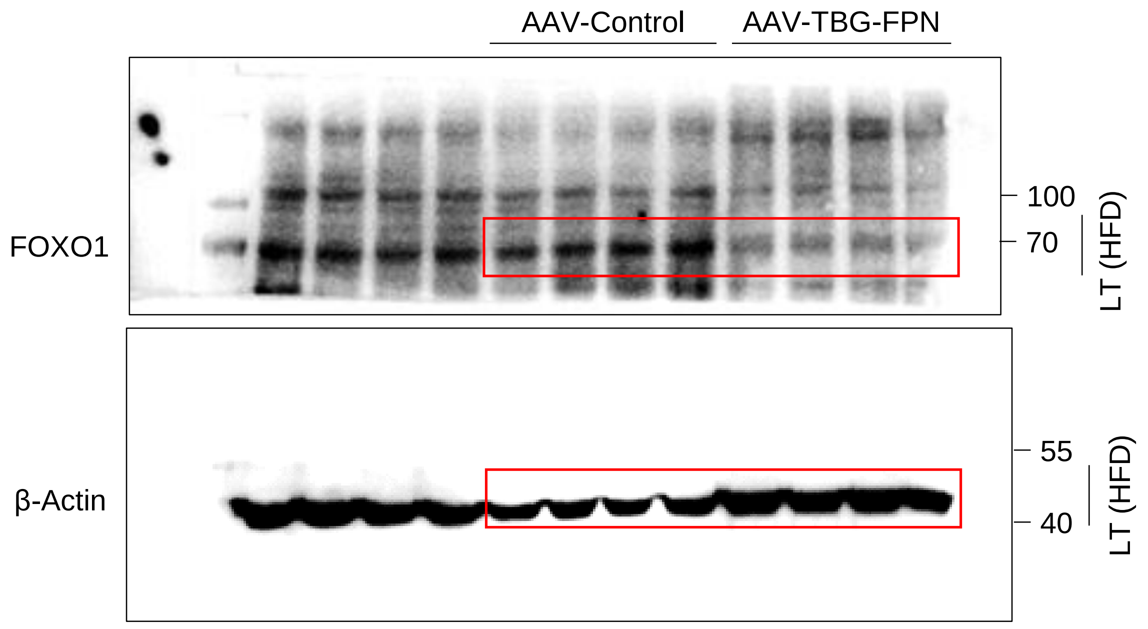

Fig. 9G

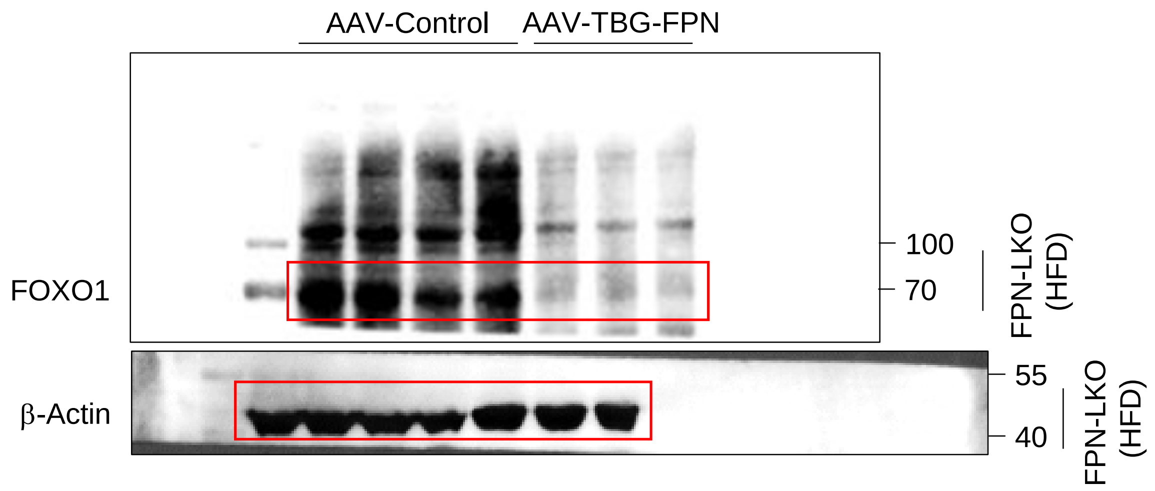

Figure 9 (raw blots)

Fig. 10C

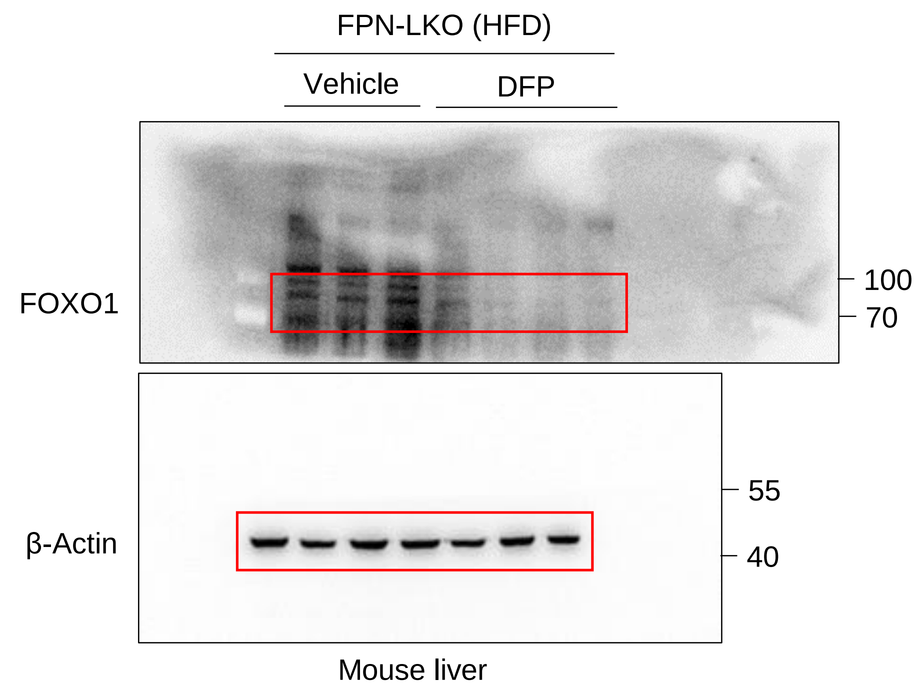

Fig. 10D

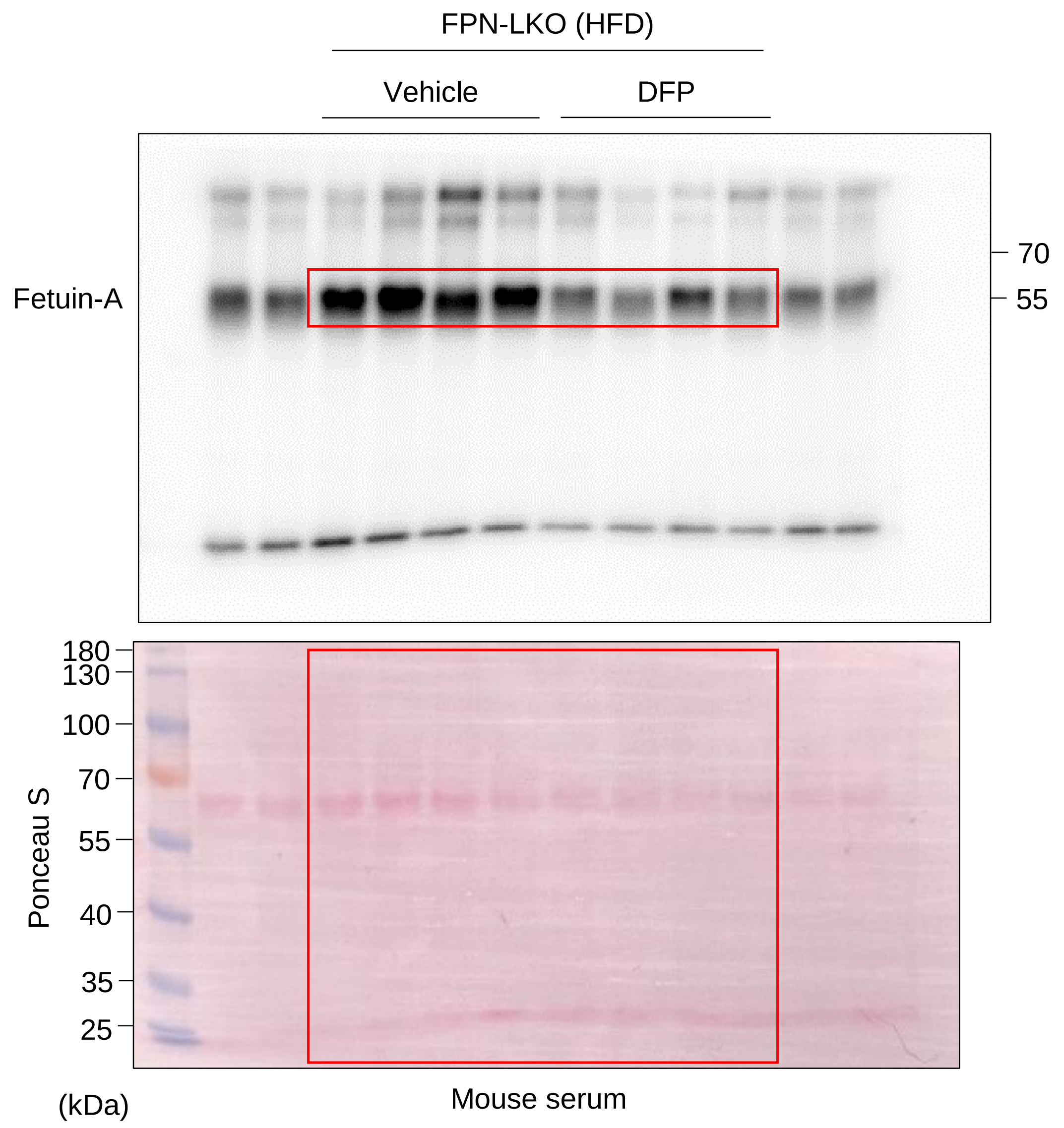

Figure 10 (raw blots)

Supplemental Fig. 3F

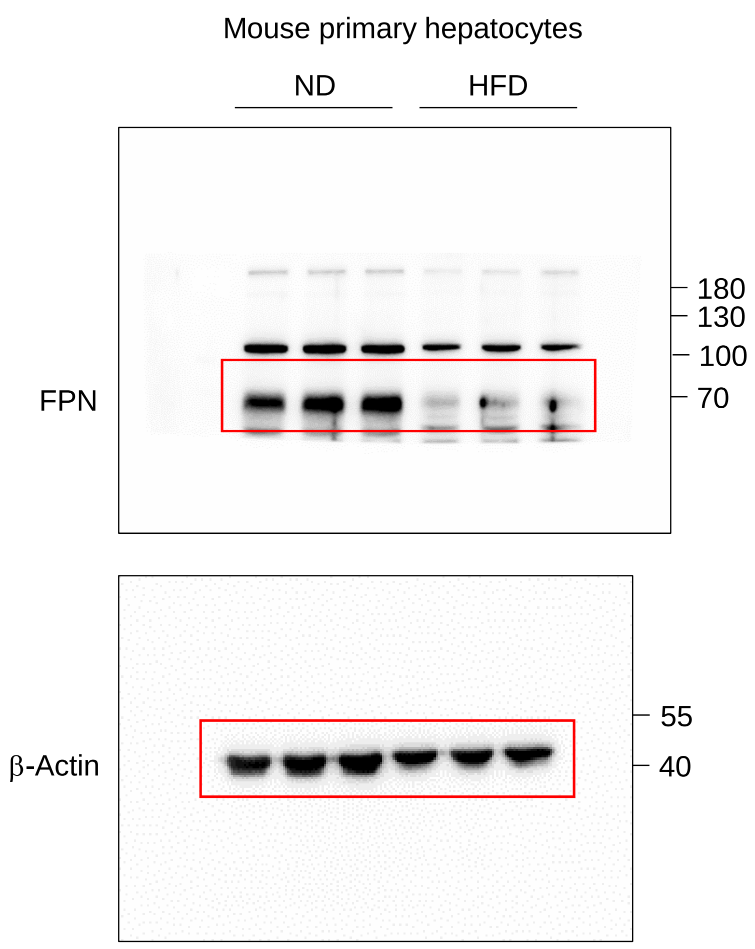

Supplemental Fig. 4C

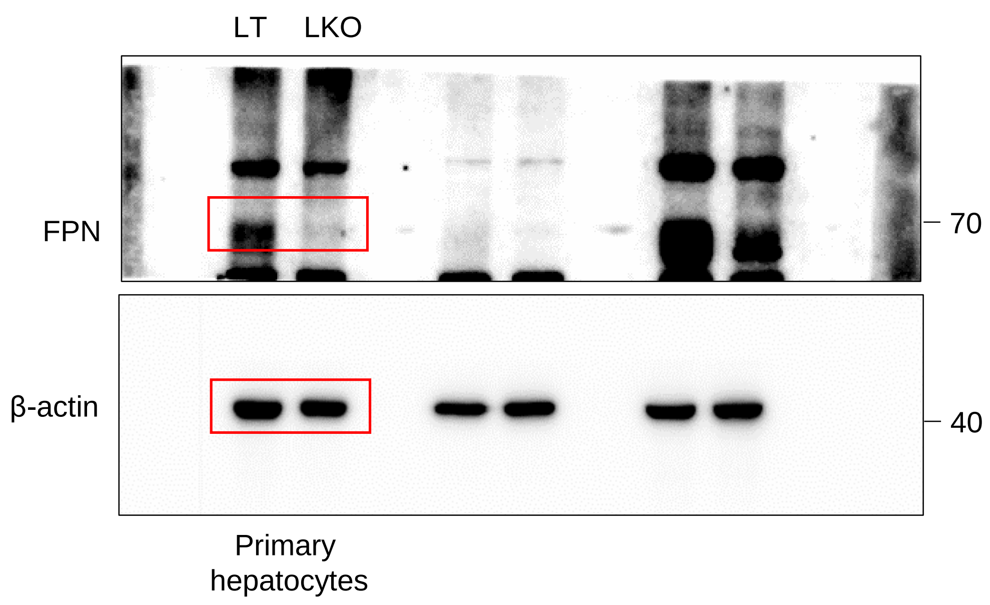

Supplemental Fig. 4E

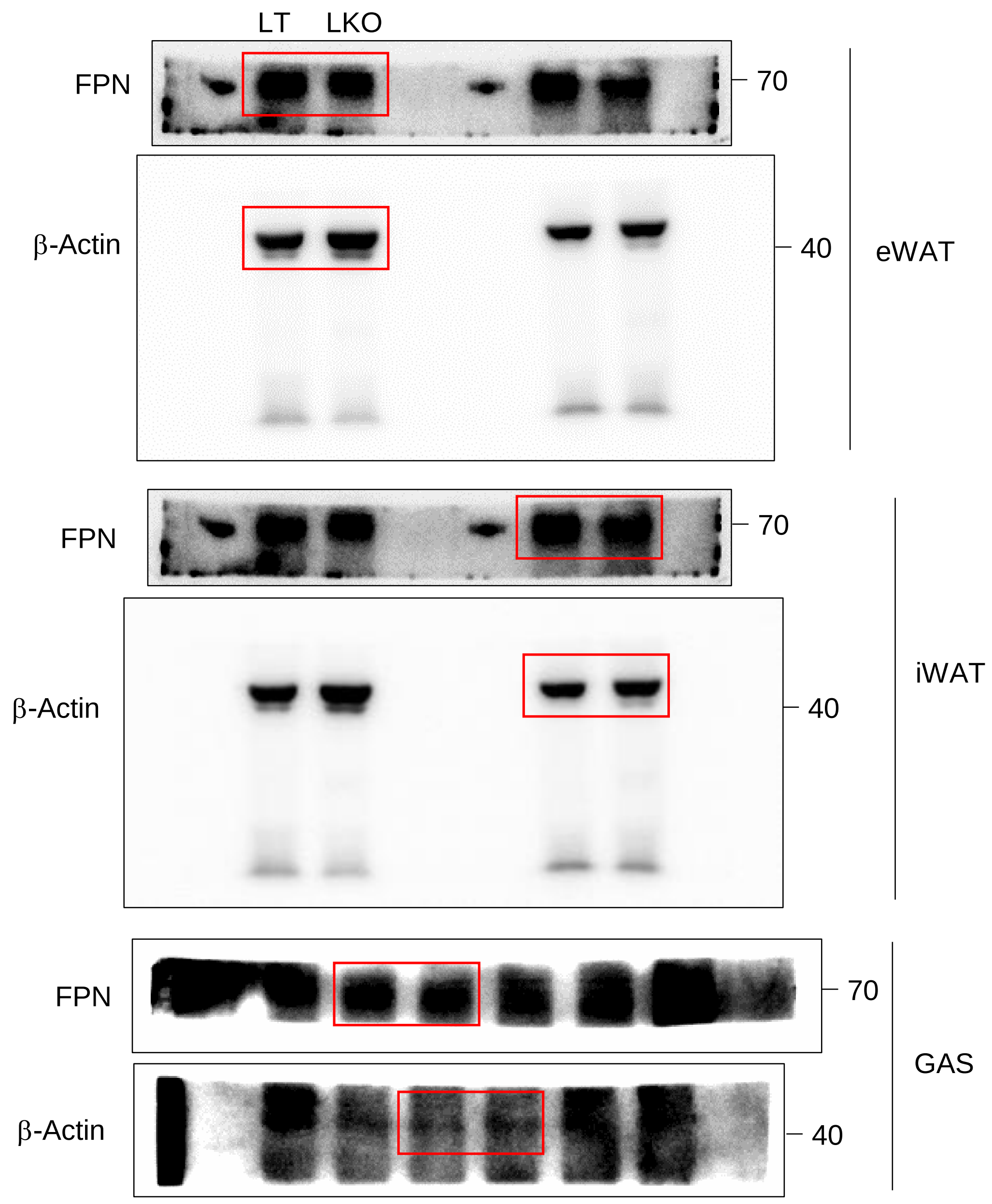

Supplemental Fig. 4 (raw blots)

Supplemental Fig. 5C

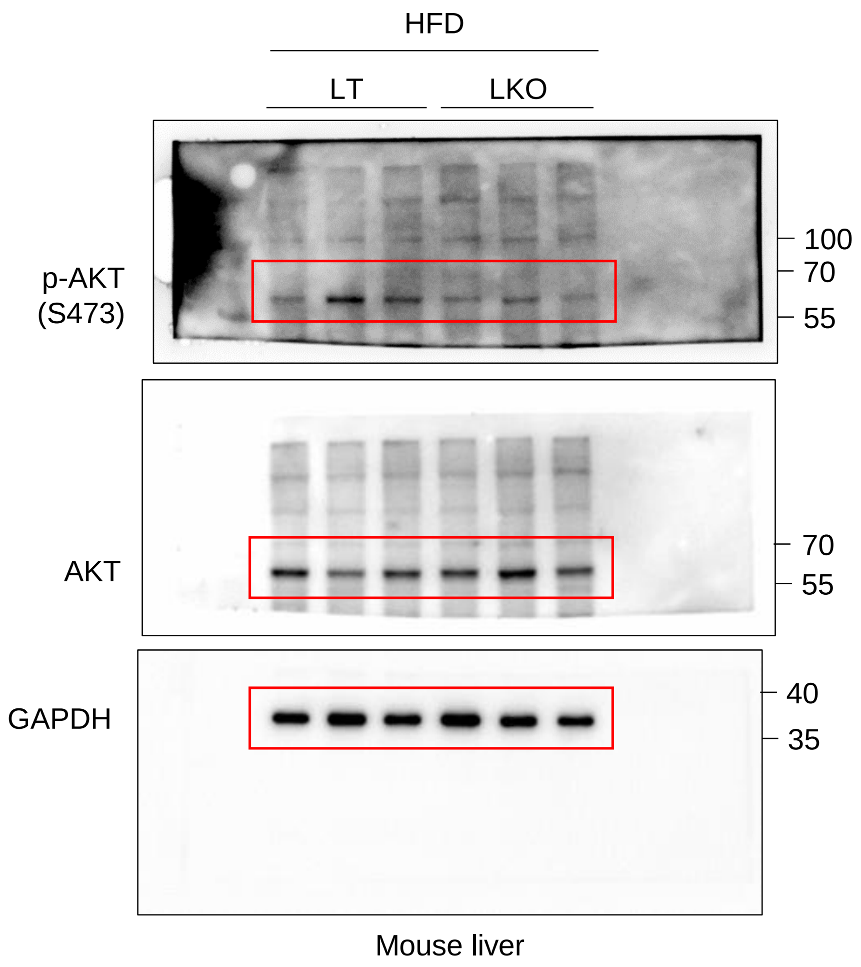

Supplemental Fig. 5E

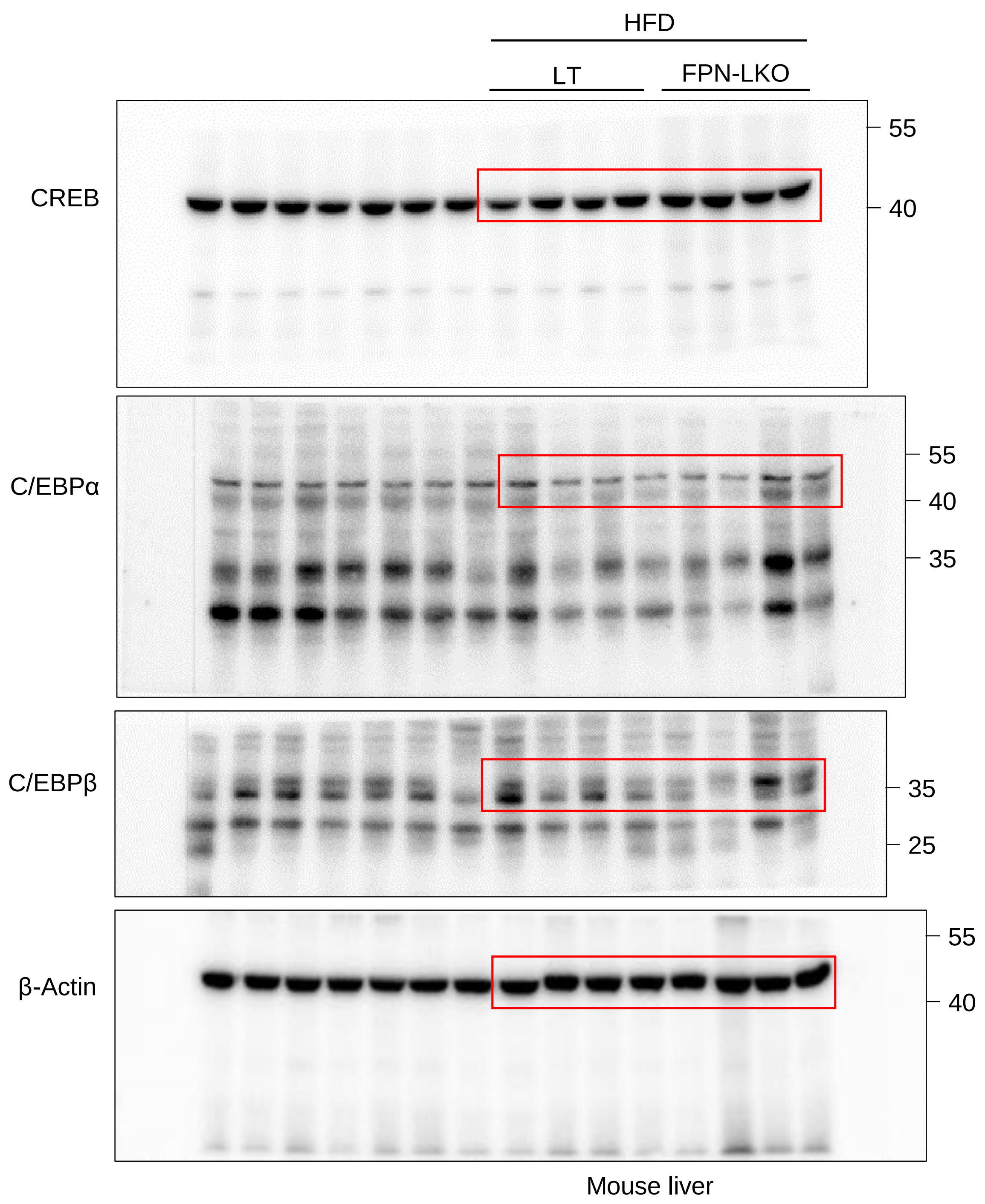

Supplemental Fig. 5 (raw blots)

Supplemental Fig. 8K

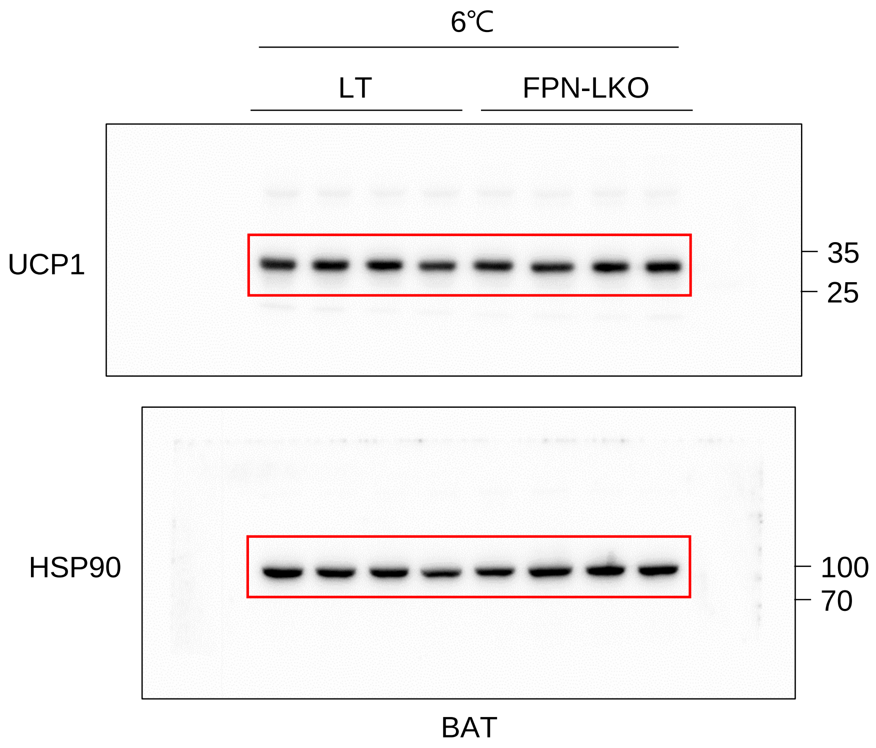

Supplemental Fig. 8L

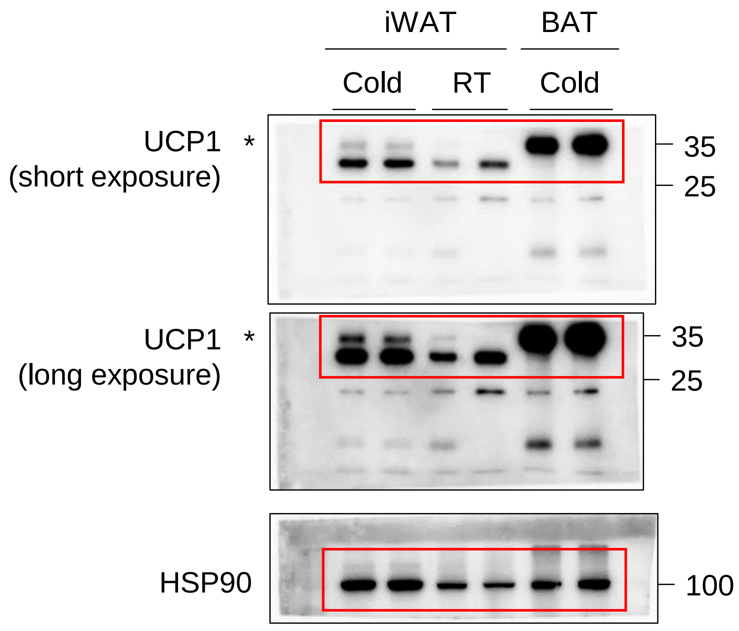

Supplemental Fig. 8 (raw blots)

Supplemental Fig. 9B

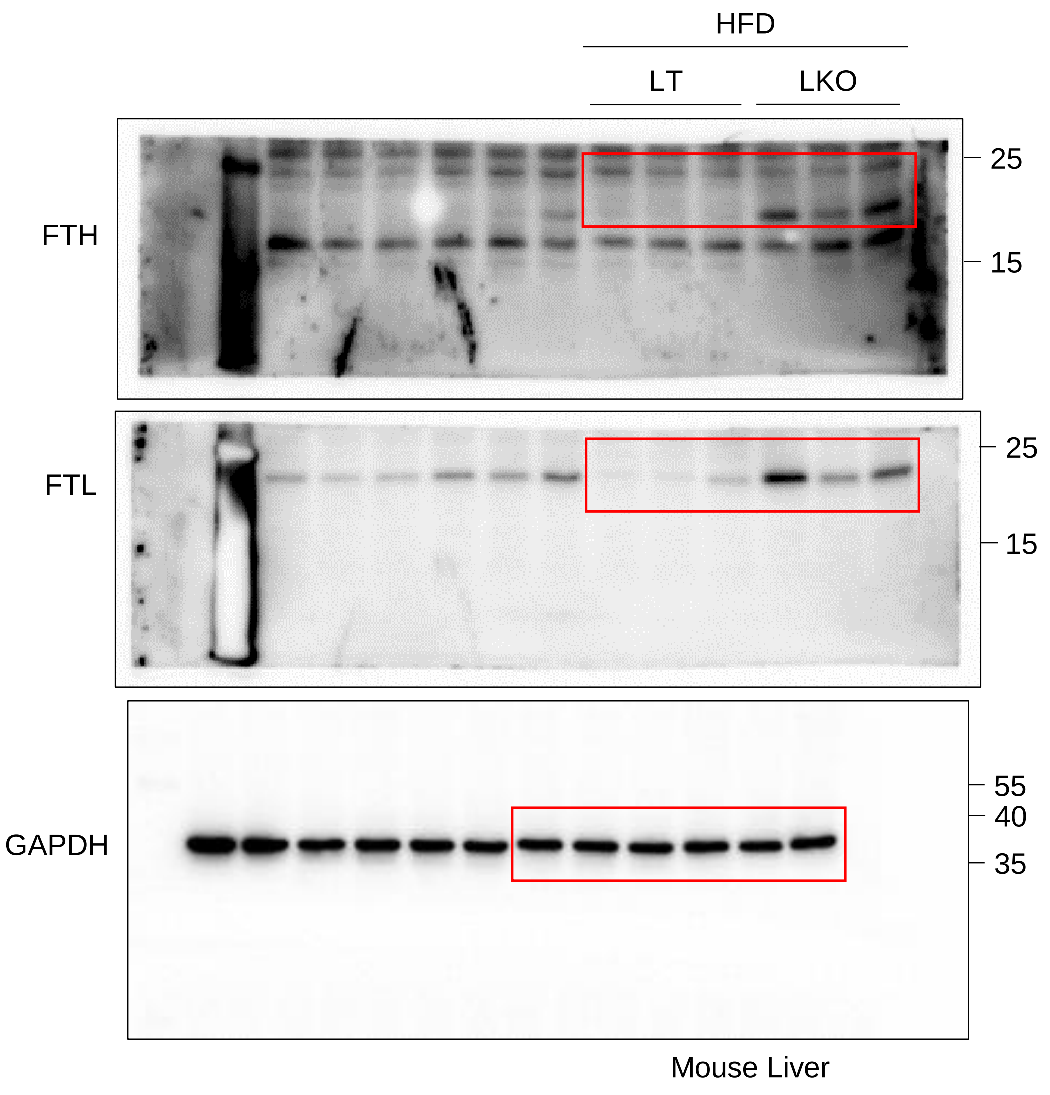

Supplemental Fig. 10B

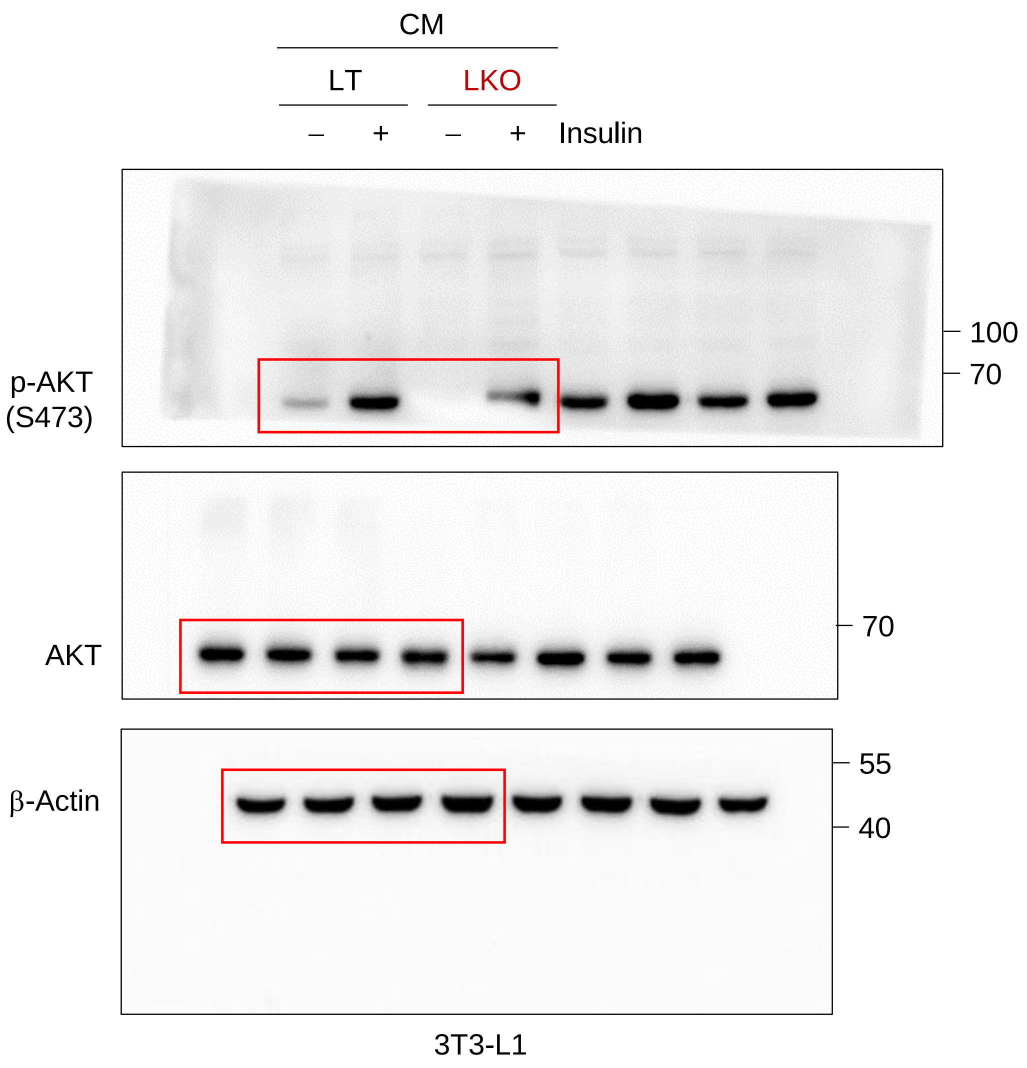

Supplemental Fig. 10C

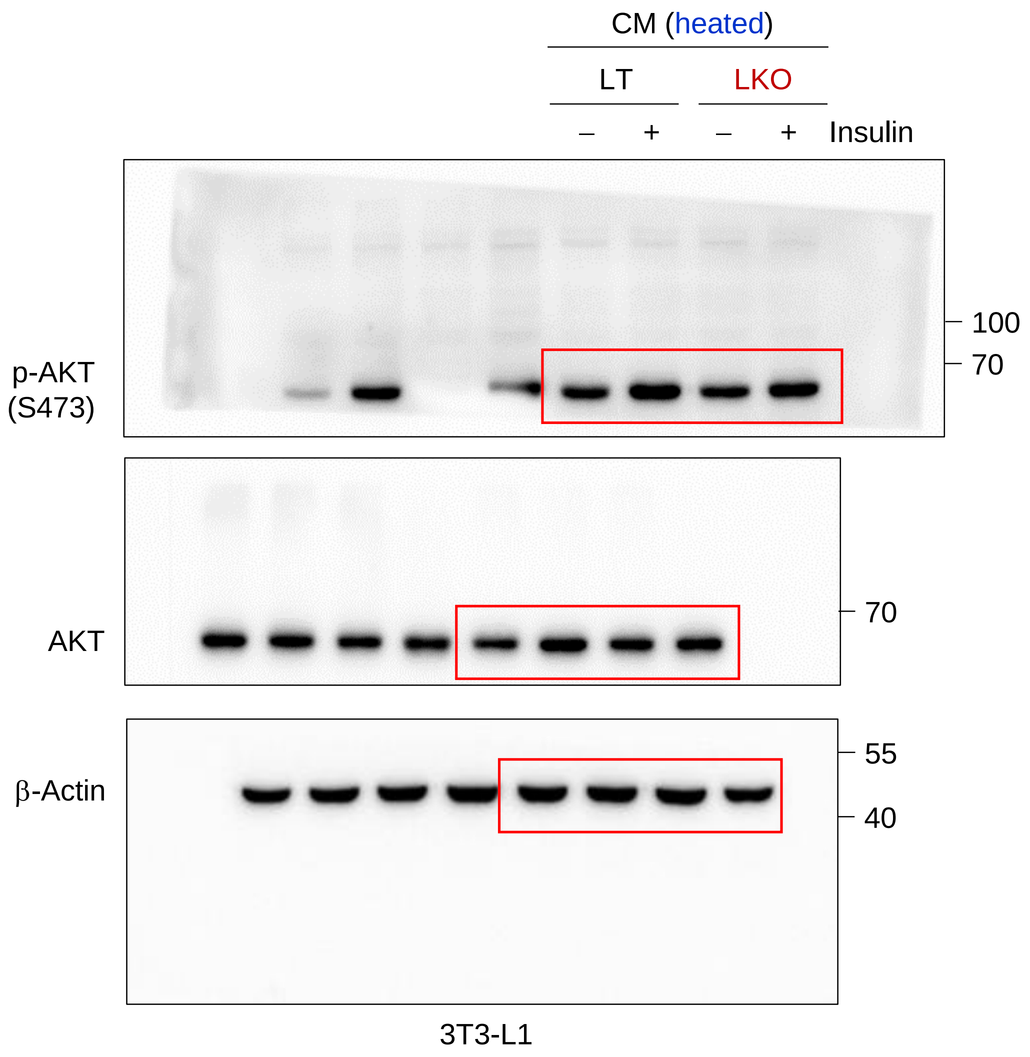

Supplemental Fig. 10D

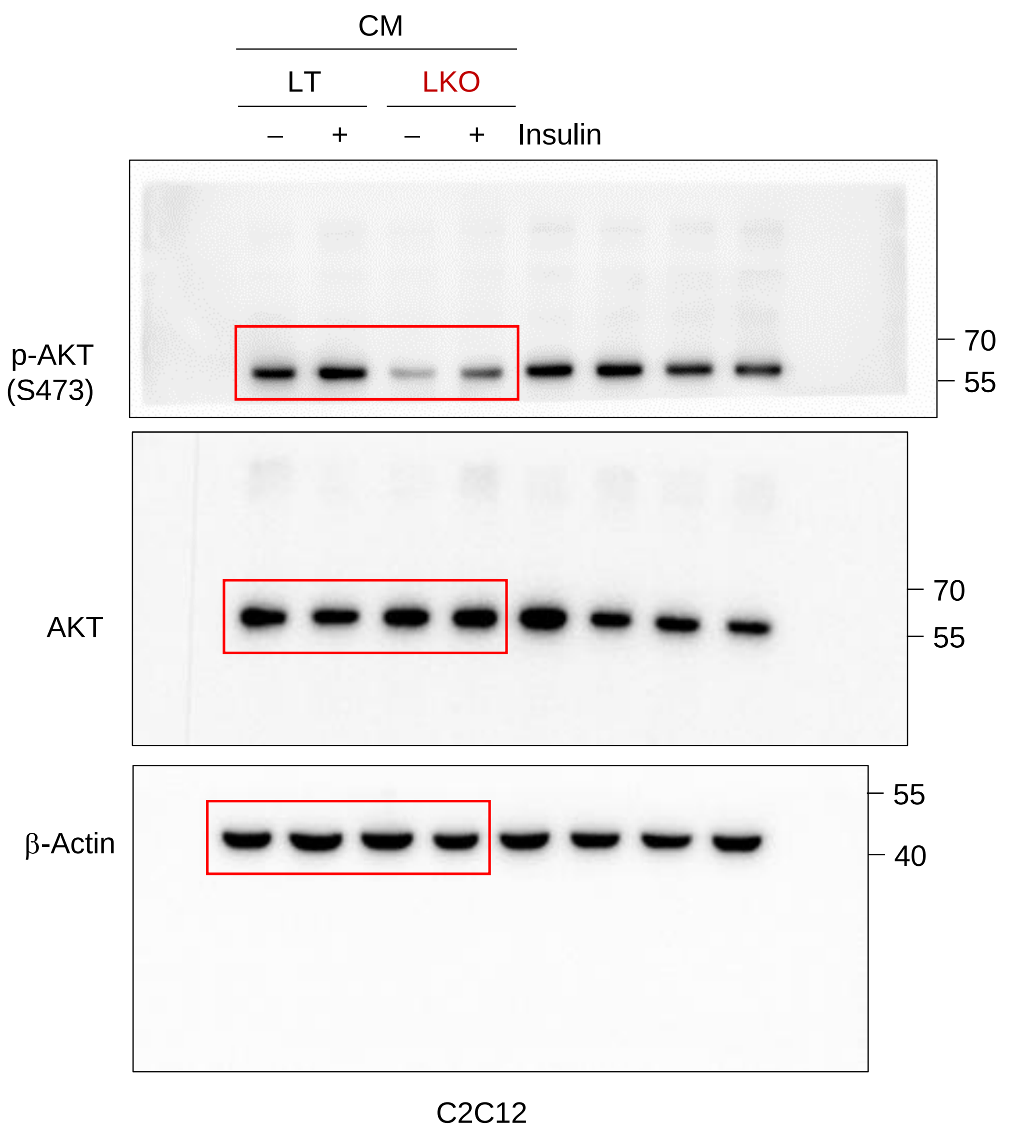

Supplemental Fig. 10E

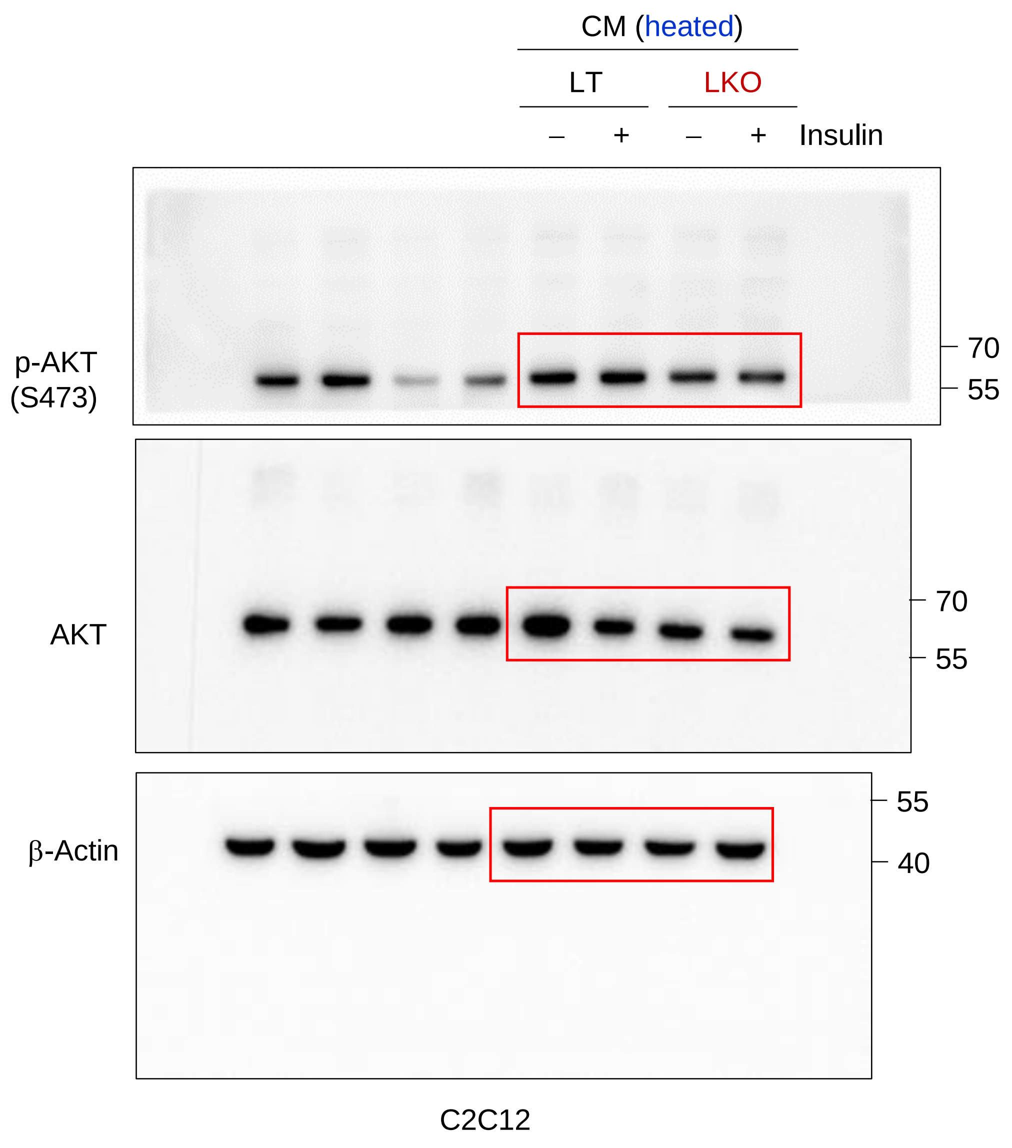

Supplemental Fig. 10 (raw blots)

Supplemental Fig. 11D

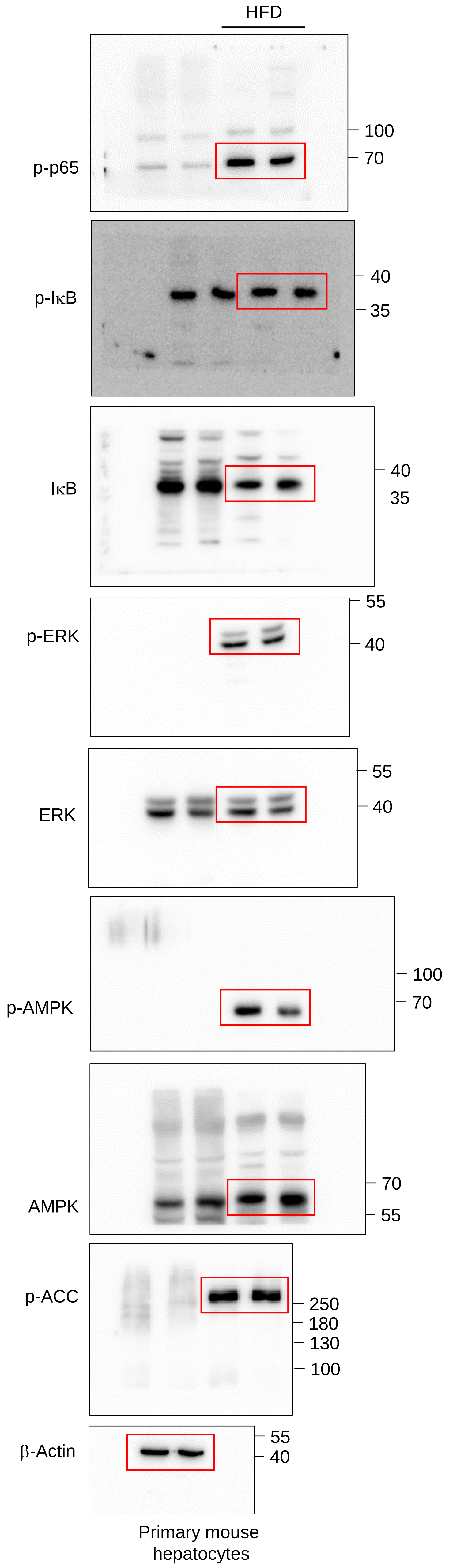

Supplemental Fig. 11E

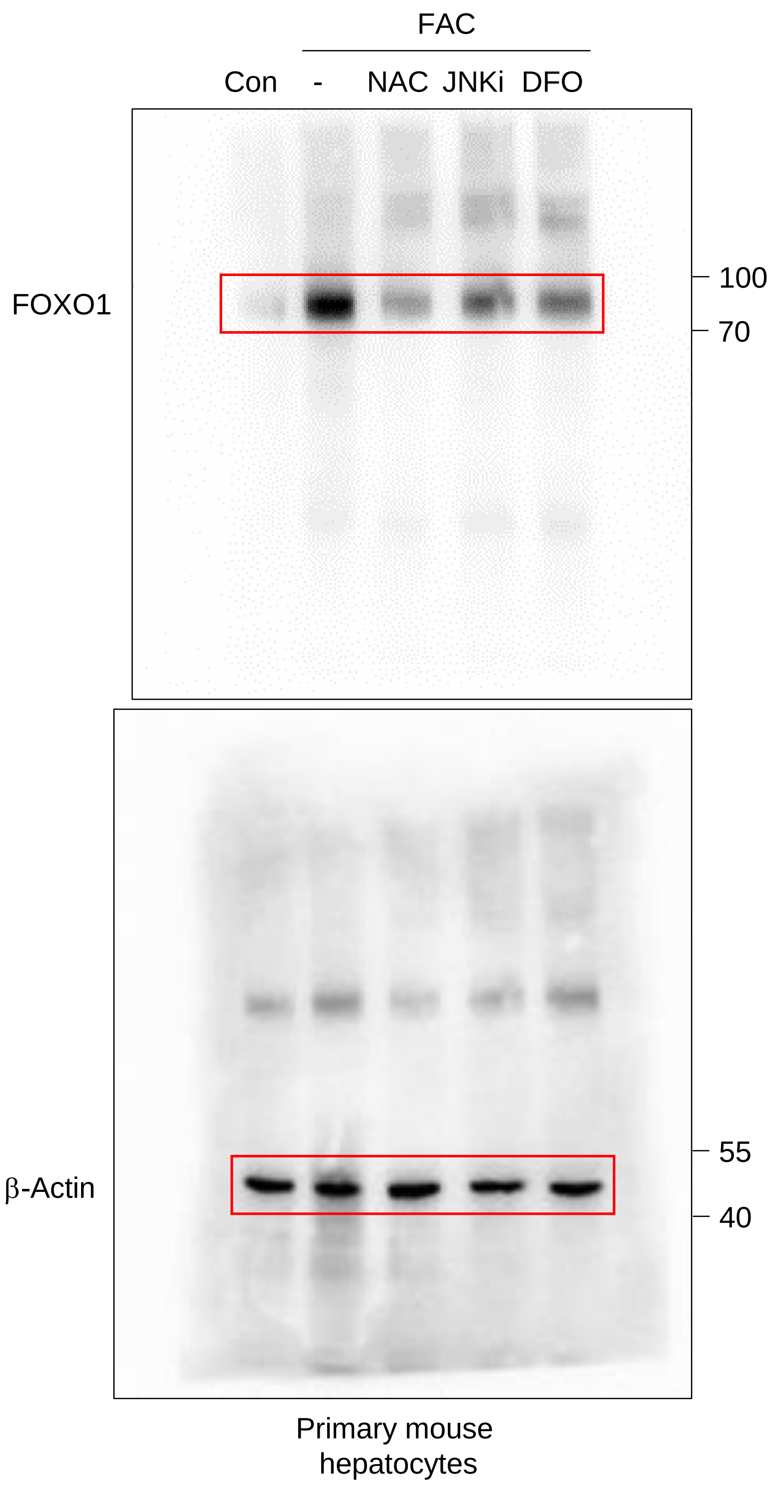

Supplemental Fig. 11F

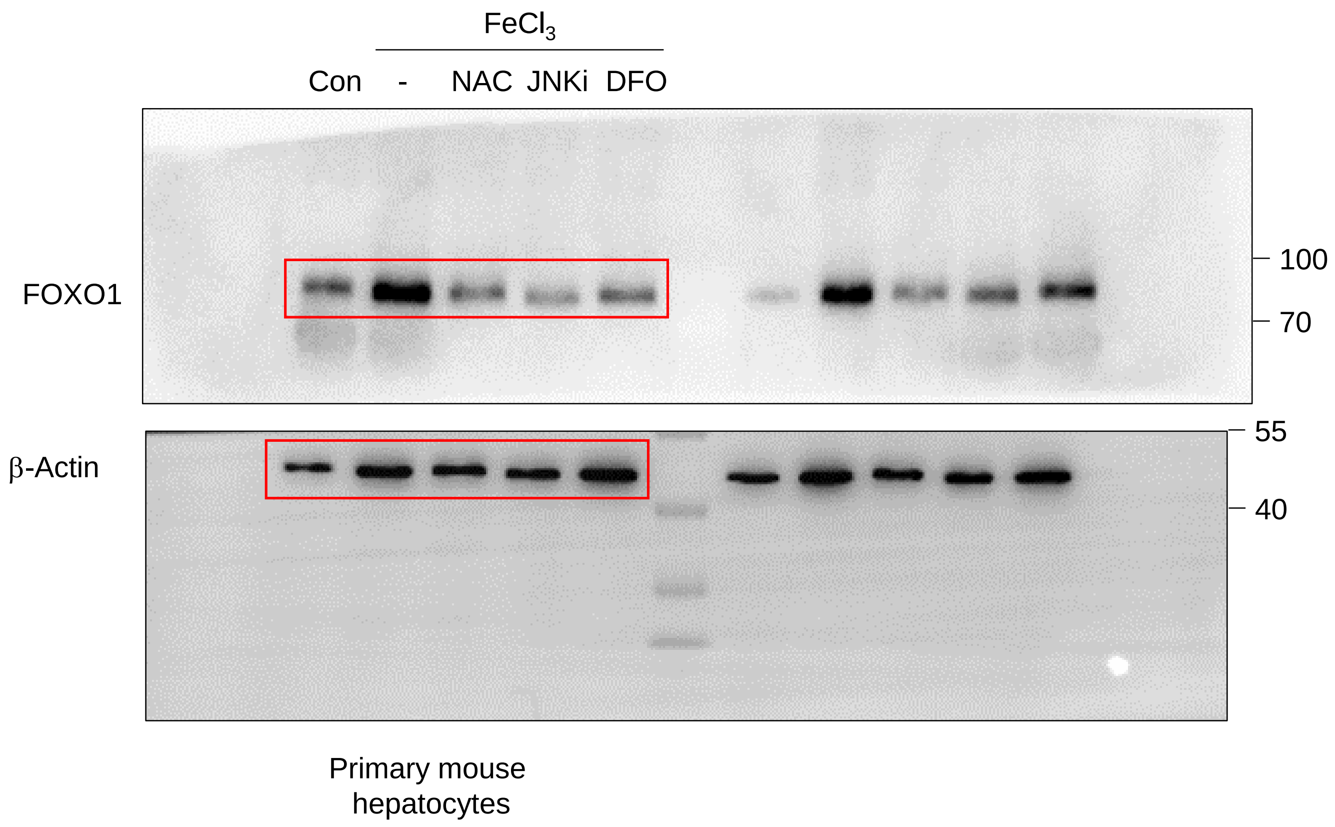

Supplemental Fig. 11 (raw blots)

Supplemental Fig. 12G

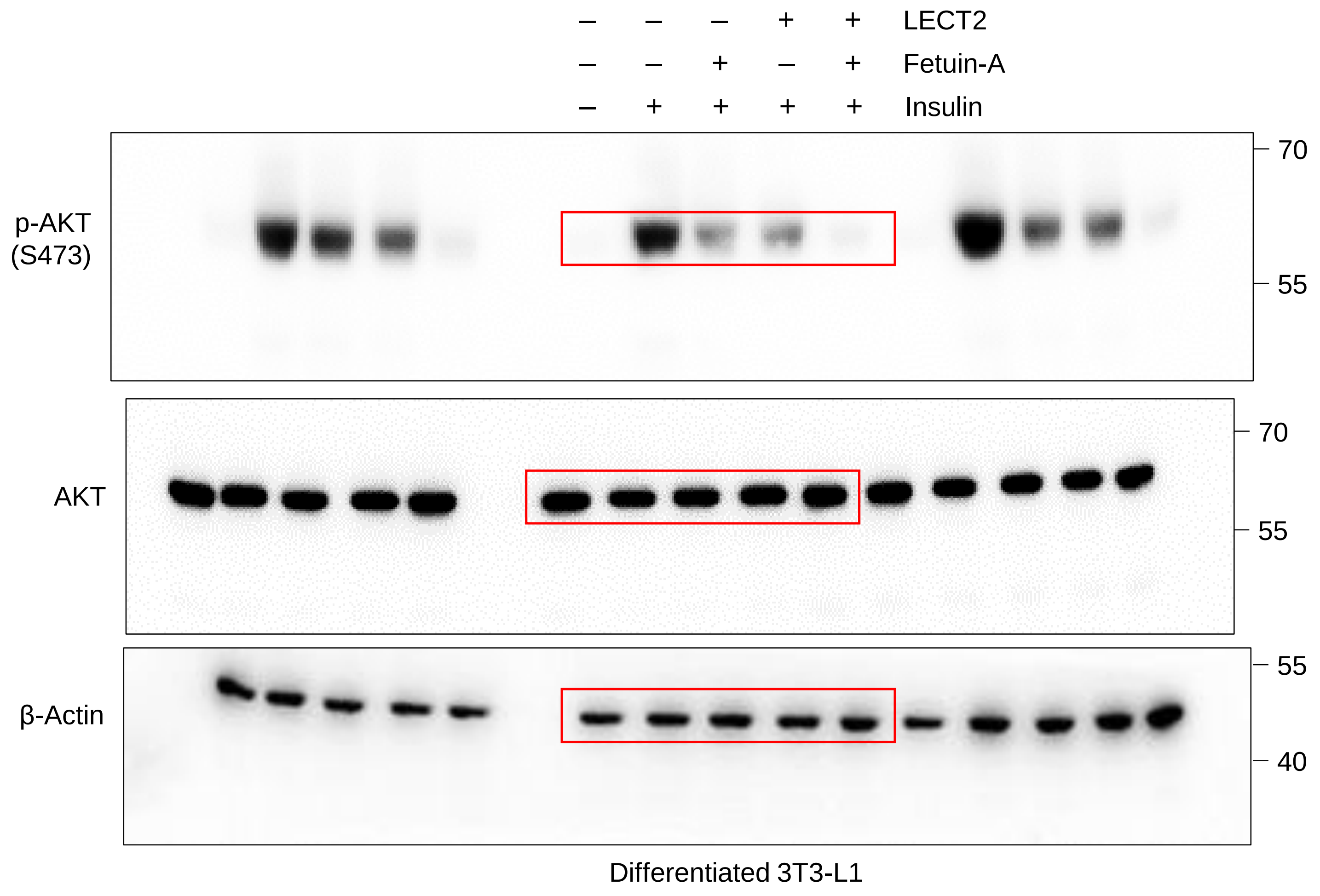

Supplemental Fig. 12I

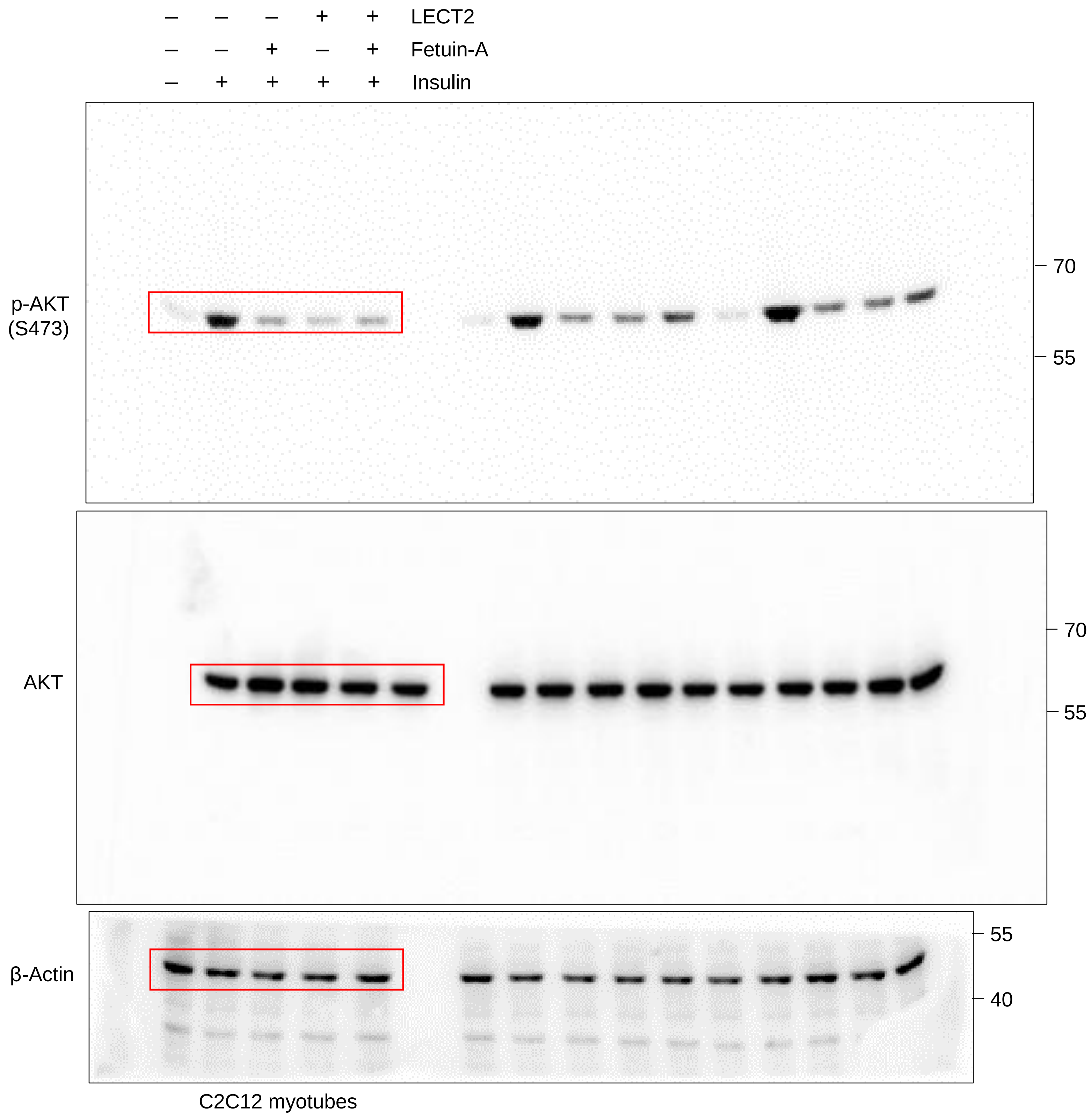

Supplemental Fig. 12 (raw blots)

Supplemental Fig. 13C

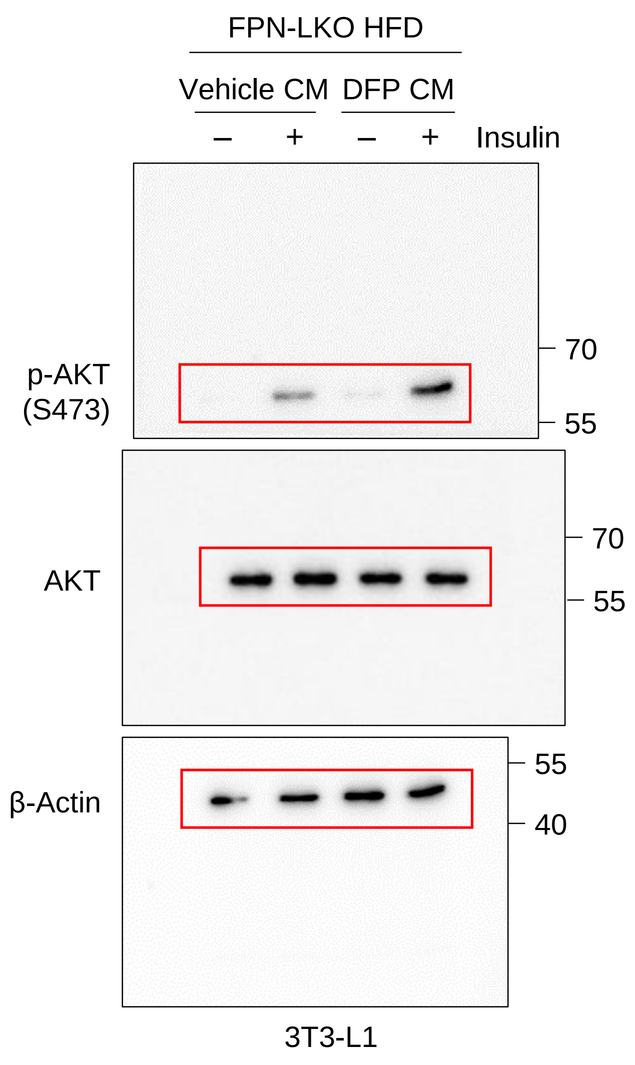

Supplemental Fig. 13D

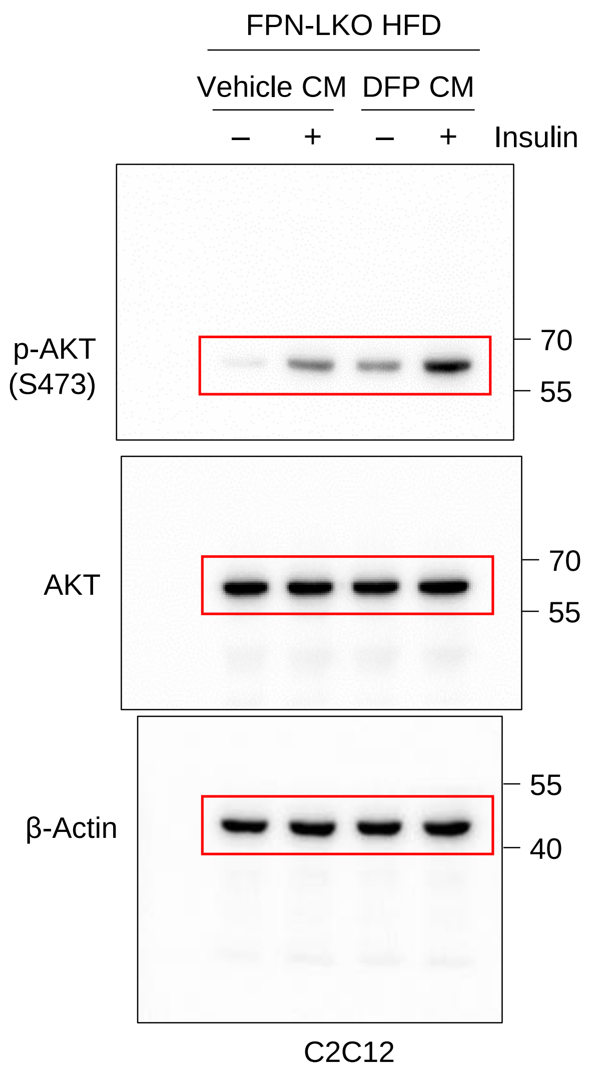

Supplemental Fig. 13 (raw blots)
